# Cell-type-focused compound screen in human organoids reveals CK1 inhibition protects cone photoreceptors from death

**DOI:** 10.1016/j.neuron.2026.02.024

**Published:** 2026-07-01

**Authors:** Stefan E. Spirig, Álvaro Herrero-Navarro, Larissa Utz, Valeria J. Arteaga-Moreta, Zoltan Raics, Susana Posada-Céspedes, Stephanie Chreng, Olaf Galuba, Inga Galuba, Isabelle Claerr, Steffen Renner, Miklos Boldogkoi, Verónica Moreno-Juan, P. Timo Kleindienst, Adrienn Volak, Jannick Imbach, Svitlana Malysheva, Rebecca A. Siwicki, Vincent Hahaut, Yanyan Hou, Tiago M. Rodrigues, Simone Picelli, Marco Cattaneo, Josephine Jüttner, Cameron S. Cowan, Myriam Duckely, Daniel K. Baeschlin, Magdalena Renner, Vincent Unterreiner, Botond Roska

**Affiliations:** 1 Institute of Molecular and Clinical Ophthalmology Basel, 4031 Basel, Switzerland; 2 Department of Ophthalmology, University of Basel, 4031 Basel, Switzerland; 3 Novartis Biomedical Research, 4056 Basel, Switzerland; 4 Biozentrum, University of Basel, 4056 Basel, Switzerland; 5 Department of Clinical Research, University of Basel, 4031 Basel, Switzerland

**Keywords:** human organoid, compound screen, retinal organoid, photoreceptor, rod, cone, cone degeneration, rod degeneration, retinitis pigmentosa, macular degeneration, selective vulnerability, CK1, CSNK1G1, MAPK11, CK1 inhibition, MAPK11 inhibition, rd10 mice

## Abstract

Human organoids that mirror their corresponding organs in cell-type diversity present an opportunity to perform large-scale screens for compounds that protect disease-affected or damaged healthy cell types. Here, we generated 20,000 human retinal organoids with green fluorescent protein (GFP)-labeled cone photoreceptors. Since degeneration of cones is a leading cause of blindness, we induced cone death and screened 2,707 compounds with known targets for those that saved cones or those that further damaged cones. We identified inhibitors of casein kinase 1 (CK1) that protected cones, heat shock protein 90 (HSP90) inhibitors that saved cones in the short term but damaged them in the longer term, and broad histone deacetylase (HDAC) inhibition by many compounds that significantly damaged cones. Finally, we confirmed the protective effects of identified compounds in a mouse model of photoreceptor degeneration. This work provides a database for cone-damaging compounds and describes compounds and targets that can be starting points to develop neuroprotection for cones in diseases such as macular degeneration.

## Highlights

• A compound screen was performed in 20,000 human retinal organoids
• Broad HDAC I/II inhibition damages organoid cone photoreceptors
• CK1 inhibition and knockdown protect organoid cone photoreceptors from death
• CK1 inhibition protects photoreceptors in a mouse model of retinitis pigmentosa

## Graphical abstract

**Figure:**
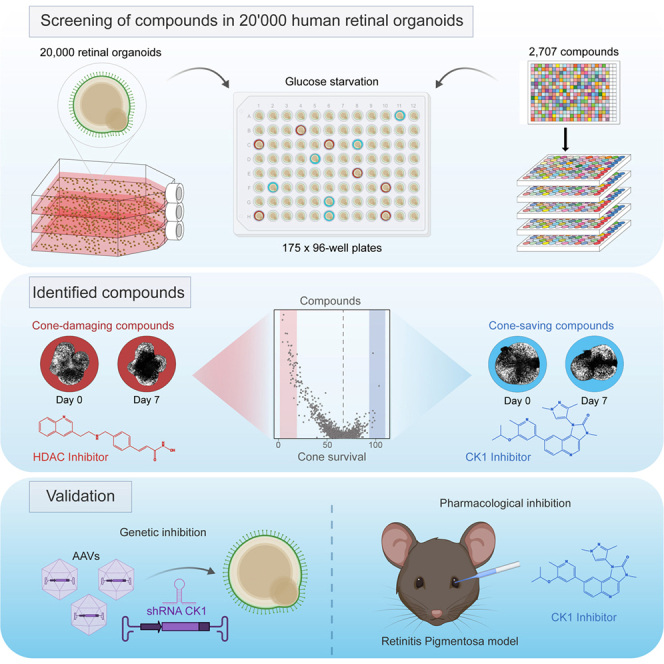

## Teaser

Spirig, Herrero-Navarro, et al. performed a compound screen in 20,000 human retinal organoids to identify cone photoreceptor damaging and protecting pathways. They identify cone toxicity from HDAC I/II inhibition and cone protection via CK1 inhibition or knockdown. They also demonstrate photoreceptor preservation in a mouse model of retinitis pigmentosa.

## Introduction

Human organoids are three-dimensional (3D) cellular assemblies grown from stem cells,^1^^,^^2^ which can be used to unravel the fundamental biology of organ development and decipher the mechanisms of genetic diseases.^2^

Two other potential uses of human organoids are to screen for compounds that can improve a disease phenotype^3^^,^^4^ and to screen for compounds with potential side effects on specific organs.^5^ These objectives rely on efficient and large-scale production of human organoids, as well as the comprehensive recording and analysis of phenotypic changes within 3D tissues. Progress has been made in screening for compounds using mouse intestinal organoids^6^ and dissociated cells from mouse retinal organoids,^7^ contributing to our understanding of organ biology and allowing for potential therapy development. However, therapies developed in mice do not always translate to humans.^8^^,^^9^^,^^10^ Screening of compounds in human organoids has only been done on a small scale, using fewer than sixty compounds, and has been limited to cancer organoids.^3^^,^^4^

The brain, including the retina, is composed of numerous cell types,^11^^,^^12^^,^^13^ and a fundamental characteristic of brain diseases is “selective vulnerability,” where the pathology predominantly affects specific cell types.^14^ Given this selective vulnerability, compound screening to find drugs that mitigate disease phenotypes in brain or retinal organoids will be most effective when it focuses specifically on the cell types affected by the disease.

The human retina is part of the brain^15^ and contains diverse cell types arranged in five layers.^16^^,^^17^^,^^18^ Photoreceptors sense light and transmit visual information to bipolar cells, which further transmit information to ganglion cells, the output neurons of the retina. Communication from photoreceptors to bipolar cells is influenced by horizontal cells, while communication from bipolar cells to ganglion cells is modulated by amacrine cell types.^15^

Photoreceptors in the retina are of two types: rods and cones. Rods are utilized in low-light conditions, and a lack of rod function results only in mild or no vision impairment.^19^ Cones are primarily responsible for image formation in daylight and enable high-resolution vision. The loss of cones or their functionality, mainly due to age-related macular degeneration or end-stage retinitis pigmentosa, results in blindness and affects over 200 million individuals worldwide.^20^^,^^21^ In age-related macular degeneration, the dysfunction or death of cones is either a direct consequence of the disease or secondary to the dysfunction of the retinal pigment epithelium.^22^ Retinitis pigmentosa, a group of monogenic retinal diseases, primarily affects rods^23^^,^^24^ and cones degenerate as a secondary consequence of rod death.^25^ The reasons behind cone degeneration in age-related macular degeneration and retinitis pigmentosa are extensively studied but are not fully understood, and several approaches are being developed to halt the degeneration process.^26^^,^^27^^,^^28^^,^^29^^,^^30^^,^^31^^,^^32^^,^^33^^,^^34^^,^^35^^,^^36^^,^^37^^,^^38^^,^^39^^,^^40^^,^^41^^,^^42^^,^^43^ A common theme for retinitis pigmentosa has emerged: cones are likely starving from a lack of glucose.^26^^,^^32^^,^^44^^,^^45^^,^^46^ Slowing down cone degeneration in patients has so far not been achieved. Given the critical importance of cones for human vision, preserving their viability remains a significant objective in medicine.

Retinal organoids were among the earliest organoids to be established.^47^^,^^48^ Since then, the technology for developing human retinal organoids has advanced rapidly and allows for the generation of complex, five-layered organoids that are light sensitive and consist of multiple cell types resembling those found in the adult human retina.^16^^,^^49^^,^^50^ Cones in human retinal organoids exhibit a close similarity to their counterparts in the adult human retina in terms of gene expression, morphology, and function^16^^,^^50^ and, thus, present a unique opportunity to study cone degeneration.

Here, we aimed to find compounds that either slow down or induce the death of cones. We produced ∼20,000 human retinal organoids and targeted green fluorescent protein (GFP) expression specifically to cones using adeno-associated viral vectors (AAVs) carrying a cone-specific promoter.^10^ We induced cone death by glucose starvation and conducted 3D imaging of the organoids before and after the starvation process, at a time when approximately 40% of cones were lost. We then evaluated a library of 2,707 compounds with known targets.^51^ This screening identified compounds that exacerbated cone death as well as compounds that counteracted the degeneration induced by glucose starvation. Broad inhibition of class I or II histone deacetylases (HDACs I/II) by various compounds resulted in significant damage to cone photoreceptors. Heat shock protein 90 (HSP90) inhibition countered cone degeneration for a few days but proved detrimental after a week. We also discovered two kinase inhibitors (cone-saving kinase inhibitor 1 [CS-KI-1] and cone-saving kinase inhibitor 2 [CS-KI-2]) that consistently preserved cones over a prolonged period. Both compounds also preserved rods. Through kinase profiling of both compounds and their inactive chemical analogs, we identified two of their potential targets: gamma 1 form of casein kinase 1 (CK1) (CSNK1G1) for CS-KI-1 and mitogen-activated protein kinase 11 (MAPK11) for CS-KI-2. Inhibiting CK1 (CSNK1G is a member of the CK1 family) with three different compounds resulted in increased survival of both cones and rods. Cone-specific downregulation of different members of the CK1 family using short hairpin RNAs (shRNAs) also protected cones from degeneration. We then developed a model of oxidative stress-induced cone degeneration in organoids and confirmed that some of the cone-saving compounds also protected cones in this model of cone degeneration. Finally, injections of CS-KI-1, CS-KI-2, or a CK1 inhibitor in a mouse model of retinitis pigmentosa confirmed their beneficial effects in protecting photoreceptors *in vivo*. Taken together, we describe here a technology to perform cell-type-focused screening in human organoids and a publicly available resource of 2,707 compounds and their targets, together with their positive or negative effects on human cone survival (https://ConeTargetedCompoundScreen.iob.ch). Furthermore, we identify kinase inhibitors and targets that could be starting points to develop medicine that counters cone and/or rod degeneration.

## Results

### Specific and rapid live labeling of cones

**Figure 1:**
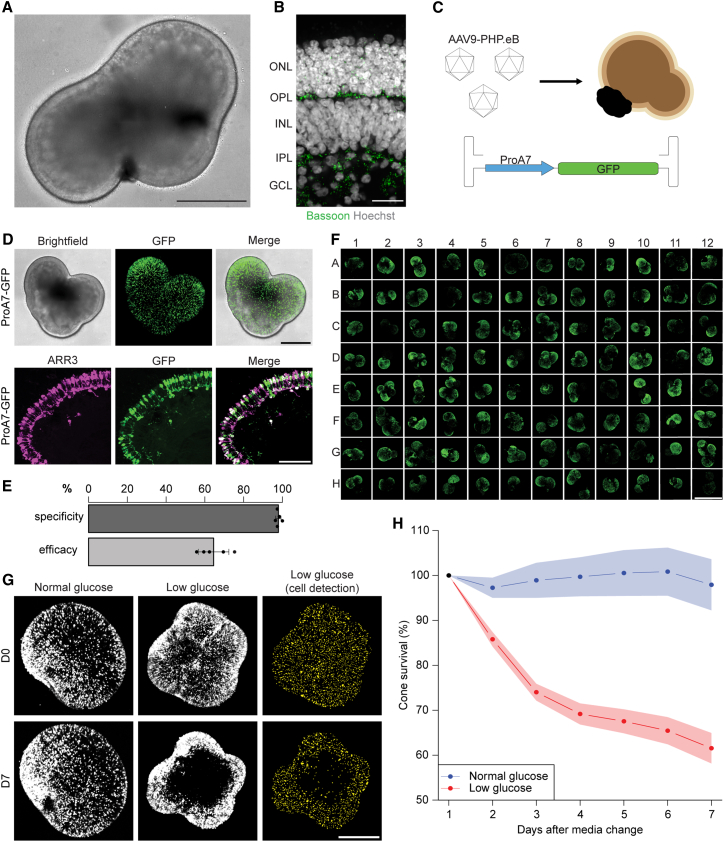
Glucose starvation induces rapid death of cones in human retinal organoids (A) Brightfield live image of a human retinal organoid (scale bar, 500 μm). (B) Confocal image of a sectioned and stained human retinal organoid (scale bar, 25 μm). Bassoon, green; Hoechst, white. ONL, outer nuclear layer; OPL, outer plexiform layer; INL, inner nuclear layer; IPL, inner plexiform layer; GCL, ganglion cell layer. (C) Schematic illustrating the transduction strategy for organoids. (D) Top: live images of a ProA7-GFP AAV-transduced human retinal organoid (scale bar, 500 μm). Bottom: confocal image of a sectioned and stained ProA7-GFP AAV-transduced human retinal organoid (scale bar, 100 μm). ARR3, magenta; GFP, green. (E) Quantification of the specificity and efficacy of cone labeling by ProA7-GFP AAV. Results are shown as mean ± SD. (F) Representative image of a 96-well plate containing ProA7-GFP AAV-transduced human retinal organoids (scale bar, 2 mm). (G) Left: representative live images of human retinal organoids at day 0 (D0) and day 7 (D7) in either normal or low-glucose medium. Right: detected cones in organoids in low glucose. GFP, white. Detected cells, yellow. (H) Quantification of cone survival in human retinal organoids in both normal- and low-glucose conditions over 7 days. The quantification was performed using the 3D-additive-count algorithm. The results are shown as mean ± SE. See also Figure S1.

We modified the agarose multiwell array seeding and scraping (AMASS) method^16^ to generate ∼20,000 five-layered human retinal organoids that were grown for 30 weeks (Figures 1A and 1B). These organoids contain the major cell classes and various cell types found in the human retina^16^ (Figure S1).

To visualize living cones, we transduced organoids with AAVs expressing GFP under control of the cone-specific promoter ProA7^10^ (Figures 1C and 1D). We tested five different capsid variants and found that AAVs with the AAV9-PHP.eB capsid^52^ provided the highest number of labeled cones within four weeks. The cone labeling specificity in organoids was 97% ± 1% (*n =* 5), while the efficacy was 64% ± 8% (*n =* 5) (Figure 1E). We performed AAV transduction in cell culture flasks that allowed simultaneous labeling of ∼130 organoids, which were subsequently positioned into 96-well plates for 3D imaging (Figure 1F). As a result, we achieved GFP labeling of cones in a large number of organoids with high specificity, efficacy, and uniformity.

### Glucose starvation induces cone death

To induce cone death, we cultured organoids individually in 96-well plates using low-glucose medium.^53^ We recorded cell death by monitoring the disappearance of cytosolic GFP^54^^,^^55^^,^^56^ using 3D confocal imaging in each well of the 96-well plate. The organoids were imaged for two weeks without a change of the medium to avoid any displacement of the organoids. Additionally, we included organoids in normal-glucose medium in the same 96-well plate as controls (Figure 1G).

We developed three different cell-counting algorithms to determine the number of cones (Figure 1G). The first (referred to as “3D-additive-count”) quantifies cones image-by-image from a 3D confocal image stack. The second (“3D-count”) counts cones from the entire 3D stack. The third (“MIP-count”) counts cones from the maximum intensity projection of the 3D stack. The primary measure of cone numbers was based on the 3D-additive-count, and we verified the results using the 3D-count and MIP-count methods.

We measured glucose concentration in the low- and normal-glucose media during organoid imaging. Initially, glucose levels in the low-glucose medium were 9% of those in the normal-glucose medium. With time, glucose levels decreased in both the low- and the normal-glucose media, becoming undetectable after 2 days in the low-glucose medium and 7 days in the normal-glucose medium (Figure S1).

Next, we examined the number of cones over the two-week period. In low-glucose medium, organoids began losing cones after 2 days of starvation, and cone loss continued over the two weeks. Conversely, cone numbers in control organoids in normal-glucose medium remained constant until day 7, after which they declined (Figures 1G, 1H, and S1). Therefore, we conducted further experiments for 7 days. Within this time frame, glucose-deprived organoids lost ∼40% of their cones, leading to a significant difference in cone numbers in low- and normal-glucose conditions (low glucose, *n =* 26; normal glucose, *n =* 6, *p <* 0.001, Mann-Whitney U test) (Figure 1H). Cone cell death occurred predominantly in the central region of organoids rather than at the edges (Figure 1G). This spatial variation is likely attributable to the additional stress experienced by cells in contact with the bottom of the well. The finding that about half of the cones were lost by the end of 7 days in low-glucose medium enabled us to screen for compounds that either slow down cone death or accelerate it.

We then performed two compound screens. In the primary screen, we tested all compounds at 10 μM. In the secondary screen, we selected compounds based on the results of the primary screen and retested them at multiple concentrations.

### Primary compound screen

**Figure 2:**
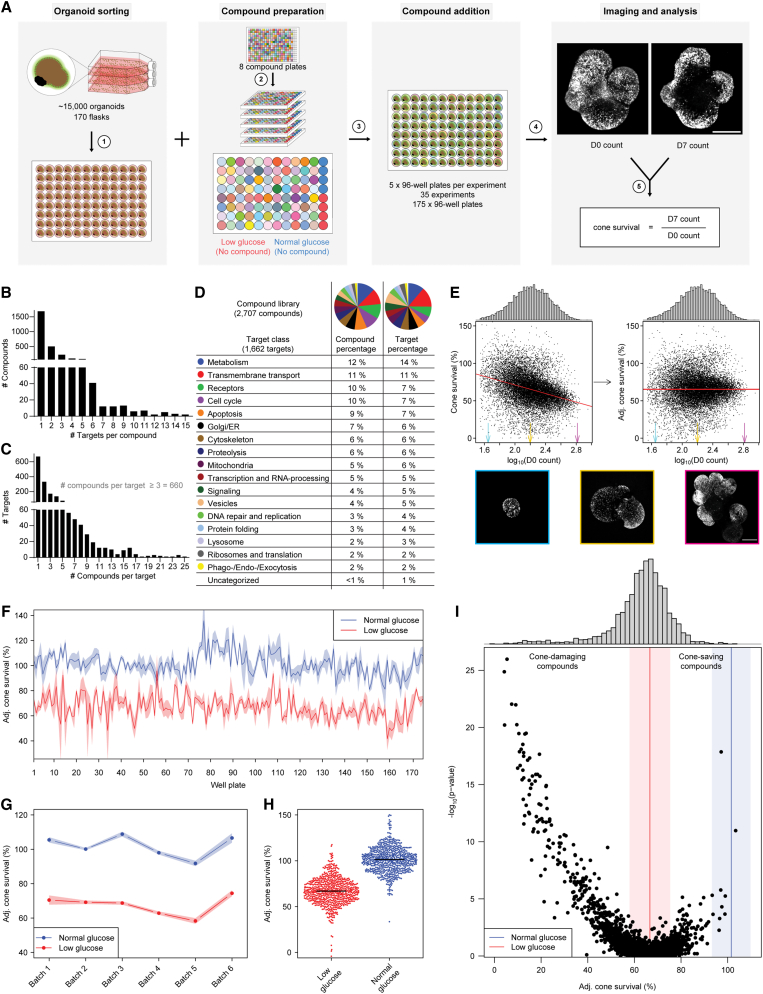
Primary screen of compounds that damage or save cones (A) Schematic illustration of the primary screen. (1) Transfer of human retinal organoids from cell culture flasks to 96-well plates. (2) Compound distribution from 384- to 96-well plates with five replicates of each compound. Low-glucose controls, red. Normal-glucose controls, blue. (3) Addition of compounds to human retinal organoids. (4) Human retinal organoid imaging. (5) Quantification of cones from the same human retinal organoid at day 0 (D0) and day 7 (D7). GFP, white. (B) Bar chart showing the number of compounds with a given number of targets. (C) Bar chart showing the number of targets with a given number of compounds per target. (D) Categorization of targets and compounds of the compound library. (E) Dependence of cone survival on the day 0 cone count. Top left: cone survival as a function of the logarithm of the cone count at day 0 (D0), with a fitted linear model, and the regression line is shown in red. The distribution of day 0 counts is shown above. Top right: adjusted cone survival as a function of the logarithm of the cone count at day 0 with the transformed regression line in red. The distribution of day 0 counts is shown above. Bottom: example images with different day 0 cone counts (scale bar, 500 μm). Colored arrows and frames around images indicate corresponding day 0 cone counts. GFP, white. (F and G) Adjusted cone survival of normal- and low-glucose controls for individual well plates (F) and organoid batches (G) using the 3D-additive-count algorithm, mean ± SE. (H) Adjusted cone survival of all normal- and low-glucose control human retinal organoids. (I) Effect of compounds on cone survival in the primary screen. Each dot corresponds to the effect of one compound, with the median of the adjusted cone survival of five human retinal organoids on the *x* axis and the *p* value comparing the cone survival between the compound and the low-glucose controls on the *y* axis. The median (line) and the interquartile range (shaded area) of normal (blue) and low (red) glucose controls are indicated. Top: the distribution of median adjusted cone survival for compounds. Results were obtained using the 3D-additive-count algorithm. See also Figures S2 and S3 and Table S1.

In the primary screen, we used ∼15,000 organoids distributed across 175 separate 96-well plates. Each 96-well plate included eight control wells, consistently positioned. Four of these control wells contained organoids in normal-glucose medium with dimethyl sulfoxide (DMSO, 0.1%) but no compounds. The other four contained organoids in low-glucose medium (DMSO, 0.1%) without compounds. Each remaining well (88 wells) within a 96-well plate contained a unique compound, and each compound was present in five different plates, resulting in five replicates of each compound (Figure 2A). All compounds were stored in DMSO. Compounds were stored in 384-well plates, and we conducted the screening in 96-well plates because organoids were too large to be cultured in 384-well plates.

In preparation for the screen, we transferred compounds from a 384-well plate to their designated locations on 96-well plates and then dissolved them in low-glucose medium. To initiate the screen, we first moved organoids from flasks containing normal-glucose medium to 96-well plates and washed them there in low-glucose medium. We then removed the low-glucose medium from the 96-well plates containing the organoids and added the compounds dissolved in low-glucose medium. We conducted a 3D confocal scan of organoids with GFP-labeled cones in each well of the 96-well plates at the beginning of the screen and again at the end, 7 days later. We then used the three algorithms to quantify the number of cones at days 0 (D0) and 7 (D7), defining the ratio of counts at day 7 and day 0 as the measure of cone survival (Figure 2A).

We screened a compound library of 2,707 compounds. The compound library, called the “mode of action library” (MOA library), contained compounds with known protein targets.^51^ Some compounds had multiple targets, while others had only one (Figure 2B). Similarly, certain targets were affected by multiple compounds, while others were affected by only one compound (Figure 2C). The MOA library had a total of 1,662 targets that are involved in different biological processes (Figure 2D).

To exclude accidentally empty wells or organoids with little or no labeled cones, we applied a threshold on the day 0 cone count and removed wells with lower day 0 counts from the dataset (Figure S2). Furthermore, we observed that the survival of cones was determined not only by the compounds but also by the initial cone count. There was a log-linear relationship between the day 0 count and cone survival at day 7 in low glucose (*n =* 14,529, R^2^ = 0.12, *p <* 0.001, 3D-additive-count) with all three cell-counting algorithms (Figures 2E and S2). We therefore adjusted the values of cone survival using a linear transformation, yielding the quantity “adjusted cone survival.” Adjusted cone survival therefore does not depend on the cone count at day 0.

The locations of organoids on the 96-well plates did not influence cone survival: the mean adjusted survival at all positions (8.8% maximum difference between positions) remained within one standard deviation of that of the position exhibiting the least variation (12.9%), excluding the normal-glucose well positions (Figure S2).

To investigate additional potential batch effects in the screen, we examined the distribution of the mean adjusted cone survival of the eight control organoids—four in normal and four in low glucose—across the 175 separate 96-well plates (Figures 2F and S2), across the 35 experiments, each consisting of five 96-well plates with the same compound (Figure S2), and across the six independent organoid productions used for the primary screen (Figures 2G and S2). We found no major differences between batches at any level. The mean adjusted cone survival of normal- and low-glucose control organoids was significantly different across all 35 experiments and across all six organoid productions, using all three cell-counting algorithms (*p* values < 0.001; Mann-Whitney U test) (Figures 2G, 2H, and S2). Cone survival values showed strong and significant correlations across the three cell-counting algorithms (R = 0.92–0.97, *p* values < 0.001, Pearson correlation) (Figure S3), suggesting that the quantification of cone survival is robust across the different algorithms.

We then analyzed the outcome of the primary screen. The mean adjusted cone survival of most compounds was not significantly different from the mean adjusted cone survival of the low-glucose control. However, one set of compounds significantly impacted cone survival negatively (“cone-damaging compounds,” *p* < 0.05, analysis of covariance [ANCOVA] with Benjamini-Hochberg correction for multiple testing), while another set of compounds showed a beneficial effect on cone survival (“cone-saving compounds”) (Figures 2I and S3).

### Secondary screen: Cone-damaging compounds

In order to validate the cone-damaging and cone-saving compounds detected in the primary screen and to assess the concentration dependence of their actions, we proceeded to secondary screens.

**Figure 3:**
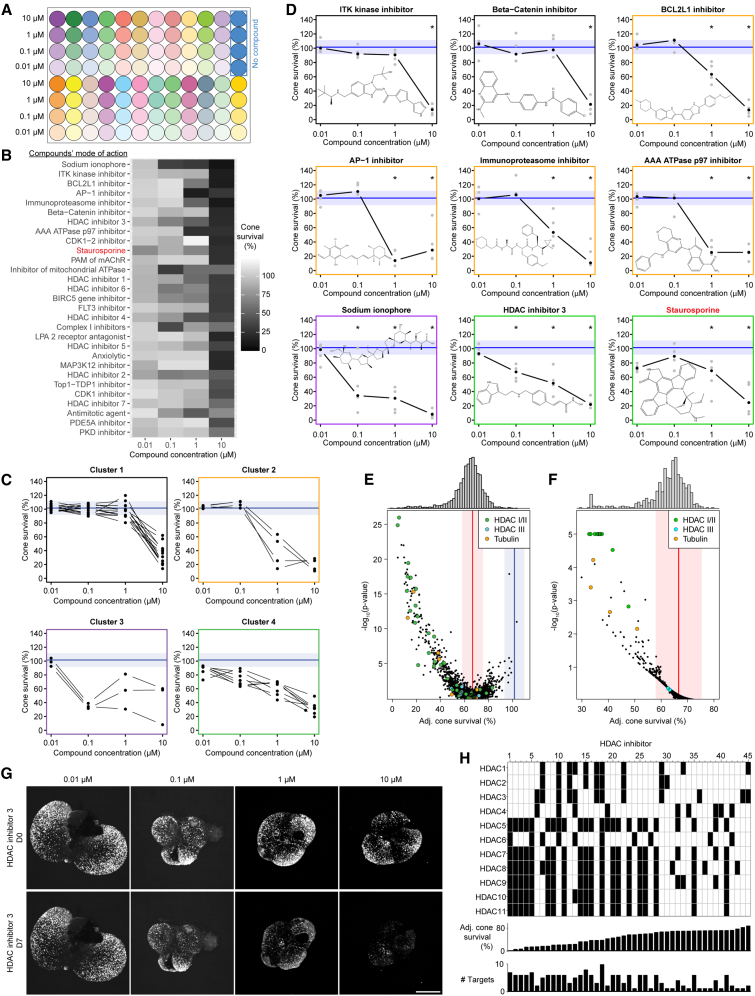
Secondary screen of cone-damaging compounds (A) Schematic illustration of the plate layout in the secondary screen for cone-damaging compounds. Different colors represent distinct compounds. No compound control, blue. (B) Summary of compound effects for significant cone-damaging compounds (*p <* 0.05 for at least one concentration after Benjamini-Hochberg correction for multiple testing). The positive control staurosporine is indicated in red. Compounds are ordered by minimum *p* value per concentration, with the smallest *p* value at the top. (C) Dose-responses for significant compounds within the same cluster. Dots represent the median of cone survival at the indicated concentrations. The median (line) and the interquartile range (shaded area) of normal (blue) glucose controls are indicated. (D) Dose-response curves of eight compounds with the lowest *p* values (at any concentration) and staurosporine (red) with their corresponding cluster indicated. The median and the interquartile range are labeled as in (C). The colored frame indicates the corresponding cluster from (C). The structure of each compound is shown in the plot. Significant compounds and concentrations are marked with ^∗^, denoting a *p* value < 0.05 (after Benjamini-Hochberg correction for multiple testing). (E) Adjusted cone survival and *p* values in the primary screen. HDAC I/II (green), HDAC III (cyan), and tubulin (orange) inhibitors are labeled. The median (line) and the interquartile range (shaded area) of normal (blue) and low (red) glucose controls are indicated. (F) Target analysis of primary screen. The means of the median adjusted cone survival for each target are shown, along with their *p* values. Compound targets are labeled. HDAC I/II, green; HDAC III, cyan; tubulin, orange. The median (line) and the interquartile range (shaded area) of low (red) glucose controls are indicated. (G) Example images of human retinal organoids before (day 0 [D0]) and after (day 7 [D7]) treatment with different concentrations of HDAC inhibitor 3 (scale bar, 500 μm). GFP, white. (H) Top: different HDAC I/II inhibitors (numbered from 1 to 45) with their targets (black shaded areas). Compounds are sorted from left to right based on median adjusted cone survival. Middle: adjusted cone survival for each of the 45 compounds. Bottom: number of HDAC I/II targets of the 45 compounds. See also Figures S4 and S5 and Tables S1 and S2.

First, we revisited the 33 most damaging compounds selected from a set of 146 compounds that caused significant damage beyond that induced by the low-glucose medium in the primary screen (*n =* 5 for each compound, *p* < 0.05, ANCOVA with Benjamini-Hochberg correction for multiple testing) (Table S1). We investigated these compounds at four concentrations (0.01, 0.1, 1, and 10 μM) in five replicates using normal-glucose medium. We used normal-glucose medium to confirm the damaging effects of these compounds on cones of healthy organoids. Furthermore, each 96-well plate included four wells with only DMSO in normal-glucose medium, serving as a negative control (Figure 3A). As for the positive control, we utilized staurosporine, a nonselective ATP-competitive kinase inhibitor known for inducing apoptosis.^57^^,^^58^ This was also tested in five replicates at each of the four concentrations.

Most compounds (29 out of 33) again induced a significant decrease in the number of cones compared with the negative control (*n =* 5 for each compound and concentration, minimum *p* value < 0.05, ANOVA with Benjamini-Hochberg correction for multiple testing) (Figure 3B). Nine caused a more significant decrease in cone numbers than the positive control, staurosporine, which reduced cone numbers by 76%. The remaining compounds reduced cone numbers by at least 38% (Figures 3B, S4, and S5; Table S2).

We observed a variety of dose-response curves that could be clustered into four groups. The first cluster included compounds that led to cone death only at the highest concentration (10 μM), such as an ITK kinase inhibitor and a beta-catenin inhibitor. The second cluster of curves had an action threshold of 1 μM and included a BCL2L1 inhibitor, an AP-1 inhibitor, an immunoproteasome inhibitor, and an AAA ATPase p97 inhibitor. The third cluster of curves had an action threshold of 0.1 μM. For example, a sodium ionophore had such a curve. For the fourth cluster of curves, cone death increased linearly with the logarithm of compound concentration. Most of these curves belonged to HDAC I/II inhibitors (Figures 3C, 3D, and S5).

Of the 146 compounds that caused significant damage to cones in the primary screen (*p* < 0.05, ANCOVA with Benjamini-Hochberg correction for multiple testing), 19 were HDAC I/II inhibitors (Figure 3E; Table S1). In the secondary screen, seven of the 28 compounds that significantly damaged cones were HDAC I/II inhibitors (Figures 3B and S4). To determine whether this high number of HDAC I/II inhibitors is a result of bias in the MOA library toward HDAC I/II inhibitors, we reanalyzed the results of the primary screen. We calculated the probability that the mean adjusted cone survival for a randomly selected set of 45 compounds is lower than the mean adjusted cone survival for the 45 different HDAC I/II inhibitors in the MOA library. This probability was smaller than 0.0001, implying that, on average, the 45 HDAC I/II inhibitors in the MOA library cause more damage to cones than a random selection of the same number of other compounds.

To systematically investigate whether modulating any specific target is significantly more harmful to cones than modulating other targets, we first selected targets that had a minimum of three compounds listed in the MOA library. We identified a total of 660 such targets (Figure 2C). Next, we calculated the probability of observing a lower mean adjusted cone survival when randomly selecting the same number of compounds in comparison to the compounds associated with the target. We identified 10 targets with a *p* value of less than 0.0001 and less than 0.05 after Benjamini-Hochberg correction for multiple testing. These targets belonged exclusively to class I/II HDACs (Figure 3F). Furthermore, when evaluating the percentage of compounds that led to significant cone damage among all compounds targeting each specific target, HDACs had the highest values (Figure S4). Each HDAC I/II target was associated with 9–29 distinct compounds, out of which 44%–67% induced significant damage to cones. Interestingly, compounds that acted on sirtuins, which are class III HDACs, had no influence on cone survival (Figures 3E, 3F, and S4). HDAC I/II inhibitors often affect multiple HDAC I/II targets, and we found a significant negative correlation between the number of HDAC I/II targets of a specific inhibitor and adjusted cone survival (*p <* 0.001, R = −0.6, Spearman’s correlation). Therefore, cones are specifically sensitive to HDAC I/II inhibition, and HDAC I/II inhibitors with a wide range of targets are more likely to result in cone damage than those that are more selective (Figures 3G and 3H).

Cones were also highly sensitive to the inhibition of tubulins (Figures 3E, 3F, and S4). The probability that the mean adjusted cone survival for a random set of 10 compounds was lower than that of the 10 distinct tubulin inhibitors in the MOA library was 0.002. Of the 10 tubulin-targeting compounds, four resulted in a significant decrease in cone numbers. Notably, three of these compounds had a broad target range, affecting 19–20 different tubulins.

### Secondary screen: Cone-saving compounds

**Figure 4:**
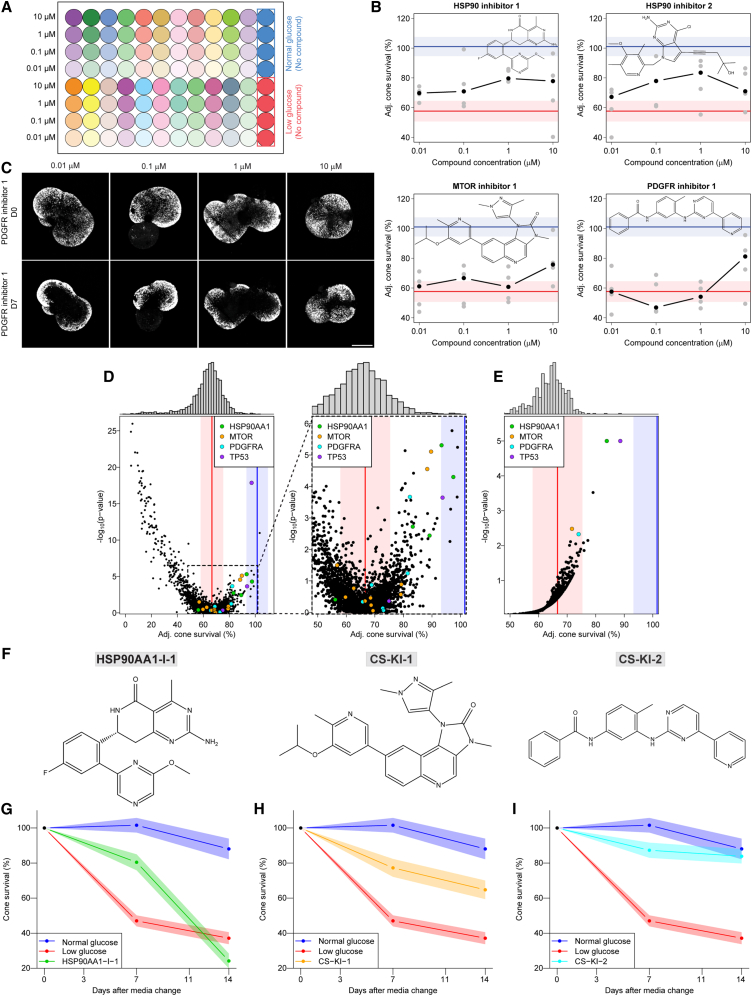
Secondary screen of cone-saving compounds (A) Schematic illustration of plate layout in the secondary screen for cone-saving compounds. Different colors indicate distinct compounds. Low-glucose controls, red. Normal-glucose controls, blue. (B) Dose-response curves of the four significant compounds. Black dots, median adjusted cone survival; gray dots, individual adjusted cone survival values. The median (line) and the interquartile range (shaded area) of normal (blue) and low (red) glucose controls are indicated. The structure of each compound is shown in the plot, and the annotated target is indicated on top. (C) Example images of human retinal organoids at day 0 (D0) and day 7 (D7) with different concentrations of PDGFR inhibitor 1 (scale bar, 500 μm). GFP, white. (D) Left: adjusted cone survival and *p* values in the primary screen. Compound targets are labeled. HSP90AA1, green; MTOR, orange; PDGFRA, cyan; TP53, purple. The median and the interquartile range are labeled as in (B). Right: zoom-in of plot on the left. (E) Target analysis of the primary screen. The means of the median adjusted cone survivals for each target are shown, along with their *p* values. HSP90AA1, green; MTOR, orange; PDGFRA, cyan; TP53, purple. The median and the interquartile range are labeled as in (B). (F) Chemical structures and new names of the indicated compounds. (G–I) Time course of cone survival in glucose-starved human retinal organoids with the indicated compounds for 14 days. Results are shown as mean ± SE. See also Figures S6–S9 and Table S3.

Seven compounds in the primary screen significantly increased cone survival. We proceeded with these compounds as well as 24 other compounds that had the highest effect in protecting cones in the primary screen. These 31 compounds were tested at four different concentrations (0.01, 0.1, 1, and 10 μM) in five replicates in low-glucose medium. Each 96-well plate included four wells containing only DMSO in normal-glucose medium as a positive control and four wells containing only DMSO in low-glucose medium as a negative control (Figure 4A).

We confirmed that four out of the 31 compounds tested in the secondary screen have a significant positive impact on cone survival after correction for multiple testing (*n =* 5 for each compound and concentration, minimum *p* values < 0.05, ANCOVA with Benjamini-Hochberg correction for multiple testing) (Figures 4B, 4C, S6, and S7; Table S3). These four compounds include an inhibitor of HSP90AA1 (“HSP90 inhibitor 1”), an inhibitor of both HSP90AA1 and HSP90AB1 (“HSP90 inhibitor 2”), an inhibitor of MTOR, PIK3CA, PIK3CB, and PIK3CD (“MTOR inhibitor 1”), and an inhibitor of PDGFRA and PDGFRB (“PDGFR inhibitor 1”). The two HSP90 inhibitors demonstrated stronger activity at lower concentrations (0.1 or 1 μM) and less activity at a concentration of 10 μM (Figure 4B). The dose-response curves of the MTOR inhibitor and the PDGFR inhibitor showed similar patterns, being effective only at the highest concentration of 10 μM (Figures 4B and 4C).

To understand the relationship between the four compounds and their targets in the context of cone protection, we reanalyzed the results of the primary screen, focusing on the targets of the four inhibitors that were confirmed to protect cones. We found five compounds in the MOA library that target HSP90AA1, four of which resulted in adjusted cone survival higher than 80% (although some of these were not significant in the primary screen). For all other targets, the percentages of compounds with a positive effect on cone survival were only between 11% and 33% (Figures 4D and S8). These results suggest that HSP90AA1 is the target of HSP90 inhibitors 1 and 2 in cones. Additionally, they suggest that the other two compounds (MTOR inhibitor 1 and PDGFR inhibitor 1), which are both kinase inhibitors, have different causal targets in human retinal organoids than those originally listed in the MOA library.

To systematically examine whether modulating particular targets has a greater positive impact on cones compared with other targets, we conducted an analysis focusing on 660 targets that had at least three partner compounds listed in the MOA library. To rank targets in their ability to protect cones, we calculated the probability of observing a higher mean adjusted cone survival in the primary screen when randomly selecting the same number of compounds in comparison to the compounds associated with the target. HSP90AA1 emerged as one of the top-ranked targets (*p <* 0.00001 and *p <* 0.01 after Benjamini-Hochberg correction for multiple testing). Targets such as HSP90AB1, MTOR, PIK3CA, PIK3CB, PIK3CD, PDGFRA, and PDGFRB were not significant because a large proportion of compounds acting on these targets had no impact on cone survival (Figures 4D, 4E, and S8). TP53 was a further significant target (*p <* 0.00001 and *p <* 0.01 after Benjamini-Hochberg correction for multiple testing), for which there were three compounds in the library: two inhibitors and one expression enhancer. However, both inhibitors and the expression enhancer increased cone survival in the primary screen, and the enhancer had no significant effect after retesting, suggesting that TP53 is not a target for cone protection.

To further explore the connection between the four validated compounds and their targets, we compared available data on IC50 (the concentration at which a compound shows 50% of its maximum inhibitory effect) with the effect on cone survival of inhibitors of HSP90AA1, HSP90AB1, MTOR, PIK3CA, PIK3CB, PIK3CD, PDGFRA, and PDGFRB. We found a significant negative correlation between the reported IC50s and the adjusted cone survival for the inhibitors of HSP90AA1 (R = −0.7, Spearman’s correlation, *p* = 0.03) (Figure S8). This suggests that compounds with lower IC50 values (indicating greater potency) are linked to higher adjusted cone survival. However, the IC50s for the inhibitors of HSP90AB1, MTOR, PIK3CA, PIK3CB, PIK3CD, PDGFRA, and PDGFRB were not significantly correlated with cone survival. These findings further confirm that HSP90AA1 is a target that, when inhibited, effectively protects cones from the effects of glucose starvation at day 7. Therefore, we have renamed HSP90 inhibitor 1 as “HSP90AA1-I-1” and HSP90 inhibitor 2 as “HSP90AA1-I-2.” On the other hand, we could not confirm that HSP90AB1, MTOR, PIK3CA, PIK3CB, PIK3CD, PDGFRA, and PDGFRB are causal targets that affect organoid cone survival. Since MTOR inhibitor 1 and PDGFR inhibitor 1 are kinase inhibitors, we renamed them as CS-KI-1 and CS-KI-2, respectively (Figure 4F).

We conducted additional tests on the three compounds HSP90AA1-I-1, CS-KI-1, and CS-KI-2 over a 14-day period. We administered these compounds in the concentrations that had the strongest effect during the secondary screen: 1 μM for HSP90AA1-I-1 and 10 μM for CS-KI-1 and CS-KI-2 (Figure 4B). We treated organoids with these compounds in low-glucose medium and performed imaging after 7 and 14 days, using normal- and low-glucose media with DMSO (without the compounds) as negative and positive controls, respectively (Figures 4G–4I).

HSP90AA1-I-1 showed significant protection of cones at 7 days but became detrimental to cones at 14 days compared with the low-glucose control (*n =* 10, 7 days: *p <* 0.001, 14 days: *p =* 0.01, Mann-Whitney U test) (Figure 4G). By contrast, CS-KI-1 and CS-KI-2 had a significant rescuing effect on cones at both seven and 14 days (low glucose, *n =* 10; CS-KI-1, *n =* 9, 7 days: *p <* 0.001, 14 days: *p =* 0.002; CS-KI-2, *n =* 10, 7 days: *p <* 0.001, 14 days: *p <* 0.001, Mann-Whitney U test) (Figures 4H and 4I). These findings suggest that HSP90AA1 inhibition is detrimental to cone survival in the long term and that the protective effect of CS-KI-1 and CS-KI-2 on cones is of long duration.

To investigate the possibility that compound treatment led to apparent cone saving by increasing GFP expression, we tested the effects of HSP90AA1-I-1, CS-KI-1, and CS-KI-2 on organoids under normal-glucose conditions. None of these compounds led to an increase or decrease in cone survival after 7 days compared with the normal-glucose control condition (no compound). Thus, the observed rescue effect of these compounds is not derived from an artifact of increased GFP expression (Figure S9). In addition, we quantified the specificity of GFP-labeled cells in retinal organoids exposed to cone-saving compounds identified in the screen. By counting GFP+ cells that were also positive for the cone-specific marker ARR3, we found that cone specificity was similar in organoids in normal glucose and in organoids under low-glucose conditions, either untreated or treated with CS-KI-1 and CS-KI-2 (normal glucose, *n =* 4; low glucose, *n =* 4, *p =* 0.412; CS-KI-1, *n =* 4, *p >* 0.999; CS-KI-2, *n =* 4, *p >* 0.999; Kruskal-Wallis test with Dunn’s correction for multiple comparisons) (Figure S9). Therefore, counting the number of GFP+ cells reflects the proportional number of cones under all these experimental conditions (Figure S9).

### Effect of HSP90AA1-I-1, CS-KI-1, and CS-KI-2 on rod photoreceptor death

**Figure 5:**
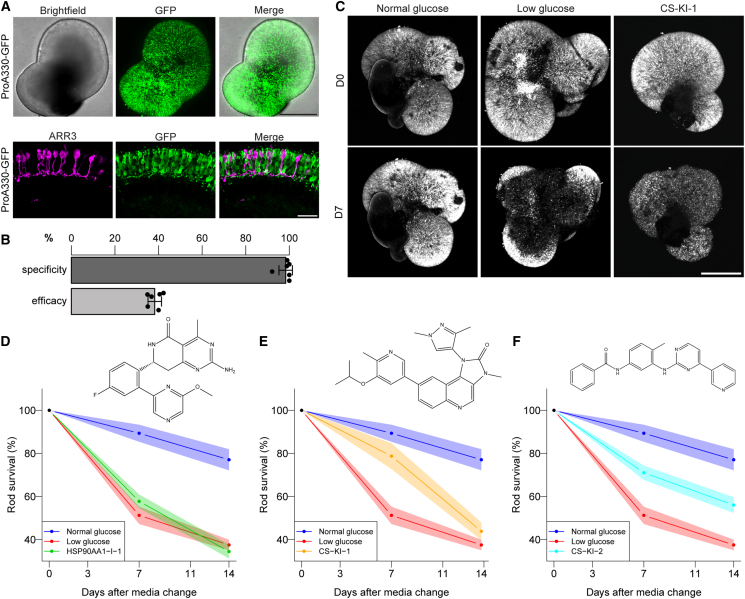
Effect of cone-saving compounds on rods (A) ProA330-GFP AAV transduction of human retinal organoids. Top: live image of a ProA330-GFP-transduced human retinal organoid (scale bar, 500 μm). Bottom: confocal image of a sectioned and stained transduced human retinal organoid (scale bar, 25 μm). Rods were identified as being present in the photoreceptor layer but negative for the cone-marker ARR3. ARR3, magenta; GFP, green. (B) Quantification of the specificity and efficacy of rod labeling by ProA330-GFP AAV. Results are shown as mean ± SD. (C) Example images of ProA330-GFP AAV-transduced human retinal organoids at day 0 (D0) and day 7 (D7) in either normal glucose, low glucose, or low glucose with CS-KI-1. GFP, white. (D–F) Time course of rod survival in glucose-starved human retinal organoids treated with the indicated compounds for 14 days. Results are shown as mean ± SE. Structures of the compounds are shown. See also Figure S10.

HSP90AA1-I-1, CS-KI-1, and CS-KI-2 each had a protective effect on cones after 7 days of glucose starvation, and we examined whether they would offer a similar protection to rods. We developed a promoter, ProA330, which targeted rods in human retinal organoids. Using AAV9-PHP.eB with ProA330 driving GFP, we observed GFP expression in rods with a specificity of 98% ± 3% (*n =* 6) and an efficacy of 38% ± 3% (*n =* 6) (Figures 5A and 5B). ProA330 also drove specific expression in rods of mouse retina *in vivo* with a specificity of 99.7% ± 0.5% and an efficacy of 41% ± 7% (*n =* 3 retinas, 2 mice) (Figure S10).

Similar to cones, rods also died during glucose starvation, with a survival by day 7 of 51% in low glucose and 89% in normal glucose. The number of remaining rods was significantly different in low- and normal glucose (low glucose, *n =* 11, normal glucose, *n =* 12, *p <* 0.001, Mann-Whitney U test) (Figures 5C and 5D). Rod survival dropped further after 14 days to 37% in low glucose and 77% in normal glucose (low glucose, *n =* 9; normal glucose, *n =* 6; *p <* 0.001, Mann-Whitney U test).

Treatment with 10 μM CS-KI-1 or 10 μM CS-KI-2 led to an increase in rod survival after 7 days of starvation (low glucose, *n =* 11; CS-KI-1, *n =* 18, *p =* 0.002; CS-KI-2, *n =* 18, *p <* 0.001, Mann-Whitney U test) (Figure 5D). However, only CS-KI-2 had a significant protective effect after 14 days (*p <* 0.001, Mann-Whitney U test). We found no significant protective or detrimental effect of HSP90AA1-I-1 on rod photoreceptors at any time.

### CS-KI-1 and CS-KI-2 nonfunctional analogs

**Figure 6:**
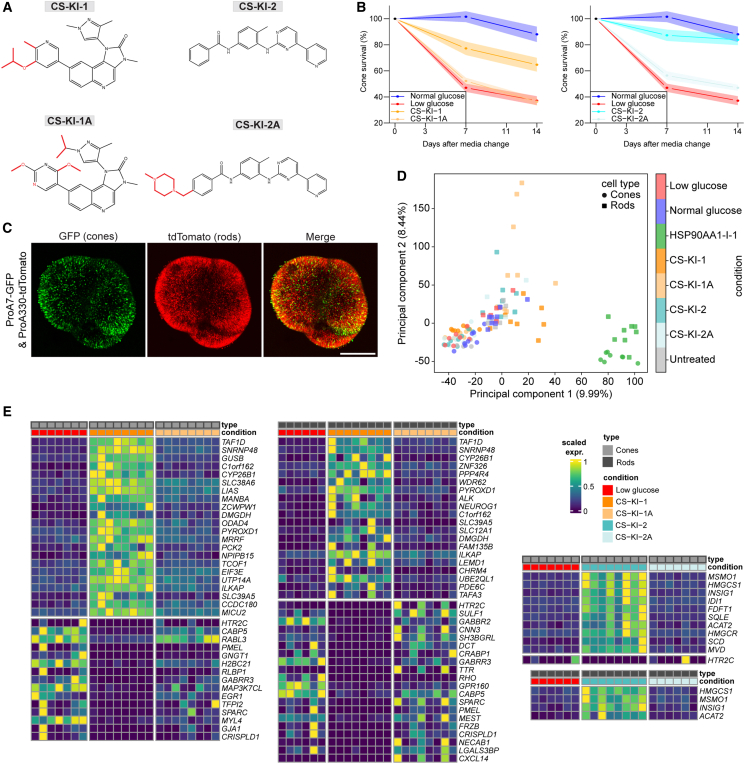
Transcriptomes of photoreceptors treated with cone-saving compounds (A) Chemical structures of CS-KI-1 and CS-KI-2 and their corresponding nonfunctional analogs. Structural differences between functional compounds and nonfunctional analogs are in red. (B) Time course of cone survival in glucose-starved human retinal organoids with the indicated compounds for 14 days. Results are shown as mean ± SE. Same data as in Figures 4H and 4I, but including the nonfunctional analog compounds CS-KI-1A and CS-KI-2A. (C) Human retinal organoids transduced with ProA7-GFP (green) and ProA330-tdTomato (red, scale bar, 500 μm). (D) Principal component analysis of transcriptomes under the indicated conditions. (E) Differential gene expression comparing human retinal organoid cone and rod transcriptomes treated with the indicated conditions. The color scale indicates row-wise normalized gene expression levels. The top 40 differentially up- or downregulated genes are shown. See also Figures S11–S16.

A problem with identifying targets of CS-KI-1 and CS-KI-2 as well as potential transcriptional changes caused by CS-KI-1 and CS-KI-2 is that these compounds are known inhibitors of MTOR and PDGFR, respectively. At the same time, they also interact with unknown pathways that protect cones. Therefore, we used the following strategy to find MTOR- and PDGFR-pathway-independent targets of CS-KI-1 and CS-KI-2 as well as to identify transcriptional changes induced by CS-KI-1 and CS-KI-2. We identified an MTOR inhibitor used in the primary screen that did not protect cones but had a very similar chemical structure to CS-KI-1. We named this CS-KI-1A (CS-KI-1 analog). Similarly, we identified a PDGFRA inhibitor that did not protect cones but had a very similar structure to CS-KI-2, which we named CS-KI-2A (Figure 6A).

We repeated experiments with 10 μM CS-KI-1, CS-KI-1A, CS-KI-2, and CS-KI-2A to assess their effectiveness in preventing cone death induced by glucose starvation. As in the primary screen, CS-KI-1A did not improve cone survival compared with low glucose, and its effect was significantly different from that of CS-KI-1 (*n =* 9, *p =* 0.002, Mann-Whitney U test) (Figure 6B). The same relation was observed between CS-KI-2A and low glucose and CS-KI-2 (*n =* 10, *p <* 0.001, Mann-Whitney U test) (Figure 6B).

We then looked for targets of CS-KI-1 and CS-KI-2 that are not targets of CS-KI-1A and CS-KI-2A, respectively, using kinase profiling. Similarly, we looked for differential changes in transcription caused by CS-KI-1 and CS-KI-1A, as well as by CS-KI-2 and CS-KI-2A.

### Transcriptomic changes in cones and rods induced by cone-saving compounds

We generated dual-color retinal organoids with green-fluorescent cones and red-fluorescent rods by transducing them with AAV9-PHP.eB capsid-coated ProA7-GFP and ProA330-tdTomato AAVs simultaneously (Figure 6C). This allowed us to isolate cones and rods from the same organoids using fluorescence-activated cell sorting (FACS) (Figure S11) and to analyze their transcriptomes using bulk RNA sequencing (RNA-seq).

We aimed to identify genes and pathways involved in the survival of cones or rods, specifically those whose expression is altered by CS-KI-1 compared with CS-KI-1A and low-glucose controls, or by CS-KI-2 compared with CS-KI-2A and low-glucose controls. In addition, we also analyzed the effect of HSP90AA1-I-1 inhibition on photoreceptor transcriptomes. We determined the transcriptomes of cones and rods in normal-glucose control organoids and in organoids exposed to 7 days of glucose starvation in the presence or absence of HSP90AA1-I-1, CS-KI-1, CS-KI-1A, CS-KI-2, or CS-KI-2A.

Cells that were GFP positive expressed marker genes for cones, while tdTomato-positive cells expressed marker genes for rods in both the normal- and the low-glucose conditions, indicating that the transcriptomic identity of cones and rods was not affected by glucose starvation (Figure S12). We asked whether glucose starvation affects S cones and L/M cones preferentially. We compared the expression levels of genes specific for S (*OPN1SW*) and L/M cones (*THRB*) in organoids exposed to normal or low glucose. We did not observe any bias, suggesting that the ratio between S and L/M cones is conserved after glucose starvation (Figure S12).

The annotated target gene of HSP90AA1-I-1, *HSP90AA1*, was highly expressed in both cones and rods. Expression of the annotated target genes of CS-KI-1, including *MTOR*, *PIK3CA*, *PIK3CB*, and *PIK3CD*, was low. Moreover, the annotated target genes of CS-KI-2, *PDGFRA*, and *PDGFRB* were not or barely expressed in cones and rods (Figure S12). These findings further support the notion that HSP90AA1 is a target of HSP90AA1-I-1 in cones, while PDGFRA and PDGFRB are not targets of CS-KI-2 in cones and rods.

The remaining glucose is low in normal-glucose medium after 7 days, and this may already subject organoids to a state of starvation. Therefore, we included a condition in which organoids received media exchanges every other day (“untreated” control). We found no differentially expressed genes when comparing cones and rods in normal glucose relative to untreated cones and rods (Figure S13). Principal component analysis showed the transcriptome of cones in low glucose to be close to the transcriptome of cones in normal glucose. This was also true for rods. Treatment with HSP90AA1-I-1 induced a strong shift in the transcriptomes of both cones and rods (Figure 6D). CS-KI-1- or CS-KI-1A-treated rods differed markedly from untreated rods, which was not the case for cones. CS-KI-2 and CS-KI-2A both caused minor shifts in photoreceptor transcriptomes (Figure 6D).

Consistent with the principal component analysis, we identified only a limited number of genes differentially expressed between normal- and low-glucose controls: four genes were downregulated in cones, of which *HSPA6* was the only gene also downregulated in rods when comparing normal to low-glucose conditions (Figure S13). Following HSP90AA1-I-1 treatment, cones exhibited differential expression relative to the low-glucose condition in 776 genes, with 169 genes upregulated and 607 genes downregulated. In rods, 423 genes were differentially expressed, including 92 upregulated and 331 downregulated genes (Figure S13). Among these, 47 upregulated and 223 downregulated genes were differentially expressed in both cones and rods. Gene set enrichment analysis (Gene Ontology [GO] terms) revealed that genes involved in RNA processing, which includes transcription, splicing, and translation, were upregulated in both cones and rods treated with HSP90AA1-I-1 compared with the low-glucose controls. Additionally, genes associated with the unfolded protein response were upregulated in cones (Figure S14).

For CS-KI-1, we examined genes that showed differential expression when comparing the transcriptomes of samples treated with CS-KI-1 against those treated with CS-KI-1A and the low-glucose control samples. We sought to identify genes that are differentially expressed in CS-KI-1 compared with low-glucose controls but not in CS-KI-1A and the low-glucose controls. We identified 22 upregulated and 15 downregulated genes in cones, and in rods, we found 39 upregulated and 130 downregulated genes (Figures 6E and S15). Notably, 10 upregulated or downregulated genes were common to both cones and rods. Gene set enrichment analysis revealed that apoptotic genes were downregulated in both rods and cones. Moreover, cones exhibited downregulation of inflammatory response genes, including those in tumor necrosis factor α (TNF- α) signaling via NF-κB and interferon alpha responses. This suggests that the downregulation of both inflammatory and non-inflammatory cell death pathways could be involved in enhancing cone survival (Figure S16).

We adopted a similar approach for CS-KI-2, examining genes differentially expressed when comparing CS-KI-2-treated samples to those treated with CS-KI-2A and to low-glucose controls. Consistent with the principal component analysis, CS-KI-2 treatment resulted in fewer differentially expressed genes. In cones, we identified 10 significantly upregulated genes, with *HMGCS1*, *MSMO1*, *INSIG1*, and *ACAT2* also showing significant upregulation in rods. Additionally, *HTR2C* was found to be downregulated in cones (Figure 6E). Gene set enrichment analysis in both cones and rods indicated that genes involved in mTORC1 signaling and cholesterol homeostasis were upregulated following CS-KI-2 treatment. Furthermore, genes related to fatty acid metabolism showed upregulation in cones (Figure S16).

These findings indicate that treatment with HSP90AA1-I-1, CS-KI-1, or CS-KI-2 induces highly distinct gene expression alterations in rods and cones, each manifesting varying degrees of transcriptomic changes.

### Photoreceptor-saving targets of CS-KI-1 and CS-KI-2

**Figure 7:**
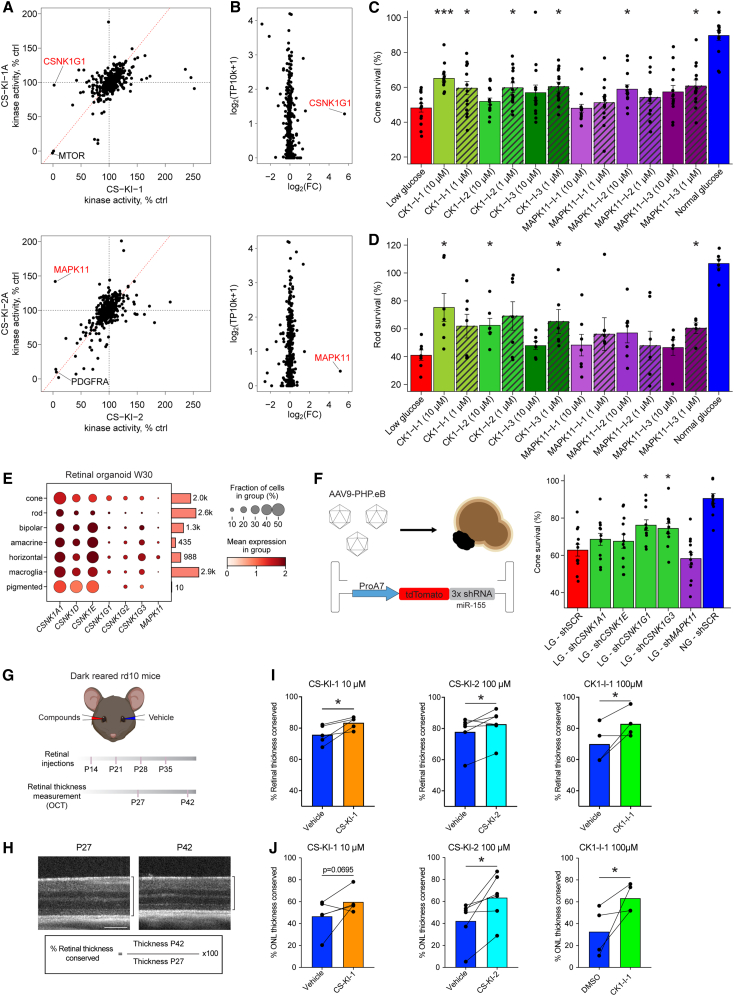
Kinase profiling, CK1 and MAPK11 inhibition with compounds and shRNAs, and compound validation in rd10 mice (A) Kinase profiling of 350 human kinases treated with CS-KI-1, CS-KI-1A, CS-KI-2, or CS-KI-2A. Percentage value of kinase activity compared with a control reaction without inhibitors. Colors indicate the average expression level of the kinase gene in low-glucose organoid cones. Dotted black lines, 100% kinase activity. Dotted red lines, unity line. TP10k, transcript counts per 10,000. (B) Differential kinase activity comparing active compounds and their analogs and average expression levels in organoid cones. FC, fold change. TP10k, transcript counts per 10,000. (C and D) Effects of CK1 and MAPK11 inhibitors on cone (C) and rod (D) survival under low-glucose conditions. Results are shown as mean ± SE with ∗ denoting a *p* value < 0.05 and ∗∗∗ denoting a *p* value < 0.001. Black dots indicate individual organoid cone survival. (E) *CK1* and *MAPK11* expression in human retinal organoids at week 30 (W30) from published single-cell atlases.^16^ Color indicates mean expression in log_2_ transcript counts per 10,000. Dot size indicates the fraction of cells expressing the indicated gene. Right bars and numbers indicate the number of cells from each population used for the analysis. (F) Experimental design and observed cone survival in low glucose after cone-specific downregulation of CK1 and MAPK11. Results are shown as mean ± SE with ^∗^ denoting an adjusted *p* value < 0.05. Black dots indicate individual organoid cone survival. LG, low glucose. NG, normal glucose; SCR, scrambled. (G) Schematic illustration for *in vivo* experiments. Rd10 mice were injected intravitreally with vehicle (DMSO) as a control to the left eye and compound in vehicle to the right eye, both diluted in PBS, weekly from P14 to P35, and retinal thickness was measured with OCT at P27 and P42. (H) Example OCT images at P27 and P42 of control eyes injected with DMSO, and the formula used to calculate the retinal thickness conservation (scale bar, 100 μm). (I) Percentage of retinal thickness conservation between P27 and P42 after weekly injections with the indicated compounds and vehicle. Black dots represent injected eyes, with ∗ denoting a *p* value < 0.05. (J) Percentage of outer nuclear layer (ONL) thickness conservation between P27 and P42 after weekly injections with the indicated compounds and vehicle. Black dots represent injected eyes, with ^∗^ denoting a *p* value < 0.05. See also Figures S17–S25 and Tables S4, S5, S6, and S7.

To investigate causal targets of CS-KI-1 and CS-KI-2 for saving cones or rods, we conducted biochemical kinase profiling for these compounds and their nonfunctional analogs CS-KI-1A and CS-KI-2A. We assessed the activity of 350 human kinases after treatment with each of the four compounds, comparing their activities at 10 μM against a vehicle control (Figures 7A and 7B; Table S4).

CS-KI-1 and its nonfunctional analog CS-KI-1A had little effect on the activities of most of the 350 kinases; however, there were exceptions. As expected, both CS-KI-1 and CS-KI-1A induced complete inhibition of MTOR. Remarkably, the activity of only one kinase, the CSNK1G1, was fully suppressed by CS-KI-1 but not influenced at all by CS-KI-1A (Figures 7A and 7B; Table S4). CSNK1G1 is therefore a potential target for the cone- and rod-saving effects of CS-KI-1 that are independent of MTOR inhibition.

Similarly, the activity of most kinases was not influenced by CS-KI-2 or its nonfunctional analog CS-KI-2A. Both CS-KI-2 and CS-KI-2A induced full inhibition of PDGFRA. The activity of only one kinase, MAPK11, was fully blocked by CS-KI-2 but not inhibited at all by CS-KI-2A. Indeed, CS-KI-2A increased the activity of MAPK11 (Figures 7A and 7B; Table S4). MAPK11 is thus a potential target for the cone- and rod-saving effects of CS-KI-2 that is independent of PDGFRA inhibition.

We tested three known inhibitors of CK1 and MAPK11 on both organoid cones and rods using the two-color organoids (GFP in cones and tdTomato in rods) described above at either 10 or 1 μM inhibitor concentrations. Strikingly, all three CK1 inhibitors significantly improved cone survival at one or both concentrations (CK1-I-1, 10 μM, *p <* 0.001; 1 μM, *p =* 0.04; CK1-I-2, 1 μM, *p =* 0.01; CK1-I-3, 1 μM, *p =* 0.01; *n =* 14, Mann-Whitney U test with Benjamini-Hochberg correction for multiple testing). This was also true for rods (CK1-I-1 10 μM, *p =* 0.01; CK1-I-2 10 μM, *p =* 0.009; CK1-I-3, 1 μM, *p =* 0.009; *n =* 7, Mann-Whitney U test with Benjamini-Hochberg correction for multiple testing). Two out of three MAPK11 inhibitors had a significant positive effect on cone survival (MAPK11-I-2, 10 μM, *n =* 14, *p =* 0.01; MAPK11-I-3, 10 μM, *n =* 13, *p =* 0.01), and one of the MAPK11 inhibitors significantly improved rod survival (MAPK11-I-3, 1 μM, *n =* 7, *p =* 0.009; Mann-Whitney U test with Benjamini-Hochberg correction for multiple testing) (Figures 7C and 7D; Table S5).

*MAPK11* and *CSNK1G1*, along with other CK1-encoding genes, are expressed in both organoid rods and cones, as well as in human retina rods and cones, both in the fovea and the periphery (Figures 7E and S17). Furthermore, the expression of these genes was unaffected by the treatment with CS-KI-1 and CS-KI-2 and glucose starvation (Figure S17). We could also detect the CSNK1G1 protein in multiple cell types in human retinal organoids (Figure S18).

Treatment with CK1 and MAPK11 inhibitors did not affect cone and rod morphology and did not affect the presence and position of other cell types in organoids (Figures S19, S20, and S21).

### Cone-specific, shRNA-mediated knockdown of CSNK1G1 and CSNK1G3 protects cones

Since expression of the *CK1* gene family and *MAPK11* is not restricted to photoreceptors, inhibition of their encoded proteins with small molecules could lead to photoreceptor protection through their action in other cell types. We therefore downregulated the expression of these genes specifically in cones and evaluated the effect on cone survival. We developed three shRNAs for each gene, targeting three different regions, and tested them first in HEK293T cells. Five out of seven shRNA triplets led to downregulation of their target gene (Figure S22). We then constructed AAVs expressing the three shRNAs and tdTomato under the control of the cone-specific promoter ProA7 for each gene whose downregulation was confirmed in HEK293T cells. We transduced organoids with these AAVs. After four weeks, the medium was changed to low-glucose medium, and we imaged organoids at day 0 and day 7. Cone-specific shRNA expression targeting *CSNK1G1* and *CSNK1G3* significantly improved cone survival compared with the scrambled shRNA control (scrambled shRNA, *n =* 12; sh*CSNK1G1*, *n =* 12, *p =* 0.02; sh*CSNK1G3*, *n =* 12, *p =* 0.02; Mann-Whitney U test with Benjamini-Hochberg correction for multiple testing) (Figure 7F). Finally, we confirmed the preserved morphology of the surviving cones treated with shRNAs, as well as the unaltered presence and distribution of other cell types (Figure S23).

### CK1 and MAPK11 inhibition increases cone survival under oxidative stress

The protective effect of the identified compounds could be specific to glucose starvation; therefore, we developed an alternative model of cone death. We induced oxidative stress by exposing the organoids to hydrogen peroxide (H_2_O_2_). We tested several concentrations of H_2_O_2_ in the culture media and measured cone degeneration over 7 days. At an H_2_O_2_ concentration of 50 μM, the mean degeneration rate at day 7 most closely resembled that of glucose starvation (Figure S24). We then tested the CK1 and MAPK11 inhibitors at 1 and 10 μM while measuring cone survival at day 7 in organoids treated with 50 μM H_2_O_2_. CK1-I-1 at both concentrations and MAPK11-I-1 and MAPK11-I-2 at 1 μM significantly increased cone survival (DMSO control, *n =* 8; CK1-I-1, 10 μM, *p =* 0.045, *n =* 8; CK1-I-1, 1 μM, *p =* 0.045, *n =* 8; MAPK11-I-1, 10 μM, *p =* 0.045, *n =* 8; MAPK11-I-2, 1 μM, *p =* 0.045, *n =* 8; Mann-Whitney U test with Benjamini-Hochberg correction for multiple testing) (Figure S24). These results suggest that selected CK1 and MAPK11 inhibitors can prevent cone death across different model systems of induced cone degeneration.

### Protective effect of CS-KI-1, CS-KI-2, and CK1-I-1 in a mouse model of retinitis pigmentosa

To investigate whether some of the photoreceptor-saving compounds also exhibit a protective effect *in vivo*, we used the rd10 mouse model of retinitis pigmentosa, in which photoreceptors progressively degenerate.^59^ We conducted the experiments in dark-reared rd10 mice since the degeneration rate is slower in darkness, which provides a broader temporal window for evaluating the effect of compounds. We injected the left eye intravitreally with 1 μL of vehicle and the right eye with 1 μL compound in vehicle. We injected both eyes weekly from postnatal day 14 (P14) to P35 (Figure 7G). To determine the progression of photoreceptor degeneration, we measured retinal thickness with optical coherence tomography (OCT) at two time points, P27 and P42. We then calculated the percentage of retinal thickness preserved from P27 to P42 under the different conditions in each mouse (Figures 7G, 7H, and S25). Among the tested compounds, three, CS-KI-1 at 10 μM, CS-KI-2 at 100 μM, and CK1-I-1 at 100 μM, showed a significant retinal thickness conservation relative to the vehicle (vehicle, CS-KI-1 10 μM, *p =* 0.016, *n =* 5; vehicle, CS-KI-2 100 μM, *p =* 0.039, *n =* 6; vehicle, CK1-I-1 100 μM, *p =* 0.041, *n =* 4; paired *t* tests) (Figure 7I). These three compounds also had a protective effect when we analyzed the conservation of the thickness of the outer nuclear layer (ONL) (vehicle, CS-KI-1 10 μM, *p =* 0.069, *n =* 5; vehicle, CS-KI-2 100 μM, *p =* 0.013, *n =* 6; vehicle, CK1-I-1 100 μM, *p =* 0.045, *n =* 4; paired *t* tests) (Figure 7J). Therefore, the CS-KI-1 and CS-KI-2 compounds as well as the CK1-specific inhibitor CK1-I-1 that protect photoreceptors in human organoids *in vitro* also protect photoreceptors in a mouse model of retinitis pigmentosa *in vivo*.

## Discussion

We have performed a cell-type-focused compound screen in ∼20,000 human organoids using 2,707 compounds with annotated targets. The results of the primary screen, including the names and unique identifiers of all the compounds, their annotated targets, and their effect on cone survival, are publicly available at https://ConeTargetedCompoundScreen.iob.ch.

We identified two kinase inhibitors that protected cones in the short and longer term, CS-KI-1 and CS-KI-2. We provided evidence suggesting that the currently labeled targets, including MTOR (CS-KI-1) and PDGFRA (CS-KI-2), are not responsible for the effects of CS-KI-1 and CS-KI-2 in cones. By performing a kinase screen using CS-KI-1 and CS-KI-2 and their nonfunctional chemical analogs CS-KI-1A and CS-KI-2A, we identified MTOR- and PDGFRA-independent kinase targets of these compounds: CSNK1G1 for CS-KI-1 and MAPK11 for CS-KI-2. We then showed that inhibitors of CK1 (CSNK1G1 is a member of the CK1 family) and inhibitors of MAPK11 protect both cones and rods. Cone-specific downregulation of different members of the CK1 gene family in organoids using shRNAs suggests a cell-intrinsic role for CSNK1G1 and CSNK1G3 in regulating cone survival. This also suggests that the cone-protective effect of CS-KI-1 originates, at least partially, from cones themselves. A triplet shRNA against MAPK11 targeted to organoid cones did not influence cone survival. One possibility is that the downregulation was not strong enough, a second possibility is that CS-KI-2 acts not on cones but on other cell types, and a third is that MAPK11 may not be a target. However, the finding that MAPK11 inhibitors MAPK11-I-1 and MAPK11-I-2 protected cones argues against the possibility that MAPK11 is not a target of CS-KI-2.

In the organoid screen, we used glucose starvation as a model for cone death. To demonstrate that the cone-saving effect of compounds is not specific to glucose starvation, we performed two sets of experiments, one *in vitro* and one *in vivo*. First, we developed a new *in vitro* model of photoreceptor degeneration induced by oxidative stress (H_2_O_2_) and tested the cone-saving CK1 and MAPK11 inhibitors. Several inhibitors (CK1-I-1, MAPK11-I-1, and MAPK11-I-2) protected cones. Second, we tested several compounds in a mouse model of retinitis pigmentosa *in vivo*. CS-KI-1, CS-KI-2, and CK1-I-1 showed photoreceptor protection. Together, the results of these two sets of experiments suggest that some of the compounds and targets can be generally used to protect photoreceptors.

## Resource availability

### Lead contact

Further information and requests for resources and reagents should be directed to and will be fulfilled by the lead contact, Botond Roska (botond.roska@iob.ch).

### Materials availability

Plasmids and unique reagents generated in this study are available from the lead contact with a completed materials transfer agreement.

### Data and code availability

• Bulk RNA-seq data from organoid photoreceptors treated with cone-saving compounds have been deposited in the European Genome-phenome Archive (EGA) and are accessible under accession number EGA: EGAS50000001626.
• The results of the primary screen, including the names and unique identifiers of all the compounds, their annotated targets, and their effects on cone survival, are publicly available at https://ConeTargetedCompoundScreen.iob.ch.
• This paper analyzes existing, publicly available data from a previous publication,^16^ accessible at the EGA under accession number EGA: EGAS00001004561.
• Additional data are available in Tables S1, S2, S3, S4, S5, S6, and S7.
• Original code used to analyze live imaging data has been deposited at Zenodo: https://doi.org/10.5281/zenodo.18485128 and is publicly available.
• Original code generated to analyze bulk RNA-seq has been deposited at Zenodo: https://doi.org/10.5281/zenodo.18471174 and is publicly available.
• Any additional information required to reanalyze the data reported in this paper is available from the lead contact upon request.

## Acknowledgments

We thank A. Muller, A. Fratzl, T. Dalmay, and P. King for comments on the manuscript. We thank other members of the Roska laboratory for their discussions on the manuscript. The project was supported by an EMBO postdoctoral fellowship ALTF 151-2022 to Á.H.-N. and a European Research Council advanced grant (HURET N 883781), a Swiss National Science Foundation Synergia grant (CRSII3_141801), a Swiss National Science Foundation grant (310030_212186), a Louis-Jeantet Foundation award, a Körber Foundation award, and the NCCR Molecular Systems Engineering grant to B.R. Content from BioRender.com was used for the graphical abstract and some illustrations in Figure 7.

## Author contributions

S.E.S. designed and conducted experiments, including the primary and secondary screens; cultured and prepared organoids, including dissociations; analyzed and interpreted data, including the screening data; and wrote the paper. Á.H.-N. designed and conducted experiments, including shRNA, H_2_O_2_, and *in vivo* experiments; analyzed and interpreted the data; and wrote the paper. L.U. conducted experiments; cultured and prepared organoids, including dissociations; and conducted organoid stainings and live imaging. V.J.A.-M. conducted experiments, including the primary and secondary screens; cultured and prepared organoids; and designed illustrations. Z.R. developed the cone and rod counting algorithms and analyzed data. S.P.-C. analyzed and interpreted RNA-seq data. S.C., O.G., I.G., and I.C. designed and conducted screening experiments and interpreted data. S.R. analyzed and interpreted screening data. M.B. and V.M.-J. conducted *in vivo* experiments. P.T.K. conducted experiments for the ProA330 discovery. A.V. and J.I. produced AAVs. S.M. performed FACS sorting. R.A.S. and V.H. conducted RNA isolation and sequencing. Y.H. performed staining and imaging on organoid sections. T.M.R. contributed to the design of shRNAs. S.P. supervised RNA-seq experiments. M.C. analyzed data. J.J. supervised AAV production and experiments for the ProA330 discovery. C.S.C. supervised RNA-seq analysis. M.D. supervised screening experiments. D.K.B. supervised screening experiments and analysis. M.R. supervised organoid production and screening experiments. V.U. supervised screening experiments. B.R. supervised experiments, analyzed and interpreted data, and wrote the paper.

## Declaration of interests

The Institute of Molecular and Clinical Ophthalmology Basel is the applicant of a pending international patent application that relates to aspects of this manuscript and names S.E.S. and B.R. as inventors.

## STAR★Methods

### Key resources table

**Table:** 

REAGENT or RESOURCE	SOURCE	IDENTIFIER
**Antibodies**		

Mouse monoclonal Anti-Bassoon, 1:800	Enzo	CAT# MA1-20689; RRID: AB_2066981
Mouse monoclonal Anti-ARR3, 1:500	Gift from the Laboratory of Wolfgang Baehr, University of Utah	N/A
Goat polyclonal Anti-NRL, 1:500	R&D Systems	CAT# AF2945; RRID: AB_2155098
Sheep polyclonal Anti-ONECUT2, 1:100	R&D Systems	CAT#AF6294; RRID: AB_10640365
Rabbit polyclonal Anti-SOX9, 1:500	Millipore	CAT# AB5535; RRID: AB_2239761
Rabbit polyclonal Anti-TRPM1, 1:200	Atlas Antibodies	CAT# HPA014779; RRID: AB_2669090
Goat polyclonal Anti-CHAT, 1:300	Millipore	CAT# AB144P; RRID: AB_2079751
Rabbit polyclonal Anti-ARR3, 1:200	Millipore	CAT# AB15282; RRID: AB_1163387
Mouse monoclonal Anti-ARR3 clone 7G6, 1:200	Sigma-Aldrich	CAT# MABN2636; RRID: AB_2935804
Chicken polyclonal Anti-RFP	Rockland	CAT# 600-901-379; RRID: AB_10704808
Lectin from Arachis hypogaea (PNA), 1:1200	Sigma-Aldrich	CAT# L6135; RRID: N/A
Rabbit polyclonal Anti-L/M Opsin, 1:100	Millipore	CAT# AB5405; RRID: AB_177456
Goat polyclonal Anti-hIslet1, 1:200	R&D Systems	CAT# AF1837; RRID: AB_2126324)
Mouse monoclonal Anti-Rhodopsin, 1:400	Sigma-Aldrich	CAT# R5403; RRID: AB_477464
Chicken polyclonal Anti-GFP, 1:1000	Abcam	CAT# ab13970; RRID: AB_300798
Rabbit polyclonal Anti-CSNK1G1, 1:50	Abcam	CAT# ab234727; RRID: N/A
Sheep polyclonal Anti-CRX, 1:200	R&D Systems	CAT# AF7085; RRID: AB_10993572
Rabbit polyclonal Anti-ARR3, 1:100	Atlas Antibodies	CAT# HPA063129; RRID: AB_2732696

**Bacterial and virus strains**		

AAV PHP.eB ProA7-GFP	Jüttner et al.^10^	N/A
AAV PHP.eB ProA330-GFP	This paper	N/A
AAV PHP.eB ProA330-tdTomato	This paper	N/A
AAV PHP.eB ProA7-tdtomato-shSCR	This paper	N/A
AAV PHP.eB ProA7-tdtomato-shCSNK1A1	This paper	N/A
AAV PHP.eB ProA7-tdtomato-shCSNK1E	This paper	N/A
AAV PHP.eB ProA7-tdtomato-shCSNK1G1	This paper	N/A
AAV PHP.eB ProA7-tdtomato-shCSNK1G3	This paper	N/A
AAV PHP.eB ProA7-tdtomato-shMAPK11	This paper	N/A

**Chemicals, peptides, and recombinant proteins**		

For detailed information about compounds used for the screening see Tables S1, S2, S3 and S5 and corresponding methods section	This paper	N/A
Mode-of-action (MOA) compound library	Canham et al.^51^	N/A
Hoechst 33342	Thermo Fisher	CAT# 62249
mTeSR1 medium	STEMCELL Technologies	CAT# 85850
Matrigel Matrix	Corning	CAT# 356230
Costar 6-well Clear TC-treated Multiple Well Plates	Corning	CAT# 3506
UltraPure 0.5M EDTA, pH 8.0	Invitrogen	CAT# 15575020
Accutase - Enzyme Cell Detachment Medium	Invitrogen	CAT# 00-4555-56
MicroTissues 3D Petri Dish micro-mold spheroids	Sigma-Aldrich	CAT# Z764000
TopVision Agarose	Thermo Scientific	CAT# R0491
Costar 12-well Clear TC-treated Multiple Well Plates	Corning	CAT# 3513
DMEM/F-12, GlutaMAX supplement	Gibco	CAT# 31331‒028
N-2 Supplement (100X)	Gibco	CAT# 17502‒048
MEM Non-essential Amino Acid Solution (100X)	Sigma-Aldrich	CAT# M7145
Heparin sodium salt from porcine intestinal mucosa	Sigma-Aldrich	CAT# H3149
DMEM, high glucose, GlutaMAX Supplement, pyruvate	Gibco	CAT# 10569‒010
Ham's F-12 Nutrient Mix, GlutaMAX Supplement	Gibco	CAT# 31765‒027
B-27™ Supplement (50X), minus vitamin A	Gibco	CAT# 12587010
Penicillin-Streptomycin (10,000 U/mL)	Gibco	CAT# 15140‒122
Nunc Non-treated Flasks	Thermo Scientific	CAT# 159926
Anti-Adherence Rinsing Solution	STEMCELL Technologies	CAT# 07010
Fetal Bovine Serum	Sigma-Aldrich	CAT# ES-009-B
Taurine	Sigma-Aldrich	CAT# T0625
Retinoic acid, all-*trans*	Sigma-Aldrich	CAT# R2625
Sterilin Standard 90mm Petri Dishes	Thermo Scientific	CAT# 101VR20
Poly(ethylene glycol) diacrylate (PEGDA)	Sigma-Aldrich	CAT# 455008
Porcine Gelatin	Sigma-Aldrich	CAT# G1890
Sucrose	Sigma-Aldrich	CAT# 84100
Lithium phenyl-2,4,6-trimethylbenzoylphosphinate (LAP)	Sigma-Aldrich	CAT# 900889
Donkey Serum	Sigma-Aldrich	CAT# S30-M
Bovine Serum Albumin	Sigma-Aldrich	CAT# 05482
Triton™ X-100	Sigma-Aldrich	CAT# T9284
Sodium azide	Sigma-Aldrich	CAT# S2002
TWEEN 20	Sigma-Aldrich	CAT# P9416
ProLong Gold Antifade Mountan	Invitrogen	CAT# P36934
Citrate Buffer, pH 6.0, 10X, Antigen Retriever	Sigma-Aldrich	CAT# C9999
Polystyrene CellSTACK - 5 Chamber with Vent Caps	Corning	CAT# 3319
PEI MAX - Transfection Grade Linear Polyethylenimine Hydrochloride (MW 40,000)	Polysciences	CAT# POL24765-1
DMEM, high glucose	Gibco	CAT# 11965-092
Millipore Stericup Quick Release Vacuum Filtration System	Millipore	CAT# S2GPU02RE
Turbonuclease	Accelagen	CAT# N0103L
POROS GoPure AAVX Pre-packed Column	Thermo Scientific	CAT# A36652
Glycine	Millipore	CAT# 1.04201.1000
L-Arginine	Sigma-Aldrich	CAT# A5006
Sodium chloride	Sigma-Aldrich	CAT# 31434
Trizma base	Sigma-Aldrich	CAT# 93350
Pluronic F-68	Thermo Scientific	CAT# 24040032
Amicon Ultra Centrifugal Filter	Millipore	CAT# UFC910096
TaqMan Fast Advanced Master Mix for qPCR	Applied Biosystems	CAT# 4444557
Proteinase K	Thermo Scientific	CAT# 11501515
Corning 96-well Clear Round Bottom Ultra-Low Attachment Microplate	Corning	CAT# 7007
DMEM, no glucose	Gibco	CAT# 11966025
384-well low dead volume plates	Labcyte	CAT# LP-0200
96-well polypropylene U-bottom microplates	Greiner	CAT# 65026
pluriStrainer Mini cell strainer, mesh size 70 μm	pluriSelect	CAT# 43-10070
Sera-Mag Magnetic carboxylate modified particles (Hydrophylic)	Cytiva	CAT# GE24152105050250
Hydrogen peroxide solution	Sigma-Aldrich	CAT# 216763

**Critical commercial assays**		

Glucose Colorimetric Detection Kit	Invitrogen	CAT# EIAGLUC
Neural Tissue Dissociation Kit (P)	Miltenyi Biotec	CAT# 130-092-628
SuperScript™ IV Reverse Transcriptase	Thermo Fisher	CAT# 18090200
KAPA HiFi HotStart	Roche	CAT# 07958935001
KAPA HiFi PCR Kit	Roche	CAT# 07958846001
PanQinase assay method	Reaction Biology Corporation	N/A
RNA with RNeasy Plus Mini Kit	Qiagen	CAT# 74134
FastStart Universal SYBR Green Master (Rox)	Roche	CAT# FSUSGMMRO
High-Capacity cDNA Reverse Transcription Kit	Applied Biosystems	CAT# 4368814

**Deposited data**		

Bulk RNA-seq of rods and cones from human retinal organoids treated with different compounds and control conditions	This paper, deposited at EGA	EGA: EGAS50000001626
Summary of the primary screen dataset	This paper	https://ConeTargetedCompoundScreen.iob.ch

**Experimental models: Cell lines**		

hiPSC line 01F49i-N-B7	Cowan et al.^16^	RRID:CVCL_C1TR
Adherent HEK293T cells	ATCC	ATCC Cat# CRL-3216, RRID:CVCL_0063

**Experimental models: Organisms/strains**		

Mouse: C57BL/6J	Jackson laboratories	RRID: IMSR_JAX:000664
Mouse: rd10: B6.CXB1-Pde6b^rd10^/J	Jackson laboratories	RRID: IMSR_JAX:004297

**Oligonucleotides**		

See Table S6	This paper	N/A

**Recombinant DNA**		

pAAV-ProA7-GFP	Jüttner et al.^10^	N/A
pAAV-ProA330-GFP	This paper	N/A
pAAV-ProA330-tdtomato	This paper	N/A
pAAV-ProA7-tdTomato-shSCR	This paper	N/A
pAAV-ProA7-tdTomato-shCSNK1A1	This paper	N/A
pAAV-ProA7-tdTomato-shCSNK1E	This paper	N/A
pAAV-ProA7-tdTomato-shCSNK1G1	This paper	N/A
pAAV-ProA7-tdTomato-shCSNK1G3	This paper	N/A
pAAV-ProA7-tdTomato-shMAPK11	This paper	N/A
pAAV-EF1a-tdTomato-shCSNK1A1	This paper	N/A
pAAV-EF1a-tdTomato-shCSNK1D	This paper	N/A
pAAV-EF1a-tdTomato-shCSNK1E	This paper	N/A
pAAV-EF1a-tdTomato-shCSNK1G1	This paper	N/A
pAAV-EF1a-tdTomato-shCSNK1G2	This paper	N/A
pAAV-EF1a-tdTomato-shCSNK1G3	This paper	N/A
pAAV-EF1a-tdTomato-shMAPK11	This paper	N/A
pcDNA3.1-CMV-CSNK1A1-P2A-GFP	This paper	N/A
pcDNA3.1-CMV-CSNK1D-P2A-GFP	This paper	N/A
pcDNA3.1-CMV-CSNK1E-P2A-GFP	This paper	N/A
pcDNA3.1-CMV-CSNK1G1-P2A-GFP	This paper	N/A
pcDNA3.1-CMV-CSNK1G2-P2A-GFP	This paper	N/A
pcDNA3.1-CMV-CSNK1G3-P2A-GFP	This paper	N/A
pcDNA3.1-CMV-MAPK11-P2A-GFP	This paper	N/A
pHGT1-Adeno1 helper plasmid	Provided by C. Cepko, Harvard Medical School. Boston, MA, USA	N/A

**Software and algorithms**		

ImageJ	Schindelin et al.^60^	https://imagej.net/software/fiji/
GraphPad Prism 10 (Version 10.4.2)	GraphPadSoftware	https://www.graphpad.com/
ChemDraw	Revvity Signals	https://revvitysignals.com/products/research/chemdraw
BLOCK-iT	Thermo Fisher	https://rnaidesigner.thermofisher.com/rnaiexpress/setOption.do?designOption=shrna
QuantStudio Design and Analysis Software (v1.5.3)	Thermo Fisher	https://www.thermofisher.com/ch/en/home/technical-resources/software-downloads/quantstudio-3-5-real-time-pcr-systems.html
Bioptigen InVivoVue v2.4.33	Leica	N/A
Photoreceptor detection and quantification code	This paper	Zenodo: 10.5281/zenodo.18485128
Bulk RNA-seq analysis pipeline	This paper	Zenodo: 10.5281/zenodo.18471174
Snakemake v7.21.0	Mölder et al.^61^	https://github.com/snakemake/snakemake
STAR v2.7.10b	Dobin et al.^62^	https://github.com/alexdobin/STAR
DESeq2 v1.38	Love et al.^63^	https://bioconductor.org/packages/release/bioc/html/DESeq2.html
scikit-learn v.1.2.2	Pedregosa et al.^64^	https://github.com/scikit-learn/scikit-learn
GSEApy v1.0.5	Fang et al.^65^	https://github.com/zqfang/GSEApy
GenomicFeatures v1.50.2	Lawrence et al.^66^	https://bioconductor.org/packages/release/bioc/html/GenomicFeatures.html
pandas v2.0.1	McKinney^67^	https://github.com/pandas-dev/pandas
scanpy	Wolf et al.^68^	https://github.com/scverse/scanpy

**Other**		

Information regarding key equipment and devices is listed in the corresponding methods section	N/A	N/A

### Experimental model and study participant details

#### Mice

All animal procedures were approved by the Veterinary Department of the Canton of Basel-Stadt, and conducted in accordance with FSVO Ordinance on Laboratory Animal Husbandry, the Production of Genetically Modified Animals and Methods of Animal Experimentation (Swiss Animal Experimentation Ordinance) SR 455.163. Mouse experiments were carried out on P14 to P43 homozygous rd10 mice with C57BL/6J background purchased from The Jackson Laboratory (B6.CXB1-Pde6b^rd10^/J). Mice were housed in dark cages from birth until euthanasia and were handled under dim red light. The animals were provided with standard mouse chow and water ad libitum.

### Human induced pluripotent stem cells

Organoids were derived from the induced pluripotent stem cell line 01F49i-N-B7.^16^ The cells were cultured at 37°C and 5% CO_2_ in a humidified incubator, using mTesR1 medium (STEMCELL Technologies, #85850) on Matrigel-coated (Corning, #356230) 6-well plates (Corning, #3506). The culture medium was replaced daily and cells were passaged weekly using 0.5 mM EDTA (Invitrogen, #15575020) in PBS without CaCl_2_/MgCl_2_ applied for 3-5 min to facilitate detachment of cells as small clumps for subsequent seeding.

### Method details

#### Generation of retinal organoids

Retinal organoids were generated as previously described from the induced pluripotent stem cell line 01F49i-N-B7,^16^with the modifications given below. All experiments in this study were performed using organoids that were 30- to 32 weeks old.

#### Embryoid body formation and culture

Induced pluripotent stem cells were detached and a single-cell suspension was created using 0.5 mM EDTA (Invitrogen, #15575020) for 3 min, followed by a 3-min Accutase (Thermo Fisher Scientific, #00-4555-56) treatment at 37°C. Embryoid body formation took place in 256 microwell-hydrogels with ∼250-300 cells seeded per microwell. The hydrogels were generated using a MicroTissues 3D Petri Dish micro-mold (Sigma Aldrich, Z764000) and 2% agarose (Thermo Fisher Scientific, #R0491). Each hydrogel was cultured in a 12-well plate (Corning, #3513) in neural induction medium DMEM / F12 (GIBCO, #31331‒028), 1% N2 Supplement (GIBCO, #17502‒048), 1% NEAA Solution (Sigma, #M7145) and 2 mg/mL heparin (Sigma, #H3149) for one week with daily medium exchanges. Embryoid bodies that formed in one 256 microwell-hydrogel were detached from the hydrogel and distributed evenly across three wells of a Matrigel (Corning, #356230)-coated 6-well plate (Corning, #430166).

#### Early organoid culture and checkerboard scraping

Organoids in 6-well plates cultured with daily medium exchanges started to form 2D confluent structures. For the first 16 days, they were cultured in neural induction medium. The medium was subsequently changed to 3 parts DMEM (GIBCO, #10569‒010): 1 part F12 medium (GIBCO, #31765‒027) (‘3:1 medium’), supplemented with 2% B27 without vitamin A (GIBCO, #12587010), 1% NEAA Solution (MERCK, #M7145), and 1% penicillin / streptomycin (GIBCO, #15140‒122). Checkerboard scraping was performed between days 28 and 30 of culture as described previously.^16^

#### 3D-organoid culture

Aggregates from four wells of a 6-well plate were transferred to one 175 cm^2^ tissue culture flask (Thermo Scientific, #159926) previously treated with an anti-adherence solution (STEMCELL Technologies, #07010). Flasks containing organoids were filled with 35 - 45 mL of medium, which was replaced 1-3 times per week. The flasks were maintained in 3:1 medium for 6 weeks of culture. The medium was supplemented subsequently with an additional 10% FBS (Millipore, # ES-009-B) and 100 μM Taurine (Sigma, #T0625) until week 10. Until week 14, the medium was further supplemented with 1 μM retinoic acid (Sigma, #R2625). After this period, the retinoic acid concentration was reduced to 0.5 μM and the B27 supplement was replaced with N2 supplement for the remaining duration of the culture. For easy access to organoids for experiments, they were transferred to round cell culture dishes (Thermo Fischer, #101VR20). Aggregates lacking neuroepithelium were removed just before the experiments.

#### Organoid fixation, sectioning and staining

Organoids were fixed in paraformaldehyde for 4 h at 4°C and washed three times for 10 min each in PBS. They were then submerged in PBS containing 30% sucrose for cryopreservation. Fixed organoids were stored at -80°C.

For sectioning, the organoids were embedded in a solution of 7.5% gelatine and 10% sucrose in PBS. For high-throughput sample embedding, we used the HistoBrick protocol.^69^ Briefly, we used 8 v% Poly(ethylene glycol) diacrylate (PEGDA) (Mw = 700, Sigma Aldrich, #455008), 2.5 wt% gelatine (Sigma Aldrich, #G1890), 10 wt% sucrose (Sigma Aldrich, #84100) and 0.05 wt% Lithium phenyl-2,4,6-trimethylbenzoylphosphinate (LAP; Sigma Aldrich, #900889) in 1 × PBS as embedding material. First, we filled the HistoBrick mold with the embedding material, crosslinked it by exposure to 365 nm wavelength light with a total illumination dose of 1.05 J/s^∗^cm (Lightning enterprises, #UV9W-2). The organoid samples were incubated in the embedding material for 15 min on a heating plate at 40°C, then they were transferred into the HistoBrick wells, followed by another crosslinking. The embedded samples were then frozen and sectioned into 25-μm-thick slices using a cryostat (MICROM International, #HM560).

Immunostaining was carried out as described previously.^16^ Briefly, slides were dried for 30 min at room temperature, then rehydrated in PBS for 5-10 min. They were then blocked with a solution containing 10% normal donkey serum (Sigma, #S30), 1% BSA (Sigma, #05482), 0.5% Triton X-100 (Sigma, #T9284), and 0.02% sodium azide (Sigma, #S2002‒25G) at room temperature for 1 h. Sections were then treated with primary antibodies (see Key Resources Table) in a similar blocking solution but with 3% normal donkey serum for 24 h. After three washes in PBS with 0.1% TWEEN 20 (Sigma, #P9416) for 10 min each, the slides were exposed to secondary antibodies (Thermo Fisher Scientific, donkey secondary antibodies conjugated to Alexa Fluor 488, 568, or 647) diluted 1:500 and Hoechst 33342 (Thermo Fisher, #62249) diluted 1:10,000 in the same buffer as the primary antibodies for 2 h. This was followed by two 10-min washes in PBS with 0.1% Tween and a 15-min wash in PBS. Slides were finalized with ProLong Gold (Thermo Fisher Scientific, #P36934) before sealing. For CSNK1G1 antibody, an antigen retrieval step was needed. First, the immunostaining for all antibodies was done as described above. Then, the slides were fixed with 4% PFA for 15 min at room temperature. Afterwards, the slides were heated in 1x citrate buffer pH6.0 (Sigma Aldrich, #C9999) in PBS for 15 min at 98°C. Then the immunostaining of all primary and secondary antibodies was performed again as described above.

#### Imaging stained cryosections

Images of representative regions of the organoids were captured using a spinning disc confocal microscope (Olympus IXplore SpinSR). The microscope was adjusted to either 20x or 40x magnification and images were taken across multiple Z-planes. All captured images are shown as maximum intensity projections.

#### AAV production

Adherent HEK293T cells (ATCC, #CRL3216) were cultured in a 5-layer CellSTACK (3,180 cm^2^; Corning, # CLS3319) for AAV vector production. These cells were co-transfected with an AAV transgene plasmid, an AAV helper plasmid encoding the AAV Rep2 and Cap proteins for the selected AAV9-PHP.eB capsid,^52^ and the pHGT1-Adeno1 helper plasmid carrying adenoviral genes (kindly provided by C. Cepko, Harvard Medical School, Boston, USA) using PEIMAX (Polyscience, #POL24765-1). Plasmids were mixed in 98 mL DMEM (Thermo Fischer, #11965-092) and incubated for 5 min. PEIMAX was then added to the DMEM-diluted DNA. After an additional 10-min incubation, the DNA-PEIMAX complex was added to the cells. After 60 h, the culture medium was supplemented with 250 mL fresh DMEM containing 1% Pen-Strep (Thermo Fischer, #15140-122). AAV vectors present in cells and the culture medium were harvested approximately 5 days post-transfection.

#### Purification of AAVs

AAVs were purified either from cell culture medium alone or from both cells and cell culture medium. The cell culture supernatant was first cleared by centrifugation at 1400 x g for 15 min (5920R; Eppendorf) and then filtered through a 0.45-μm PES filter (Merck Millipore, #S2GPU02RE). The cell pellet was resuspended in 11 mL lysis buffer (150 mM NaCl, 20 mM Tris-HCl pH 8.0) and subjected to three freeze-thaw cycles. To remove cell debris, the cell lysate was centrifuged at 4,000 x g for 30 min and the resulting supernatant filtered through a 0.45-μm filter.

Both the filtered cell culture medium and the cell lysate were treated with Turbonuclease (Accelagen, #N0103L) at 50 U/mL for 1 h at 37°C. The sample was then loaded onto an affinity column (POROS CaptureSelect AAVX; ThermoFisher, #A36652) and eluted with a solution of 0.1 M glycine (Merck, #1.04201.1000), 0.25 M arginine (Sigma, A5006), 0.2 M NaCl (pH 2.7; Sigma, 31434), following an extensive wash with 20 times the column volume of 500 mM NaCl, 50 mM Tris at pH 7.3 (Merck, 93350), and 0.01% Pluronic F-68 (Thermo, #24040032). The eluted AAVs were immediately neutralized with 1/11 volume of 1 M Tris-HCl pH 10. The purified AAV vectors were then concentrated as needed in sterile PBS + 0.001% Pluronic F-68 using a spin filter (Amicon Ultra Centrifugal Filter Units; Millipore Sigma, UFC910096; molecular cutoff 100 kDa).

#### AAV titration

Encapsidated viral DNA was quantified using TaqMan RT-PCR (Thermo Fischer, #4444557) targeting the ITR sequences (Table S6) relative to a linearized ITR-containing plasmid as a standard. Prior to quantification, AAV particles were denatured using Proteinase K (Thermo Fischer, #11501515). The titers were then calculated and expressed as genome copies per mL.

#### AAV transduction of organoids in 96-well plates

For small-scale experiments (Figures 6, 7, S9, S11, S19–S21, S23, and S24), individual organoids were transferred to a single well of an ultra-low attachment U-bottom 96-well plate (Corning, #7007) after 25 to 26 weeks of maturation. AAVs were diluted in culture medium to a concentration of 3.3 x 10^12^ genome copies per mL. The existing medium in the wells was removed from the organoids and replaced with 30 μL of the AAV solution. After an incubation period of 4-5 h, an additional 70 μL of medium was added per well. After 24 h, 100 μL of medium was added on top per well. After a further 24 h, 150 μL of medium was replaced per well. Transduced organoids were then cultured for 4 to 5 weeks at 37°C and 5% CO_2_ before the onset of further experiments. During this period, the medium (150 μL per well) was replaced 3 times per week.

#### Bulk AAV transduction of organoids

For large-scale experiments (Figures 1, 2, 3, 4, 5, 6, S1–S8, and S10), organoids were transduced with AAVs in their original flask. AAVs were diluted in culture medium to a concentration of 1 x 10^13^ to 2.5 x 10^13^ genome copies per mL. The flasks were placed upright and the medium aspirated from the organoids. AAV solution was then added at 8 mL per flask and the flasks incubated in the upright position for 24 h. Following this, 32 mL of medium was added to each flask and the flasks were then laid flat. After a further 24 h, the medium was exchanged completely. The transduced organoids were cultured for an additional 4-5 weeks at 37°C and 5% CO_2_ with a medium exchanged once a week.

#### Low-throughput imaging

All non-screening imaging (Figures 1, 4G–4I, 5, 6, 7, S9, S11, S19–S21, S23, and S24) was conducted using a spinning disk confocal microscope (Olympus IXplore SpinSR) with a 4x or a 10x objective. For live imaging, organoids were kept in a humidified chamber maintained at 37°C with 5% CO_2_. The contrast and brightness settings for images captured from the same organoid across different timepoints were the same.

#### Glucose starvation

After 30 weeks of maturation and four weeks after AAV transduction, organoids were transferred into an ultra-low attachment U-bottom 96-well plate (Corning, #7007) with 150-200 μL medium. The remaining medium was then reduced to approximately 60 μL per well.

The low glucose starvation medium was composed of 3:1 medium supplemented with an additional 10% heat-inactivated FBS (Millipore, #ES‒009‒B), N2 supplement (GIBCO, #17502‒048), 100 μM taurine (Sigma, #T0625), and 0.5 μM retinoic acid. Instead of the standard DMEM, DMEM with no glucose (Thermo Fisher, #11966025) was used. The normal glucose medium was prepared similarly but with regular DMEM containing 25 mM glucose (Thermo Fisher, #10569010). The low glucose medium still contained a small amount of glucose due to the F12 medium (GIBCO, #31765‒027) and possibly the FBS.

Prior to the addition of the respective experimental conditions, organoids including the normal glucose controls were washed twice with 120 μL of low glucose medium. This process was conducted manually or, for screening experiments, with a 96-well head Selma pipettor (Cybio, #OL7001-26-212) fitted with 60-μL tips (Cybio, #OL3800-25-735-P).

#### Glucose consumption measurement

Organoids were transferred to an ultra-low attachment U-bottom 96-well plate (Corning, #7007) and subjected to glucose starvation. On each measurement day, 5 μL of medium was collected from the 180 μL of medium for each of the 10 organoids per condition. For the initial measurement, which involved only medium without organoids, three replicates were taken. The glucose concentration was subsequently determined using a Glucose Colorimetric Detection Kit (Invitrogen, #EIAGLUC). The assay results were read using a Hidex Sense Microplate Reader.

#### Compound preparation, dilution, and addition

A set of 2,707 annotated compounds selected for screening on retinal organoids was sourced from the Mode-of-action (MOA) compound library.^51^ The compounds, originally at a stock concentration of 10 mM in 100% DMSO, were plated in 384-well low dead volume plates (Labcyte, #LP-0200). Using the ECHO acoustic liquid handler (Labcyte, #Echo 555), 225 nL of the compounds was transferred into sterile 96-well polypropylene U-bottom microplates (Greiner, #65026). These plates were stored overnight at 4°C in a confined environment to prevent evaporation.

The following day, the plates were brought to room temperature and the compounds diluted 666 times by adding 150 μL of low glucose medium with the multidrop combi dispenser (Thermo Scientific, #5840300). Using a 96-well head Selma pipettor (Cybio, #OL7001-26-212) equipped with 60-μL tips (Cybio, #OL3800-25-735-P), 120 μL of culture medium was removed from the ultra-low attachment U-bottom 96-well assay microplates (Corning, #7007) containing the organoids. Then, using the same pipettor, 120 μL of compounds diluted in medium were pipetted from the intermediate 96-well plates into the plates containing the retinal organoids. The assay plates were then incubated (5% CO_2_; 37°C; humidified environment) in an automated incubator (Thermo, #incubator Cytomat 10 C 450) until imaging. Each compound was tested in five replicates at a final concentration of 10 μM. All vehicle controls were 0.1% DMSO in low glucose or normal glucose medium. In the secondary screens, the compounds were tested at four different concentrations (10; 1; 0.1 and 0.01 μM) using the compound transfer process as in the primary screen.

HSP90AA1-I-1, CS-KI-1 and CS-KI-1A (Figures 4G, 4H, 5C, 5D, 6, and S9, and S12–S15) were newly synthesized by Enamine. CS-KI-2 and CS-KI-2 were purchased from MolPort (Figures 4I, 5D, 6, S9, and S12–S15). These were dissolved in 90% DMSO prior to manual dilution in low glucose medium. The vehicle controls for these experiments involved 0.09% DMSO. If not indicated otherwise HSP90AA1-I-1 was added at a concentration of 1 μM and CS-KI-1, CS-KI-1A, CS-KI-2 and CS-KI-2A at a concentration of 10 μM. All CK1 and MAPK11 inhibitors were purchased from MedChemExpress or StemCell Technologies.

#### Automated imaging

Confocal images for all screening experiments were captured using a 4x objective lens (Olympus UPLSAPO, NA=0.16) on an automated spinning disk confocal microscope equipped with a sCMOS camera (Yokogawa, CV7000). The samples were maintained in a 5% CO_2_ and 37°C environment during acquisition. Images were acquired at 24 different confocal planes, each separated by a 34-μm interval, to cover the entire organoid. This was followed by the acquisition of a stack of seven brightfield images at 100-μm intervals. After image acquisition, each plate was incubated (5% CO_2_; 37°C; humidified environment) for seven days and then re-imaged using the same procedure.

#### Promoter ProA330

The general design and the testing of ProA series promoters have been described previously.^10^ The AAV serotypes used were AAV9-PHP.eB^52^ for human retinal organoids and AAV8-BP2^70^ for mouse injections. This new promoter has the following sequence:

AACCCAAGAAATTACAGGCTGAAACCAGAAAAGAACACATTAAAGCACCAAGAGAAAGTTGGAGTGGGTTGAAGGGAAACAGATTTTTAAAGTTAAGGCTCTGTGAAATGGGTAGAATTAACTACAGGTTAAAAATAAAATGTTAACTAAAGGTTGCCTCTGAGTAACAGGATTATGGGTGATTTTAATTGTCTTCTTTGTGTATGTTCAACAGTGACTATAATATGTATTACTTTTGGAATAAAGGAAAACCTGAAAGGTGTGTTGTTTTATAAGGGCCCTTAGGTTGCCAAAATTAGAGTCATTGAAATCTAAAGCTGATAAAAACTTTAGTGCAAAGATTGTGACATGGGAGACTACACATACCAGATCCATAATGTACATGAGGACAGTAGGCCGAGGGGCCCTGCACATTGAAAGCCCACATGGGAGAAGCCCTTGGGAAGGGGAGTGGAAGGATGAGGCAAGGGGCCGGGGGGATGCAGAGGCTGGCAGGCAGTCATTTCTCAGCTTCAGCCATTCCCGCCATGGGGGAATGTGGACAGAGAAGCCAAACAAATCTCCTAAACAGTAAATGTCAGTCTTCTGTGTCAGATATTTAAGAAAACTAACAGAGGTCAGAGAAGACACACCTACAGCAAGTAGACTGTCCCTGTGCTGCCTTTTTGCAACCCCTGCTTTGGCAGTGCTCAAGCCCACCTCCTGCTCTGTGCAGACATCTCTTCTTTGCTCTTACTAGACCAAGGTGAAAGAAAACTCTCACCTTCTCCCATCTGGCCCCACAGCATCTGGAACACACTGATCCTCATAATCCTTGTTCTTGAGAAATATTAATGACTTAATCTCCCAAGCTTGCTCCCTCTCCTGTGCAGGCCATCTCAGTATGTTTTGCAGACAAGACCCAGAGAAGTCCAGACTGGACTTGTTGCAGACTGCAAAACTGCCATTGGAAGGCCTCCGTCCCAGTCCTTCTACAGAGTAGCCAGTGGGATTCCCAGCC

#### Organoid dissociation and FACS-sorting for RNA-seq

For bulk RNA sequencing, organoids at week 26 were co-transduced with a ProA7-GFP^10^ construct (cone-specific promoter driving GFP expression) and ProA330-tdTomato (rod-specific promoter driving tdTomato expression). Four weeks post-transduction, organoids were subjected to their corresponding treatments (untreated, normal glucose, low glucose, and low glucose with either HSP90AA1-I-1, CS-KI-1, CS-KI-1A, CS-KI-2 and CS-KI-2A) in 96-well plates. After seven days of treatment, individual organoids were dissociated using the reagents from the Neural Tissue Dissociation Kit (P) (Miltenyi Biotec, #130-092-628).

Each organoid was transferred to a 1.5-mL Eppendorf tube, washed once with 1 mL PBS, and then with 1 mL of provided Buffer X. Subsequently, 25 μL of provided Enzyme P solution was diluted in 1 mL Buffer X and added to the organoids. Organoids were then incubated in the enzyme solution at 37°C with agitation at 900 rpm for 25 min. During this incubation period, the organoids were pipetted up and down using a 1-mL pipette every 5 min to assist dissociation. Then 5 μL of Enzyme A together with 10 μL of Buffer Y were added to the partially dissociated organoids, followed by a 15-min incubation at 37°C without shaking. Thereafter the cells from the fully dissociated organoids were handled on ice. The dissociated cells were centrifuged at 300 x g for 5 min at 4°C to pellet the cells and remove residual enzyme solution. The cell pellet was then resuspended in 250 μL PBS and passed through a 70-μm filter (pluriSelect, 43-10070). Prior to FACS-sorting, Hoechst 33342 (Thermo Fisher Scientific, #62249) was added to the cell suspension at a 1:10,000 dilution. This was done to allow exclusion of debris from nucleated cells during FACS.

FACS sorting was performed using a FACSAria (BD Biosciences). Up to 500 fluorescent cones and rods were sorted directly into guanidine lysis buffer (0.25 M GuHCl, 24 μM dNTPs, 1.8 μM oligo-dT, 1.2 μM DTT, 1 M Betaine) for subsequent RNA extraction and immediately frozen at -70°C for storage.

#### Bulk RNA-sequencing

Cell lysates were processed following the bulk FLASH-seq protocol.^71^^,^^72^ Briefly, RNA was converted to cDNA fragments using Superscript IV (Thermo Fisher Scientific, #18090200) and amplified with KAPA HiFi HotStart (Roche, #KK2602). The cDNA was then cleaned using a 0.8x ratio of homebrew SeraMag beads in 18% PEG (Cytivia, #GE24152105050250). cDNA concentration and quality were measured using Qubit (Thermo Fisher Scientific, #Q33231) and an Agilent Bioanalyzer (Agilent, #5067-4626). The cDNA was normalized to 200 pg/μL before tagmentation using 0.2 μM of homemade Tn5. Tn5 transposase was produced by the EPFL Protein Facility (Lausanne, Switzerland). The reaction was halted with 0.2% SDS. An indexing PCR was performed to add Nextera index adapters (1 μM, Integrated DNA Technology) using KAPA HiFi reagents (Roche, #KK2102). Libraries were pooled in equal volumes and a final 0.8x cleanup performed with homebrew SeraMag beads before measuring sample concentration and quality. The library pool was normalized and sequenced on Illumina NextSeq MO flowcell (75-8-8-75) at approximately 1 million reads/sample. Basecalling and demultiplexing were performed with bcl2fastq (v2.20, Illumina Inc.).

#### Kinase profiling

CS-KI-1, CS-KI-1A, CS-KI-2, and CS-KI-2A were subjected to *in vitro* kinase profiling against a panel of 350 human wild-type kinases. This profiling was conducted by the Reaction Biology Corporation using their PanQinase assay method, a plate-based assay that measures kinase activity through the transfer of radioactively labeled ATP onto a specific substrate. An analogs assay has been described before.^73^ Briefly, kinase/substrate pairs and cofactors were prepared in a specific buffer, introducing the compounds at a concentration of 10 μM, followed by the addition of a mix of ATP and 33P ATP after approximately 20 min. For detailed assay conditions, refer to Table S7. The reactions, maintained at 25°C for two hours, were then applied to P81 ion exchange filter papers. Subsequent washing removed unbound phosphate and kinase activity was quantified by comparing the activity in test samples against vehicle (DMSO) controls, adjusted for background from inactive enzyme controls. Detailed experimental conditions are provided in Table S7.

### Quantification and statistical analysis

All quantifications, statistical analyses, and plots were executed using R, Python, ImageJ or Graphpad Prism. All illustrations were created using Adobe Illustrator, while chemical structures were rendered with ChemDraw.

If not stated otherwise, ‘n’ always refers to the number of organoids per condition. The p-values depicted in the figures are not corrected for multiple testing if not indicated otherwise. A summary of the primary screen dataset can be found at https://ConeTargetedCompoundScreen.iob.ch.

#### Promoter specificity and efficacy analysis

Promoter specificity and efficacy quantification was performed using ImageJ. Maximum intensity projections were calculated and cells were then manually counted using the ImageJ plugin, Cell Counter.

For the ProA7-GFP construct (Figure 1E), five different organoids were analyzed. The specificity was determined by calculating the percentage of all GFP-positive cells that were also ARR3-positive. Efficacy was determined by calculating the percentage of all ARR3-positive cells that were also GFP-positive.

For the ProA330-GFP construct (Figure 5B), three different organoids were analyzed using the methods used for ProA7-GFP. Specificity was determined by calculating the ratio of all GFP-positive cells located in the outer nuclear layer that were not ARR3-positive. Efficacy was determined by calculating the percentage of GFP-positive and ARR3-negative cells among all cells of the outer nuclear layer counted by Hoechst staining. The specificity and efficacy in mice were evaluated in a similar manner, using three retinas from two mice (Figure S10). Specificities and efficacies in the results section are displayed as mean ± sd.

#### Cell counting algorithms

To assess cone survival in organoids, an algorithm was designed that locates and counts local maxima in pixel intensity values corresponding to GFP-expressing cells. Three distinct counting approaches were employed: counting was done image-by-image from a 3D confocal stack (referred to as ‘3D-additive-count’), from the entire 3D stack (referred to as ‘3D-count’), and from the maximum intensity projection of the 3D stack (referred to as ‘MIP-count’).

Initially, Gaussian filtering was applied to each image to minimize background noise. Following this, local maxima in pixel intensity were identified within each image using the peak_local_max function from the skicit-image package in Python. Any detected local maxima that fell below 1.25 times the frame's average pixel value were disregarded. This was performed in 3D for the 3D-count. These detected local maxima were then subjected to a three-step filtering process to ensure they accurately represented cone cells.

In the first step of filtering, local maxima of low contrast were removed by applying Otsu thresholding to a local Region of Interest (ROI) around the local maximum. If active pixels were detected at the ROI edges, the window size was expanded. This iterative process continued until only inactive pixels were found at the ROI edges. Local maxima corresponding to ROIs exceeding a size of 70 x 70 pixels (113.75 x 113.75 μm) were excluded.

The second filtering step aimed to separate objects that were closely situated. Objects with a diameter ranging from 8.1 to 65 μm and with a perimeter-to-area ratio between 4 and 6.5 were selected for a process known as binary erosion, which effectively separated such adjacent or touching objects.

In the final filtering step, attributes like object diameter, perimeter-to-area ratio, and the contrast between object foreground and background were analyzed. Only objects with diameters between 6 and 100 μm and a perimeter-to-area ratio between 0.1 and 4 were retained. Low-contrast objects, defined as those for which the foreground was no more than 1.2 times brighter than the background, were also excluded.

All filtering steps for the 3D-count were done in 2D on the z-plane where each local maximum was identified.

In some experiments where noise levels were high, cell candidates where all pixels were below 150 were excluded (Figures 1H, 4G–4I, 7C, and S1). The cell counts obtained were normalized to the initial cell count yielding relative cone survival values.

For counting rod photoreceptors (Figures 5D–5F and 7D), slight modifications were made to the parameters of the cone-counting algorithm. For rod photoreceptors, the maximum intensity projections were quantified. The image resolution was enhanced fourfold via cubic interpolation. The minimum allowable diameter for cell candidates was also reduced from 4 μm to 2 μm, and any candidates where all pixels were below an intensity of 200 were excluded.

If not stated otherwise cone-survival was calculated using the counts from the 3D-additive-count algorithm.

#### Target categorization

Categorizer software^74^ was used to categorize the targets of the MOA compound library. Target categories were assigned to their respective compounds. If a compound had multiple targets, the most common category found among the targets was assigned.

#### Cell count thresholding

For the primary screen, thresholds were determined after visually inspecting images with the lowest reported D0 counts, ensuring the inclusion of as many data points as possible. These thresholds were set uniquely for each quantification algorithm (Figure S2). The thresholds for the secondary screens were the same as for the primary screen (Figures S4 and S6).

Similarly, thresholding on rod data was conducted following a visual inspection of images (Figure S10).

#### Adjusted cone survival

To compensate for the effect of initial cone counts on the survival of glucose deprivation, we calculated an adjusted version of the cone survival. For this, a linear model was fitted using the complete primary screen dataset to explain the cone survival with the logarithm of the scaled cone counts at D0. The obtained regression coefficient was then multiplied with the scaled logarithm of the D0 cone counts. Subtracting this term from the original cone survival yielded the adjusted values. This was done separately for all three cone counting algorithms (Figures 2E and S2).

This adjustment sometimes led to values higher than 100% and very rarely to values lower than 0%.

Since the cone-damaging secondary screen was done in normal glucose, we analyzed separately the relationship between cone survival and D0 counts in a newly calculated linear model. While we found a linear model that significantly explains this relationship, it only accounted for a marginal amount of the explained variation for all three quantification algorithms (n=711, R^2^=0.007-0.018, p-values<0.001, Figure S6). Therefore, we did not generate adjusted cone survival values for this dataset.

For the cone-saving secondary screen dataset, we used the linear model originally generated from the primary screen to compute the adjusted values. This primary screen model accurately predicted the D0 to cone survival relationship in the secondary screen (n=673-681, R^2^=0.17-0.22, p<0.001, Figure S6).

For all remaining low throughput experiments we used unadjusted cone survival values, because these experiments were performed on a different microscope. Therefore, the linear model of the primary screen did not accurately predict the D0 to cone survival relationship. In addition, the dataset for the low throughput experiments was too small to generate a new robust model.

#### Well position bias analysis

To account for any potential influence on the results of well position within the 96-well plate, an analysis was performed on the mean adjusted cone survival for each well position, based on the primary screen dataset with the 3D-additive-count. This procedure assumes that most of the compounds under study do not exert a significant effect on cone survival. These mean values were then compared. If the differences between the means were found to fall within the range of the minimum standard deviation observed for the least variable well, it was determined that the well position did not have a significant impact on the outcome. Excluding the positions of the normal glucose control wells, cone survival was not influenced by any of the well positions (Figure S2).

#### Analysis of primary screen data

To compare cone survival across various compound conditions while controlling for the initial count at D0, an Analysis of Covariance (ANCOVA) was conducted. In this analysis, the dependent variable was the unadjusted cone survival, and the independent variable was the compound condition (low glucose control vs. compound 1 vs. compound 2, etc.). The raw count at D0 served as the covariate in the model. P-values were calculated for a two-sided test comparing each compound to the low glucose controls. To identify significant hits from the primary screen, a statistical threshold of p<0.05 was set. The Benjamini-Hochberg correction was employed to account for multiple testing. Consequently, only compounds with an adjusted p-value less than 0.05 were considered significant. The compounds selected for secondary screening were determined based on their p-values and after visual inspection of images.

#### Mode of action names

The mode of action for each compound was sourced from Canham et al.^51^ For compounds subjected to secondary screens, a concise version of their modes of action was manually generated (Figures 3, 4, S4–S6, and S7).

#### Analysis of the secondary screen cone-damaging dataset

To assess the impact of different conditions compared to the control, an Analysis of Variance (ANOVA) was conducted that compared all different compound conditions to the normal glucose control. The resulting p-values were subsequently adjusted for multiple comparisons using Benjamini-Hochberg correction for all compounds and concentrations. P-values were calculated for a one-sided test. Compounds and concentrations with adjusted p-values less than 0.05 were deemed statistically significant. Controls are also displayed in Figure S4.

#### Clustering cone-damaging compounds from the secondary screen

To categorize compounds from the secondary screen based on cone survivals at four different concentrations, hierarchical clustering was performed using medians of the cone survival values of all significant cone-damaging compounds. The Elbow Method was employed to identify the optimal number of clusters, involving a plot of the total within-cluster sum of squares (WSS) against the number of potential clusters. WSS values were computed for all possible solutions, ranging from 1 to 34 clusters, and an elbow in the curve was observed at four clusters. The resulting clusters were then assigned to all compounds, as depicted in Figure 3C. One outlier was removed from cluster 3 and one from cluster 4 in Figure 3C.

#### Definition of target classes

HDAC1, HDAC2, HDAC3, HDAC4, HDAC5, HDAC6, HDAC7, HDAC8, HDAC9, HDAC10 and HDAC11 were categorized as HDAC I/IIs. SIRT1, SIRT2, SIRT3 and SIRT 6 were categorized as HDAC IIIs. TUBA1A, TUBA1B, TUBA1C, TUBA3C, TUBA3D, TUBA3E, TUBA4A, TUBA8, TUBB, TUBB1, TUBB2A, TUBB2B, TUBB3, TUBB4A, TUBB4B, TUBB6, TUBB8, TUBD1, TUBG1, and TUBG2 were categorized as tubulins. These target classes were used in Figures 3E, 3F, and S4.

#### Analysis of the secondary screen cone-saving dataset

The secondary screen cone-saving dataset was analyzed in the same way as the primary dataset using an ANCOVA with the D0 count as covariate, comparing compound effects to the low glucose controls. The resulting p-values were subsequently adjusted using a Benjamini-Hochberg correction for multiple testing for all compounds and concentrations (Figure S6). The p-values were calculated for a one-sided test. Controls are shown in Figure S6.

#### Analysis of target effects in the primary screen dataset

In the analysis of the impact of compound targets on cone survival, targets with three or more listed compounds were initially selected (Figure 2C). For each of these targets, the average of the median adjusted cone survival across all targeting compounds was determined. This average was then compared to a distribution generated by randomly drawing an equal number of compounds and calculating their mean of the median adjusted cone survival.

This process of random drawing was performed 10,000 times initially to create a distribution of mean values. In the analysis of cone-saving targets, the number of these randomly generated means that were higher than the observed mean was determined (Figure 4E and S8). Conversely, for the cone-damaging targets, the number that were lower was determined (Figure 3F).

If less than 10 of the random means were found to be higher (or lower, depending on the analysis), the process was repeated with 100,000 random draws to ensure robustness. The p-value for each target was then estimated as the fraction of random means that were found to be higher (or lower) than the observed mean, plus one, divided by the total number of random draws.

Finally, to account for multiple comparisons, these p-values were corrected using the Benjamini-Hochberg method and a significance threshold set at 0.05. However, in Figures 3F, 4E and S6 the unadjusted p-values are displayed.

#### Analysis of IC50

IC50 values for all compounds in the library targeting HSP90AA1, HSP90AB1, MTOR, PIK3CA, PIK3CB, PIK3CD, PDGFRA, and PDGFRB, were sourced from the ChEMBL database.^75^ This dataset encompassed reported IC50s, even for compound-target pairs not present in the MOA library. In instances where multiple IC50 values were noted for a specific compound-target combination, the median of these values was used for subsequent analysis. Spearman correlation coefficients were determined by correlating the median-adjusted cone survival with median IC50 values.

#### Analysis of transcriptomes

Sequencing reads were processed into gene counts using Snakemake (v7.21.0), a workflow management system. The workflow consisted of two main steps: read alignment and differential gene expression analysis. Reads were aligned against the GRCh38 (Ensembl release 109) reference genome using STAR (v2.7.10b). Both the number of reads per gene (--quantMode GeneCounts) and alignments translated into transcripts coordinates (--quantMode TranscriptomeSAM) were set as outputs. The reference genome was augmented to include sequences from two transgenes (ProA7-GFP and ProA330-tdTomato) used in cell sorting. Read counts per gene and per sample were aggregated using custom Python scripts, and genes expressed in fewer than 5% of the samples were filtered out. DESeq2 (v1.38) was then employed to identify differentially expressed genes, using a log_2_ fold change threshold of 1 and a 5% significance level after Benjamini-Hochberg correction for multiple hypothesis testing. Principal component analysis was performed using scikit-learn (v.1.2.2) on normalized and standardized gene counts. GSEApy pre-rank was employed to perform gene set enrichment analysis using gene ontology terms (biological process, molecular function and cellular component ontologies) as well as hallmark gene sets from the Human Molecular Signatures Database (MSigDB). For this purpose, genes were ranked based on Wald test statistics provided by DESeq2. Transcripts from ribosomal-protein coding genes were excluded from this analysis, as these genes can be variable or highly expressed irrespective of the tested conditions. Data were normalized to transcript counts per 10,000 adjusted for non-overlapping exon lengths (TP10k), where lengths were estimated using the R package GenomicFeatures (v1.50.2). Marker genes were identified based on an available adult human peripheral retina atlas (https://data.iob.ch) using scanpy’s rank_genes_groups function.

#### Analysis of kinase profiling

Kinase profiling data and the main analysis were provided by Reaction Biology Corporation. Gene expression values were derived from the average gene expression in low glucose cones.

#### Cell type-specific downregulation of target genes with shRNAs

DNA sequences targeting different regions of the target genes were designed using BLOCK-iT™ RNAi Designer online tool. The top three ranked sequences were then inserted into the miR-155 backbone. shRNAs were integrated in the AAV genome vector following the tdTomato coding sequence. The expression cassette was then inserted under the regulation of the cone specific promoter ProA7 for experiments in organoids, or under EF1a constitutive promoter for validations in HEK293T cells. A scrambled version of the shRNAs targeting one of the genes was used as control.

We validated the effect of the designed shRNAs in downregulating the expression of target genes in adherent HEK293T cells. Cells were seeded in 6-well plates with DMEM (Gibco, #10566016) supplemented with 10% fetal bovine serum and 1% Penicillin-Streptomycin (Gibco, #15140-122). Then, we transfected them with a mix of two plasmids (i) a plasmid that overexpressed the coding sequence of the targeted gene downstream of the constitutive CMV promoter together with GFP as a control of successful transfection, and (ii) the plasmid containing tdTomato and the three shRNAs regulated by the constitutive promoter EF1a (Figure S22). Plasmids were mixed at a 1:3 ratio (0.5 ug plasmid (i) and 1.5 ug of plasmid (ii)) with PEIMAX (Polyscience, #POL24765-1) and DMEM, incubated 15 mins and then added to the cells. 72 hours later we collected the cells, extracted RNA with RNeasy Plus Mini Kit (Qiagen, #74134) and performed reverse transcription with High-Capacity cDNA Reverse Transcription Kit (Applied Biosystems, #4368814). The expression levels of the target genes in cells transfected with gene-specific shRNAs were compared to the ones transfected with scrambled shRNAs with RT-qPCR (Applied Biosystems, QuantStudio™ 3 Real-Time PCR System) using FastStart Universal SYBR Green Master (Rox) (Roche, #FSUSGMMRO) and corresponding gene specific primers and GAPDH as a reference housekeeping gene (see Table S6). Then, we generated AAVs including shRNAs that showed a significant reduction of gene expression levels in HEK293T cells and transduced them into retinal organoids at week 26. These organoids were glucose starved at week 30 and live imaged at D0 and D7 after glucose removal from the medium. AAV transduction, glucose starvation and cell counting were performed as described previously.

#### H_2_O_2_ experiments

AAV transduction with ProA7-GFP transgene was performed as described in previous sections. H_2_O_2_ was added 4 weeks post transduction (Sigma, #216763). First, a concentration series of H_2_O_2_ was tested to find the concentration that resembled the cone survival curve after low glucose starvation the most. Automated imaging was done on the day of addition and on D1,2,3,4 and 7. For compound validation, the optimal concentration, 50 μM H_2_O_2_, was added 4 weeks after transduction together with the compounds at two concentrations. Automated imaging was done on the day of addition and 7 days later.

#### Retinal injections of compounds in rd10 mice

Rd10 mice were kept in dark-rearing conditions at all times. We injected at P14, P21, P28 and P35. Injections were performed under dim red light. We tested CS-KI-1, CS-KI-2, CK1-I-1, CK1-I-2 and CK1-I-3, at two different concentrations. In the right eye, we injected the compound reconstituted in DMSO and then diluted in PBS at 10 μM or 100 μM. We used the left eye as a control, in which we injected a DMSO vehicle control diluted in PBS. We diluted the same volume of 100% DMSO as used for the compound into PBS. Thus for compounds injected at 10 μM, we injected 0.1% DMSO in PBS in the control eye, and for compounds injected at 100 μM, we injected 1% DMSO in the control eye. Mice were anesthetized with isoflurane and then 1 μL of vehicle or compound were injected intravitreally using a nanoinjector (Nanoliter 2020, WPI). Treated mice were given carprofen (50 mg/mL, Rimadyl) 24 h before the surgery for up to 48 h. The injected eye was post-treated with Novesin 0.4% (OmniVision).

#### Optical Coherence Tomography (OCT) and data analysis

OCT recordings were made on an Envisu R2210 device from Bioptigen/Leica using a custom objective adapted for mouse eyes. Mice were imaged in the dark under dim red light at two time points: P27-28 and P42-43. The animals were anaesthetised with a combination of Fentanyl (0.05 mg/kg), Midazolam (5 mg/kg), and Medetomidine (0.5 mg/kg) (FMM) and were adequately warmed for the duration of the imaging process. Their pupils were dilated using tropicamide eye drops and their eyes were moisturised before and after image acquisition. After the measurements, mice received a reversal cocktail of Flumazenil (0.5 mg/kg) and Atipamezol (2.5 mg/kg). The acquired images were volume scans spanning the majority of the retina (Area: 1.4 x 1.4 mm^2^, A-Scans/B-Scan: 1000, B-Scans: 100, Averaging: 10x). The retina thickness was measured as the distance from the RPE to the inner retinal surface, and in the case of the ONL, the thickness was measured as the distance from the outer limiting membrane to the outer boundary of the outer plexiform layer. The reported thickness values are the average of the retina thickness values at the midpoint between the optic nerve and the temporal or nasal edge of the retina. To calculate the percentage of thickness conservation, we divided the thickness measured at P42 by the thickness measured at P27 (Figures 7H–7J and S25). Each data point represents one retina. For the statistical comparisons, the percentage of retinal or ONL thickness conserved of the eye injected with the compound was compared with the eye injected with vehicle from the same mouse, using one-tailed paired t tests for all the mice injected with the same compound. In two mice the control eyes already denegerated at P27, they were below the 95 percentile of retinal thickness distribution of control eyes (potentially due to injection-related damage or unintended light exposure). These two mice were excluded from the analysis.

## Supplemental information

Document S1. Figures S1–S25 and Table S6

Table S1. Compound summary of all significant cone-damaging compounds of the primary screen, related to Figures 2, 3, and S3

Table S2. Compound summary of all retested cone-damaging compounds in the secondary screen, related to Figures 3, S4, and S5

Table S3. Compound summary of all retested cone-saving compounds in the secondary screen, related to Figures 4, S6, and S7

Table S4. Kinase profiling results, related to Figure 7

Table S5. Summary of CK-1 and MAPK11 inhibitors, related to Figure 7

Table S7. Kinase profiling assay conditions, related to Figure 7 and STAR Methods

Document S2. Article plus supplemental information
