# Supplementary material for: Cell-type-focused compound screen in human organoids reveals CK1 inhibition protects cone photoreceptors from death
Source: Neuron. 2026 Jul 1;114(13):2347–2366.e12. doi: 10.1016/j.neuron.2026.02.024 (PMC13353051; doi:10.1016/j.neuron.2026.02.024)
Supplement: Document S1. Figures S1–S25 and Table S6 [file mmc1.pdf]

Neuron, Volume *114*

## Supplemental information

# Cell-type-focused compound screen in human organoids reveals CK1 inhibition protects cone photoreceptors from death

**Stefan E. Spirig, Álvaro Herrero-Navarro, Larissa Utz, Valeria J. Arteaga-Moreta, Zoltan Raics, Susana Posada-Céspedes, Stephanie Chreng, Olaf Galuba, Inga Galuba, Isabelle Claerr, Steffen Renner, Miklos Boldogkoi, Verónica Moreno-Juan, P. Timo Kleindienst, Adrienn Volak, Jannick Imbach, Svitlana Malysheva, Rebecca A. Siwicki, Vincent Hahaut, Yanyan Hou, Tiago M. Rodrigues, Simone Picelli, Marco Cattaneo, Josephine Jüttner, Cameron S. Cowan, Myriam Duckely, Daniel K. Baeschlin, Magdalena Renner, Vincent Unterreiner, and Botond Roska**

## Supplemental figures

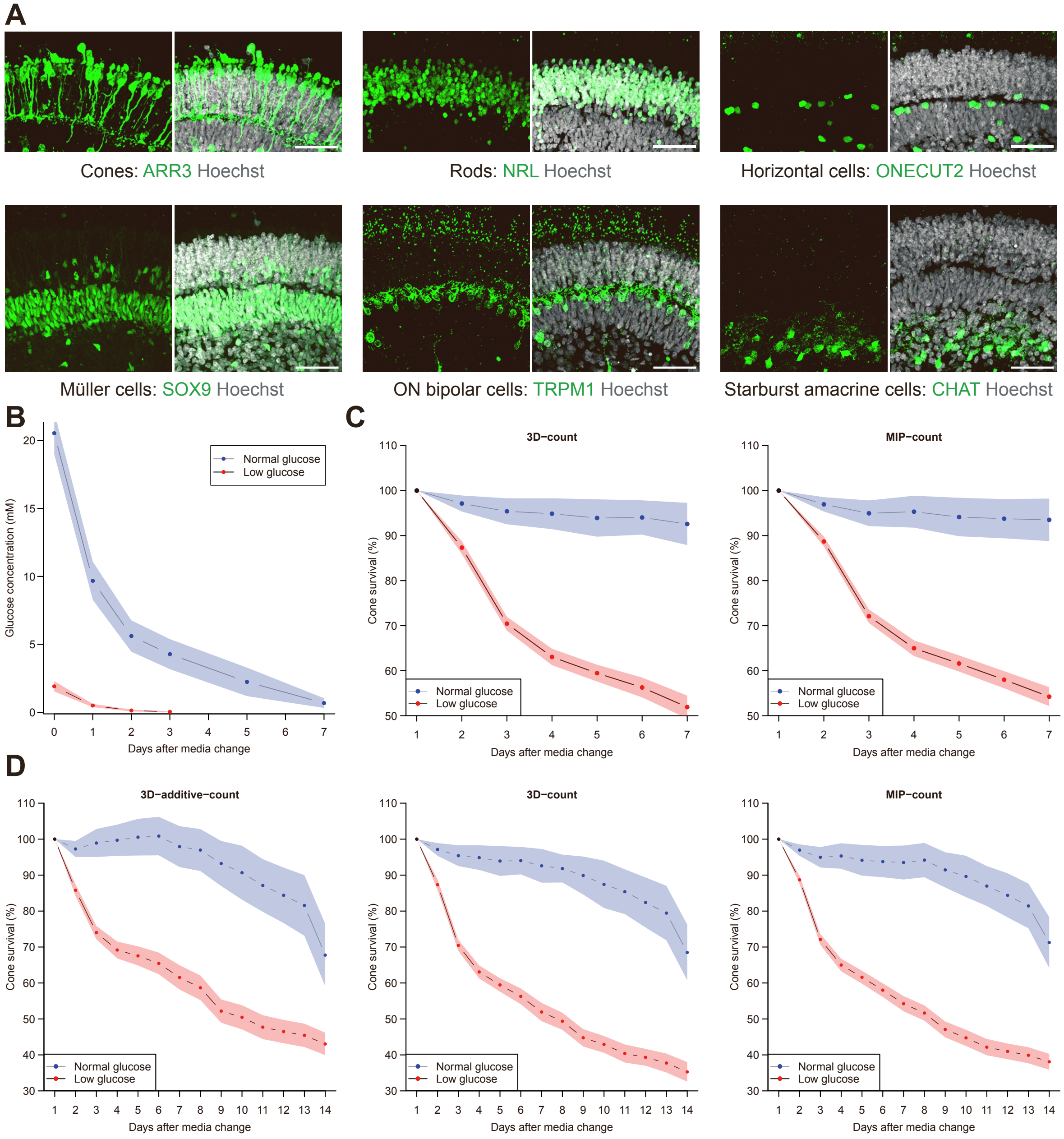


**Figure S1. Cell types of mature human retinal organoids and glucose starvation, related to Figure 1.**

**A:** Marker gene expression of cell types in human retinal organoids. Confocal images of sectioned and stained human retinal organoids at week 30 of maturation with indicated staining (scale bar, 25 µm). **B:** Glucose consumption assay of human retinal organoids. Mean ± se from 10 human retinal organoids. **C:** Quantification of cone survival in human retinal organoids in normal and low glucose over seven days. Quantification with either 3D-count (left) or MIP-count (right). Mean ± se. Low glucose, n=26; normal

glucose, n=6. **D:** Same as (C) but for 14 days using all three quantification algorithms (3D-additive-count, 3D-count, and MIP-count).

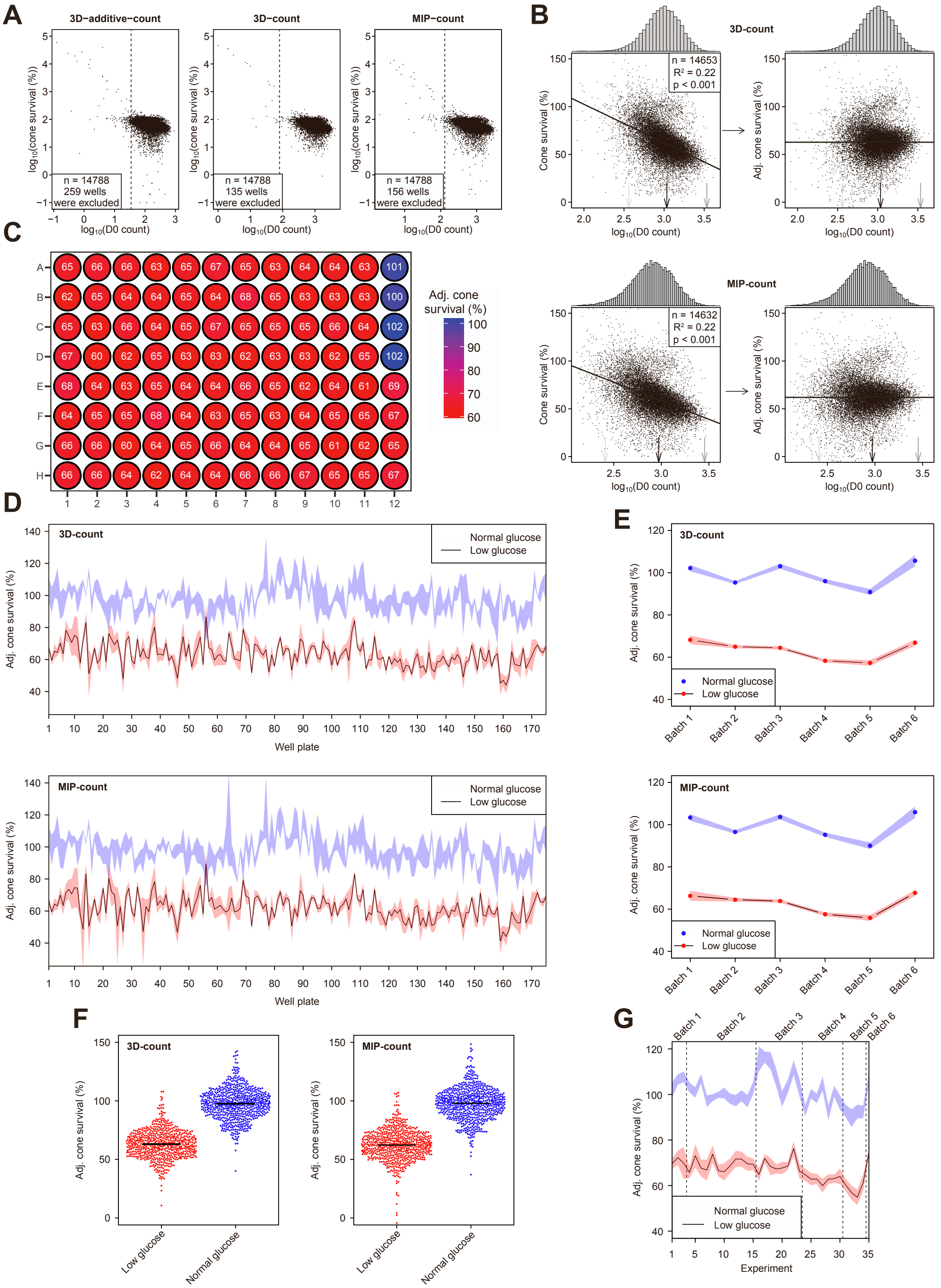
A
3D−additive−count
3D−count
MIP−count
log10(cone survival (%))
log10(D0 count)
n = 14788
259 wells
were excluded
n = 14788
135 wells
were excluded
n = 14788
156 wells
were excluded
B
3D-count
MIP-count
n = 14653
R² = 0.22
p < 0.001
n = 14632
R² = 0.22
p < 0.001
Cone survival (%)
Adj. cone survival (%)
log10(D0 count)
C
Adj. cone
survival (%)
D
3D-count
MIP-count
Normal glucose
Low glucose
Adj. cone survival (%)
Well plate
E
3D-count
MIP-count
Normal glucose
Low glucose
Batch 1
Batch 2
Batch 3
Batch 4
Batch 5
Batch 6
F
3D-count
MIP-count
Low glucose
Normal glucose
G
Batch 1
Batch 2
Batch 3
Batch 4
Batch 5
Batch 6
Normal glucose
Low glucose
Experiment

**Figure S2: Primary screen: analysis, related to Figure 2.**

**A:** Data thresholding based on counts at D0 for all quantification algorithms. The dotted red line indicates the threshold beyond which wells are included in the analysis. **B:** Adjustment of cone survival values based on the D0 count. Top: 3D-count. Bottom: MIP-count. Left: cone survival and the logarithm of the cone count at D0, fitted with a linear model. Regression line, red; summary statistics, top right corner of each panel. Right: adjusted cone survival and the logarithm of the cone count at D0 with the transformed regression line. Colored arrows correspond to D0 counts of example images in Figure 2E. **C:** Well position bias analysis. The means of adjusted cone survival for all wells of the primary screen data are depicted. **D-E:** Adjusted cone survival of normal- and low-glucose controls for individual well plates (D) and organoid batches (E), mean ± se. Top: 3D-count. Bottom: MIP-count. **F:** Adjusted cone survival of all normal- and low-glucose controls. Left: 3D-count. Right: MIP-count. **G:** Adjusted cone survival of normal- and low-glucose controls for individual experiments (groups of 5 well plates containing all replicates for compounds) using the 3D-additive-count algorithm, mean ± se.

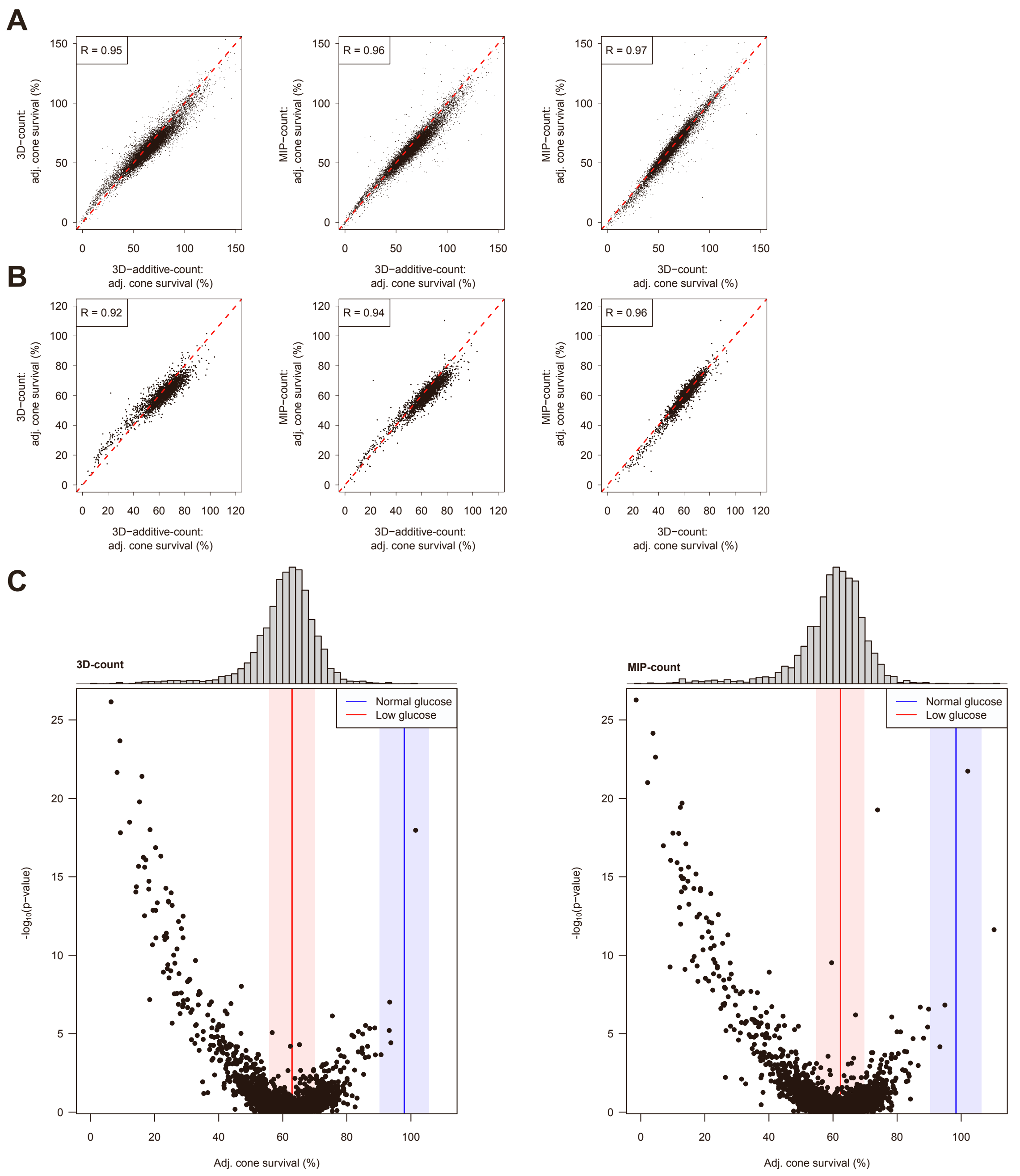


**Figure S3: Primary screen: comparison of different quantifications, related to Figure 2.**

**A-B:** Comparison of different quantification methods. Comparing either adjusted cone survival values of individual organoids (A) or the median of adjusted cone survival values for individual compounds (B). The dotted red line represents the unity line and ‘R’ denotes the Pearson’s correlation coefficient. **C:** Effect of compounds on cone survival in the primary screen. Left: 3D-count. Right: MIP-count. Each dot corresponds to the effect of one compound, with the median of the adjusted cone survival of five human retinal organoids on the x-axis and the p-value comparing the cone survival in the compound and in low glucose on the y-

axis. The median (line) and the interquartile range (shaded area) of normal (blue) and low (red) glucose controls are indicated. Top: the distribution of median adjusted cone survival for the compounds.

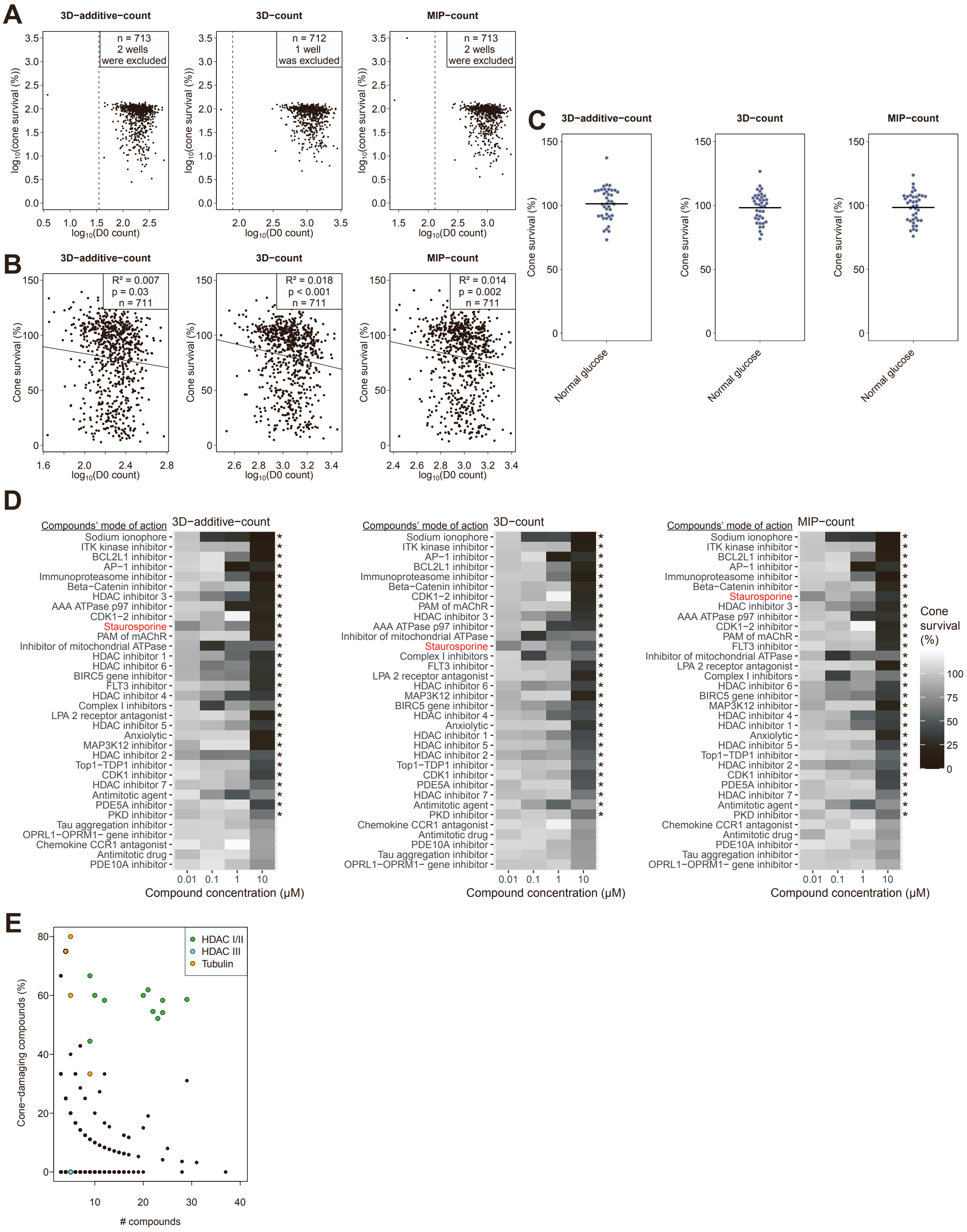

A
3D−additive−count
3D−count
MIP−count
n = 713
2 wells
were excluded
n = 712
1 well
was excluded
n = 713
2 wells
were excluded
log10(cone survival (%))
log10(D0 count)
B
R² = 0.007
p = 0.03
n = 711
R² = 0.018
p < 0.001
n = 711
R² = 0.014
p = 0.002
n = 711
Cone survival (%)
log10(D0 count)
C
3D−additive−count
3D−count
MIP−count
Cone survival (%)
Normal glucose
D
Compounds' mode of action
3D−additive−count
Sodium ionophore
ITK kinase inhibitor
BCL2L1 inhibitor
AP−1 inhibitor
Immunoproteasome inhibitor
Beta−Catenin inhibitor
HDAC inhibitor 3
AAA ATPase p97 inhibitor
CDK1−2 inhibitor
Staurosporine
PAM of mAChR
Inhibitor of mitochondrial ATPase
HDAC inhibitor 1
HDAC inhibitor 6
BIRC5 gene inhibitor
FLT3 inhibitor
HDAC inhibitor 4
Complex I inhibitors
LPA 2 receptor antagonist
HDAC inhibitor 5
Anxiolytic
MAP3K12 inhibitor
HDAC inhibitor 2
Top1−TDP1 inhibitor
CDK1 inhibitor
HDAC inhibitor 7
Antimitotic agent
PDE5A inhibitor
PKD inhibitor
Tau aggregation inhibitor
OPRL1−OPRM1− gene inhibitor
Chemokine CCR1 antagonist
Antimitotic drug
PDE10A inhibitor
Compound concentration (µM)
Compounds' mode of action
3D−count
Sodium ionophore
ITK kinase inhibitor
AP−1 inhibitor
BCL2L1 inhibitor
Immunoproteasome inhibitor
Beta−Catenin inhibitor
CDK1−2 inhibitor
PAM of mAChR
HDAC inhibitor 3
AAA ATPase p97 inhibitor
Inhibitor of mitochondrial ATPase
Staurosporine
Complex I inhibitors
FLT3 inhibitor
LPA 2 receptor antagonist
HDAC inhibitor 6
MAP3K12 inhibitor
BIRC5 gene inhibitor
HDAC inhibitor 4
Anxiolytic
HDAC inhibitor 1
HDAC inhibitor 5
HDAC inhibitor 2
Top1−TDP1 inhibitor
CDK1 inhibitor
PDE5A inhibitor
HDAC inhibitor 7
Antimitotic agent
PKD inhibitor
Chemokine CCR1 antagonist
Antimitotic drug
PDE10A inhibitor
Tau aggregation inhibitor
OPRL1−OPRM1− gene inhibitor
Compound concentration (µM)
Compounds' mode of action
MIP−count
Sodium ionophore
ITK kinase inhibitor
BCL2L1 inhibitor
AP−1 inhibitor
Immunoproteasome inhibitor
Beta−Catenin inhibitor
Staurosporine
HDAC inhibitor 3
AAA ATPase p97 inhibitor
CDK1−2 inhibitor
PAM of mAChR
FLT3 inhibitor
Inhibitor of mitochondrial ATPase
LPA 2 receptor antagonist
Complex I inhibitors
HDAC inhibitor 6
BIRC5 gene inhibitor
MAP3K12 inhibitor
HDAC inhibitor 4
HDAC inhibitor 1
Anxiolytic
HDAC inhibitor 5
Top1−TDP1 inhibitor
HDAC inhibitor 2
CDK1 inhibitor
PDE5A inhibitor
HDAC inhibitor 7
Antimitotic agent
PKD inhibitor
Chemokine CCR1 antagonist
Antimitotic drug
PDE10A inhibitor
Tau aggregation inhibitor
OPRL1−OPRM1− gene inhibitor
Compound concentration (µM)
Cone survival (%)
E
HDAC I/II
HDAC III
Tubulin
Cone−damaging compounds (%)
# compounds

**Figure S4: Secondary screen for cone-damaging compounds: analysis, related to Figure 3.**

**A:** Thresholding of data for the secondary screen of cone-damaging compounds based on counts at D0 for all quantification methods. The dotted red line indicates the threshold beyond which wells are included in the analysis. **B:** Effect of D0 count. Cone survival and the logarithm of the cone count at D0, fitted with a linear model. The regression line is shown in red and the summary statistics are displayed at top right. **C:** Normal-glucose controls with indicated means for all three quantification methods. **D:** Summary of the effects of all cone-damaging compounds tested in the secondary screen. The positive control Staurosporine is indicated in red. Compounds are sorted by the minimum p-value across all concentrations, with the smallest p-value at the top. Significant compounds (at any concentration) are marked with *, denoting a p-value<0.05 (after Benjamini Hochberg correction for multiple testing). **E:** Target analysis showing the number of compounds against the fraction of compounds having a significant cone-damaging effect (p-values<0.05, after Benjamini Hochberg correction for multiple testing) for all targets with more than two compounds.

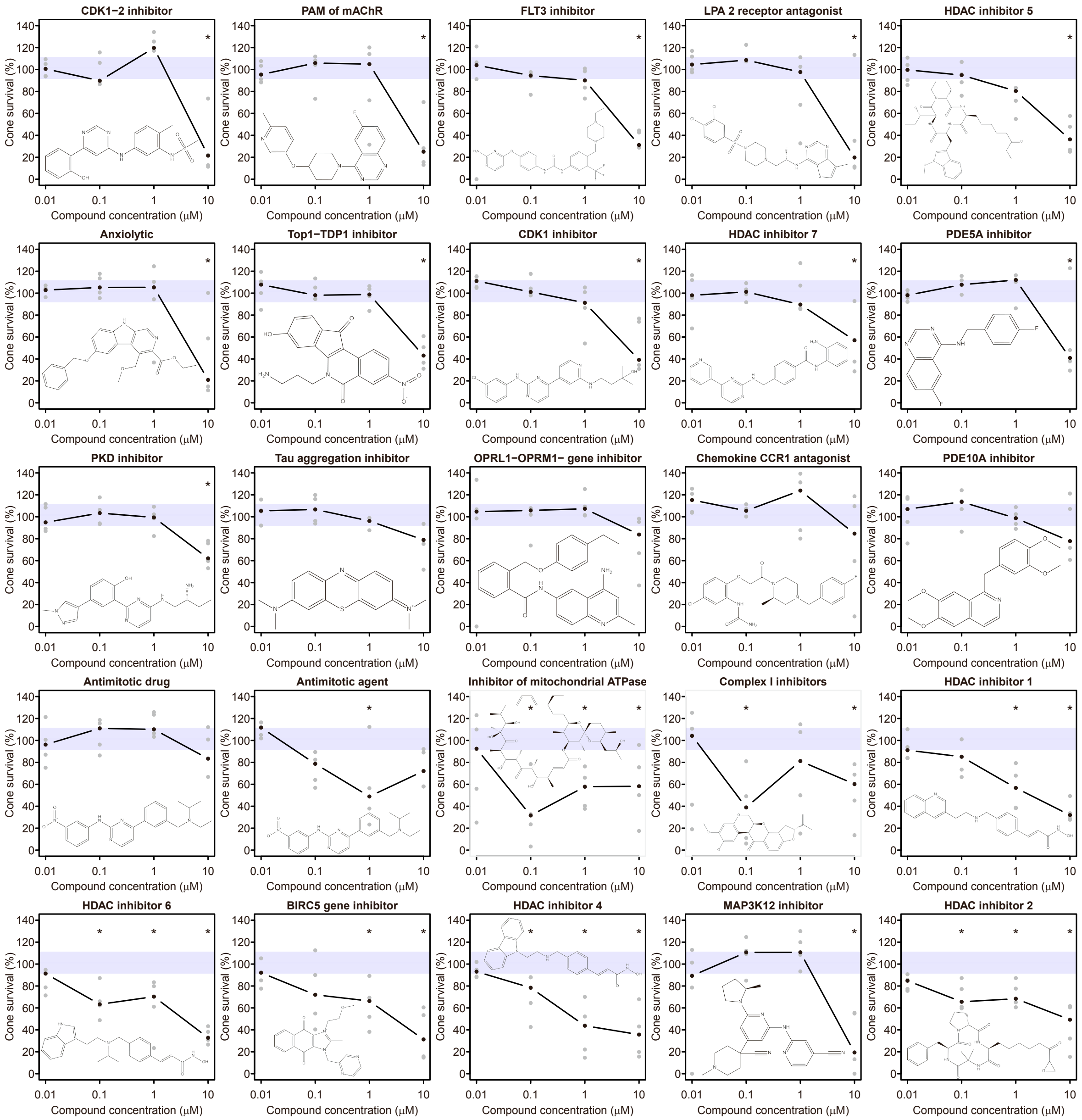


**Figure S5: Secondary screen for cone-damaging compounds: dose-response curves, related to Figure 3.**

Dose-response curves of all secondary screen cone-damaging compounds, excluding those depicted in Figure 3D. The compounds are ordered based on their respective clusters from Figure 3D and minimum p-values. Black dots denote median cone survival, gray dots represent individual values. The blue line and area indicate median and interquartile range of the normal-glucose controls, respectively. The colored frame corresponds to the clusters from Figure 3C. The structure of each compound is illustrated within the plot.

Significant compounds and concentrations are marked with * denoting a p-value<0.05 (after Benjamini Hochberg correction for multiple testing).

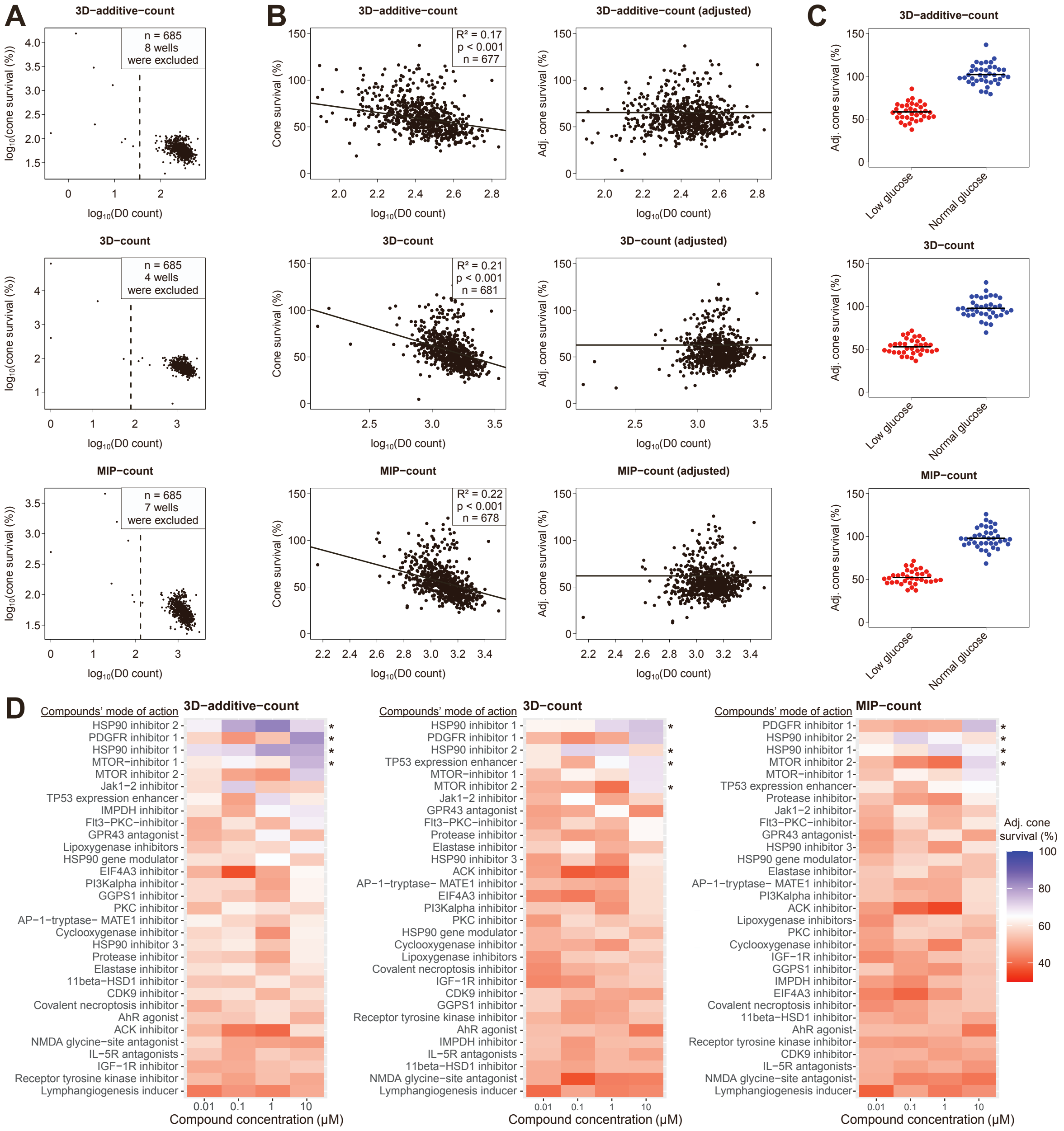


**Figure S6: Secondary screen for cone-saving compounds: analysis, related to Figure 4.**

**A:** Thresholding of data for the secondary screen of cone-saving compounds based on counts at D0 for all quantification methods. The dotted red line indicates the threshold beyond which wells are included in the analysis. **B:** Adjustment of cone survival values based on D0 counts for all quantification methods. Left: cone survival and the logarithm of the cone counts at D0, fitted with a linear model. Regression line, red; summary statistics, top right corner of each panel. Right: adjusted cone survival and the logarithm of the

cone counts at D0 with the transformed regression line. **C:** Adjusted cone survival of all normal- and low-glucose controls for all quantification methods. **D:** Summary the effects of cone-saving compounds. Compounds are sorted by the maximum median adjusted cone survival. Results are shown for all three quantification methods. Significant compounds are marked with *, denoting a p-value<0.05 (after Benjamini Hochberg correction for multiple testing).

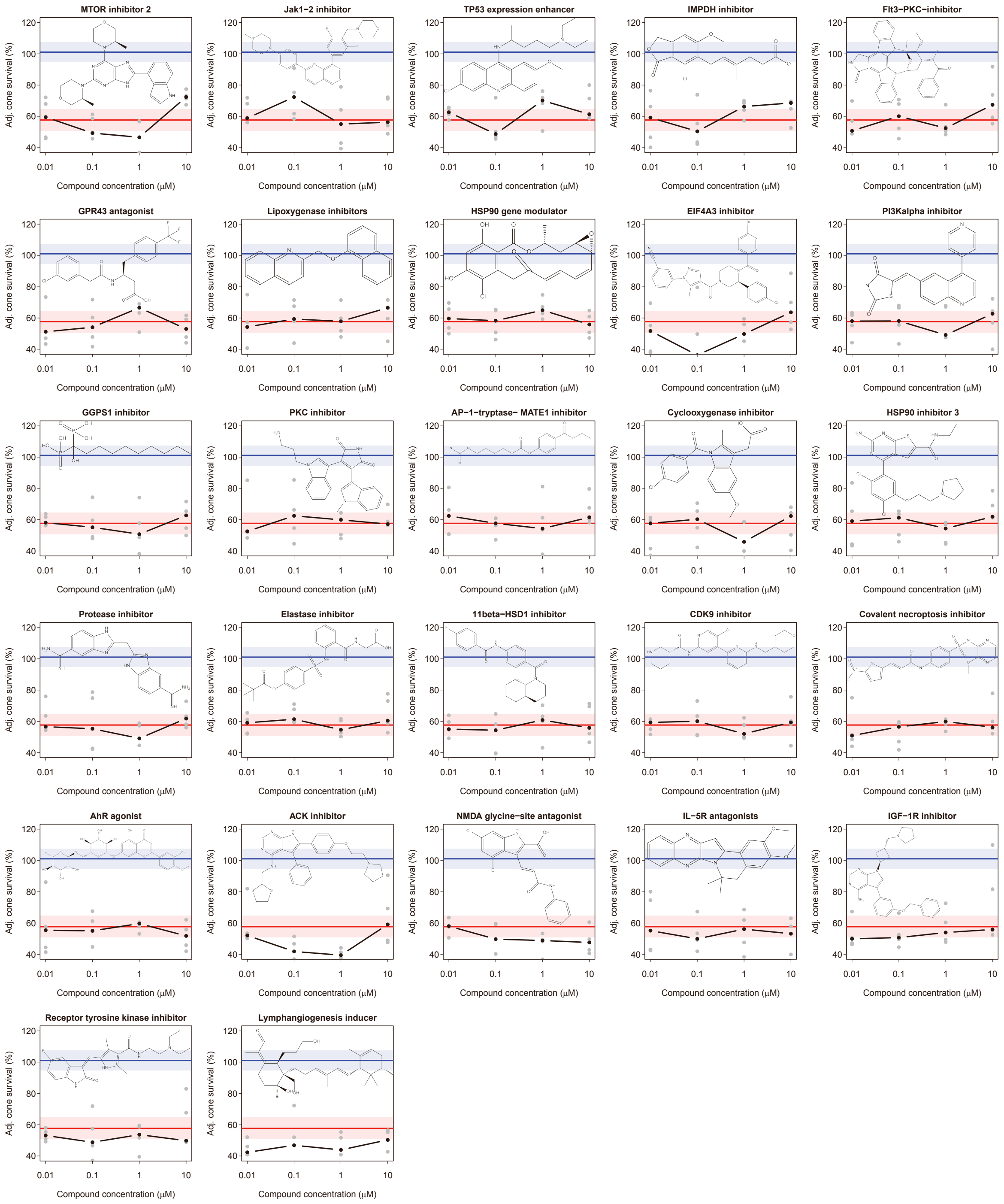
MTOR inhibitor 2
Jak1−2 inhibitor
TP53 expression enhancer
IMPDH inhibitor
Flt3−PKC−inhibitor
GPR43 antagonist
Lipoxygenase inhibitors
HSP90 gene modulator
EIF4A3 inhibitor
PI3Kalpha inhibitor
GGPS1 inhibitor
PKC inhibitor
AP−1−tryptase− MATE1 inhibitor
Cyclooxygenase inhibitor
HSP90 inhibitor 3
Protease inhibitor
Elastase inhibitor
11beta−HSD1 inhibitor
CDK9 inhibitor
Covalent necroptosis inhibitor
AhR agonist
ACK inhibitor
NMDA glycine−site antagonist
IL−5R antagonists
IGF−1R inhibitor
Receptor tyrosine kinase inhibitor
Lymphangiogenesis inducer
Adj. cone survival (%)
Compound concentration (μM)
40
60
80
100
120
0.01
0.1
1
10

**Figure S7: Secondary screen for cone-saving compounds: dose-response curves, related to Figure 4.**

Dose-response curves of all secondary screen cone-saving compounds, excluding those depicted in Figure 4B. Compounds are ordered by their maximum adjusted cone survival (most effective concentration). Black dots denote median cone survival, gray dots represent individual values. The median (line) and the interquartile range (shaded area) of normal (blue) and low (red) glucose controls are indicated. The structure of each compound is illustrated within the plot.

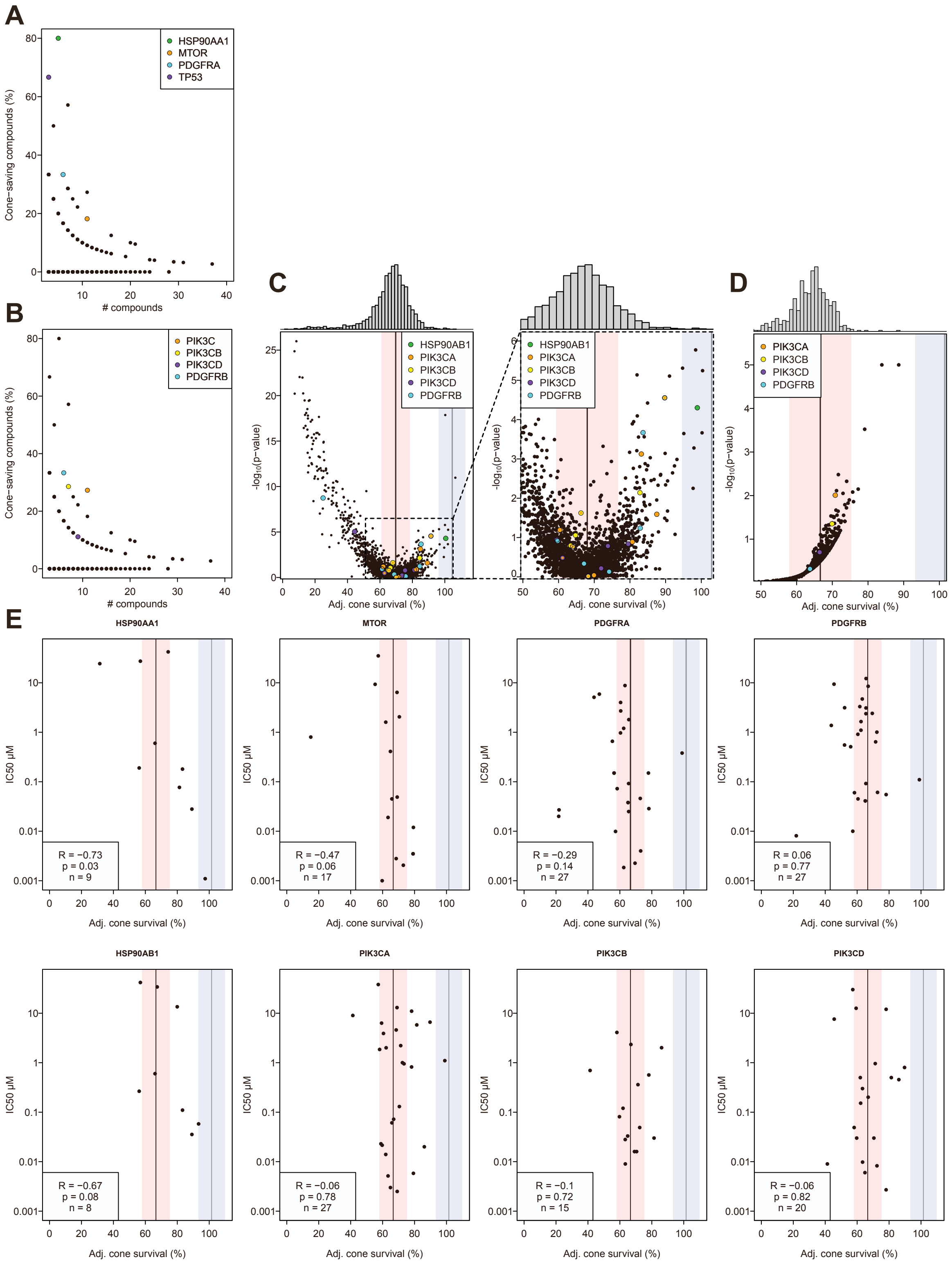
A
HSP90AA1
MTOR
PDGFRA
TP53
Cone−saving compounds (%)
# compounds
B
PIK3C
PIK3CB
PIK3CD
PDGFRB
Cone−saving compounds (%)
# compounds
C
HSP90AB1
PIK3CA
PIK3CB
PIK3CD
PDGFRB
-log10(p−value)
Adj. cone survival (%)
D
PIK3CA
PIK3CB
PIK3CD
PDGFRB
-log10(p−value)
Adj. cone survival (%)
E
HSP90AA1
R = −0.73
p = 0.03
n = 9
MTOR
R = −0.47
p = 0.06
n = 17
PDGFRA
R = −0.29
p = 0.14
n = 27
PDGFRB
R = 0.06
p = 0.77
n = 27
HSP90AB1
R = −0.67
p = 0.08
n = 8
PIK3CA
R = −0.06
p = 0.78
n = 27
PIK3CB
R = −0.1
p = 0.72
n = 15
PIK3CD
R = −0.06
p = 0.82
n = 20
IC50 µM
Adj. cone survival (%)

**Figure S8: Target analysis and IC50s, related to Figure 4.**

**A-B:** Number of compounds and fractions of compounds having a median adjusted cone survival >80% for all targets (with more than two compounds). Indicated targets colored. **C:** Left: adjusted cone survival and p-values in the primary screen. Compound targets are indicated and colored. The median (line) and the interquartile range (shaded area) of normal (blue) and low (red) glucose controls are indicated. Right: zoom-in of plot on the left. **D:** Target analysis of primary screen. The means of the median adjusted cone survival for each target are shown, along with their p-values. Specific targets are indicated. The median and the interquartile range are labeled as in (C). **E:** Median IC50 values for compounds that bind the annotated targets (labeled on top) of cone-saving compounds and their median adjusted cone survival. The median and the interquartile range are labeled as in (C).

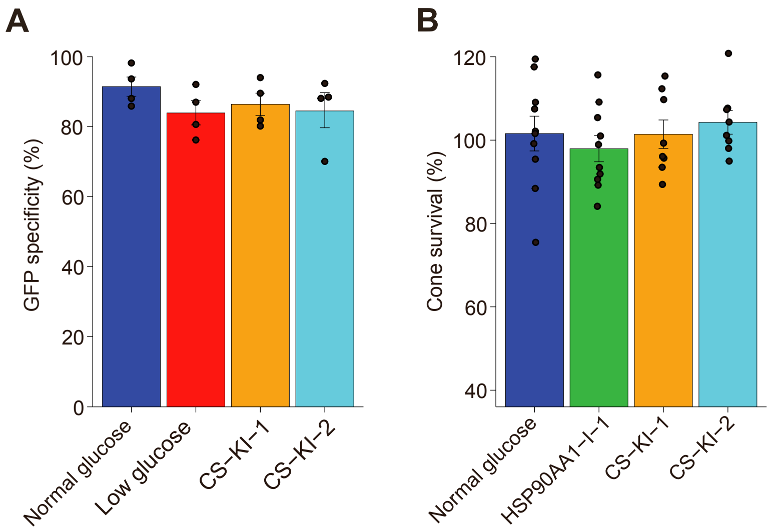


**Figure S9: Compound effects in normal glucose and on GFP expression, related to Figure 4.**

**A:** Quantification of the specificity of GFP expression in cones in organoids treated with normal glucose, low glucose, CS-KI-1 and CS-KI-2 compounds. **B:** Cone survival of compound-treated organoids in normal glucose after seven days. HSP90AA1-I-1 was administered at 1 µM, while CS-KI-1 and CS-KI2 were administered at 10 µM. Results are shown as mean ± se. Black dots indicate individual organoids.

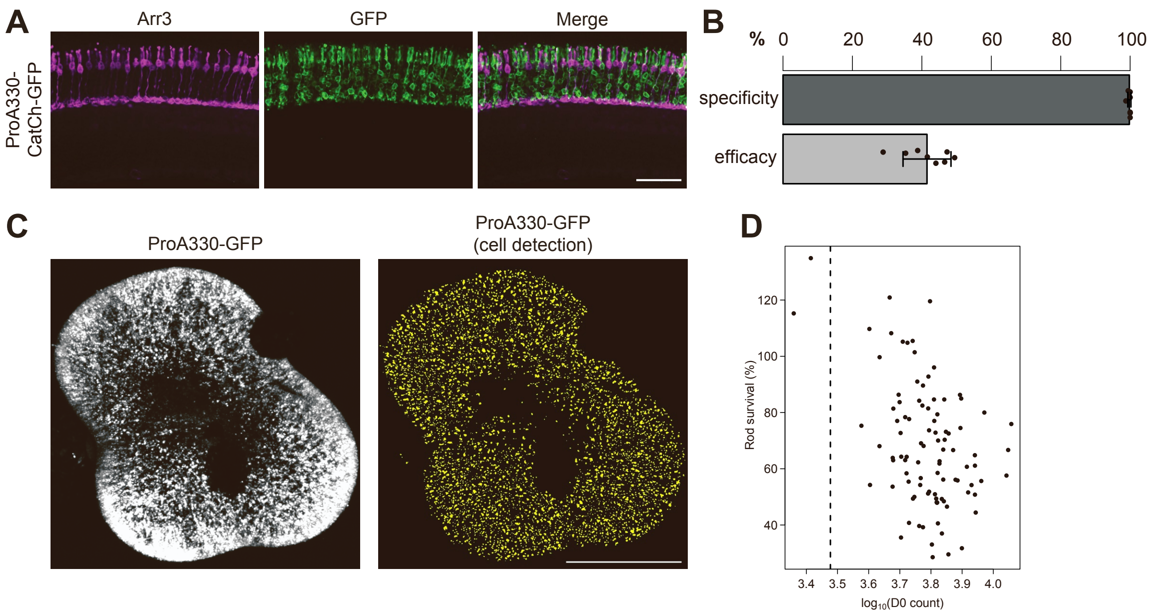


**Figure S10: Rod promoter, related to Figure 5.**

**A:** Confocal image of sectioned and stained transduced mouse retina (scale bar, 50 µm). Cone marker Arr3, magenta; CatCh-GFP, green. **B:** Quantification of the specificity and efficacy of rod labeling in mice by ProA330-CatCh-GFP AAV. Rods were identified as being present in the photoreceptor layer but negative for the cone-marker Arr3. Results are shown as mean ± sd. **C:** Left: example image of a ProA330-GFP AAV-transduced human retinal organoid. GFP, white. Right: Detected rods of an example human retinal organoid. Detected cells, yellow. **D:** Data thresholding based on rod counts at D0. The dotted red line indicates the threshold beyond which wells are included in the analysis.

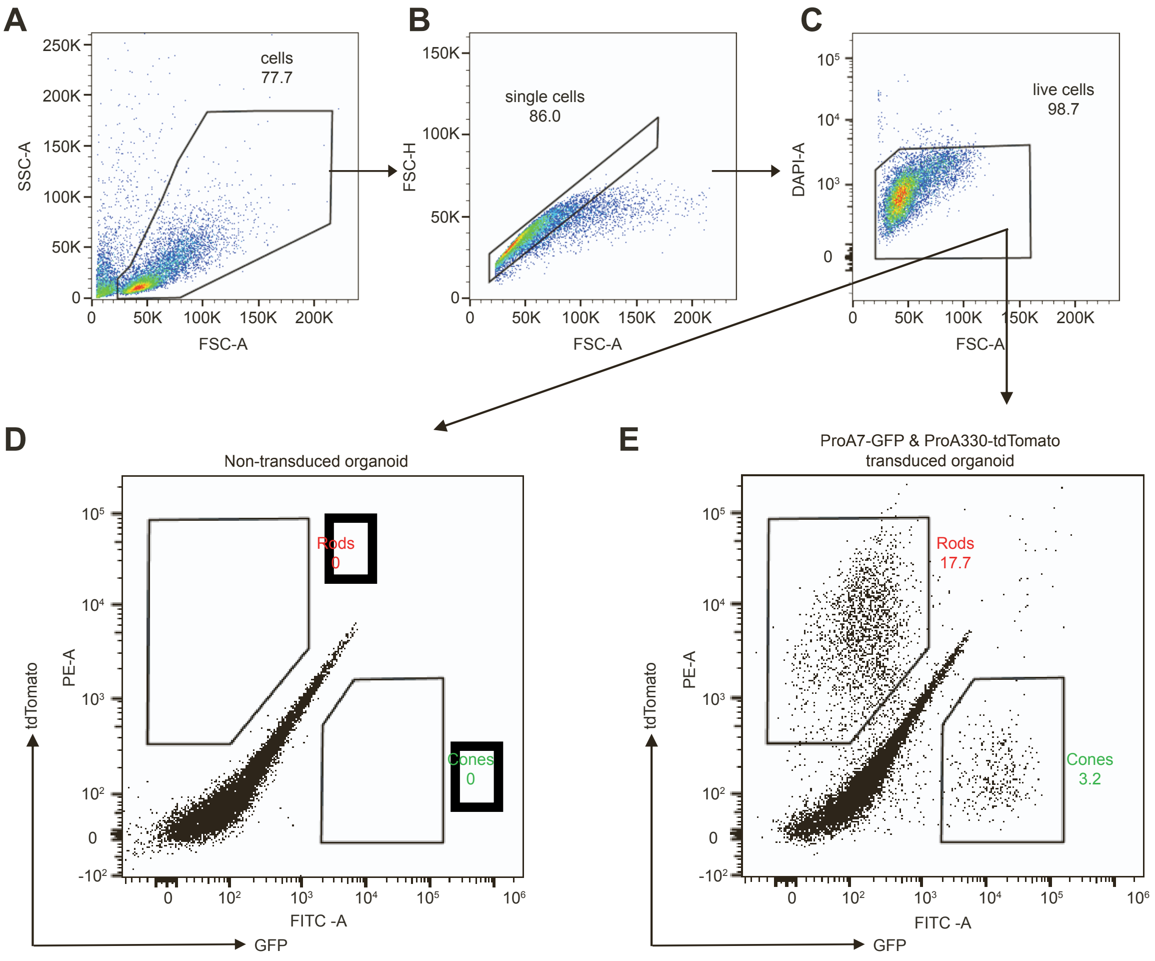


**Figure S11: Fluorescence activated cell sorting (FACS) of photoreceptors, related to Figure 6.** Representative FACS density plots from a human retinal organoid co-transduced with ProA7-GFP and ProA330-tdTomato. All numbers are percentages of gated cells. **A:** Forward scatter area (FSC-A) and side scatter area (SCS-A) to filter cells from debris. **B:** FSC-A and forward scatter height (FSC-H) to filter single cells form aggregates. **C:** FSC-A and Hoechst channel (DAPI-A) to sort out living cells. **D-E:** GFP (cone) and tdTomato (rod) positive cells from either non-transduced (D) or co-transduced (E) human retinal organoids.

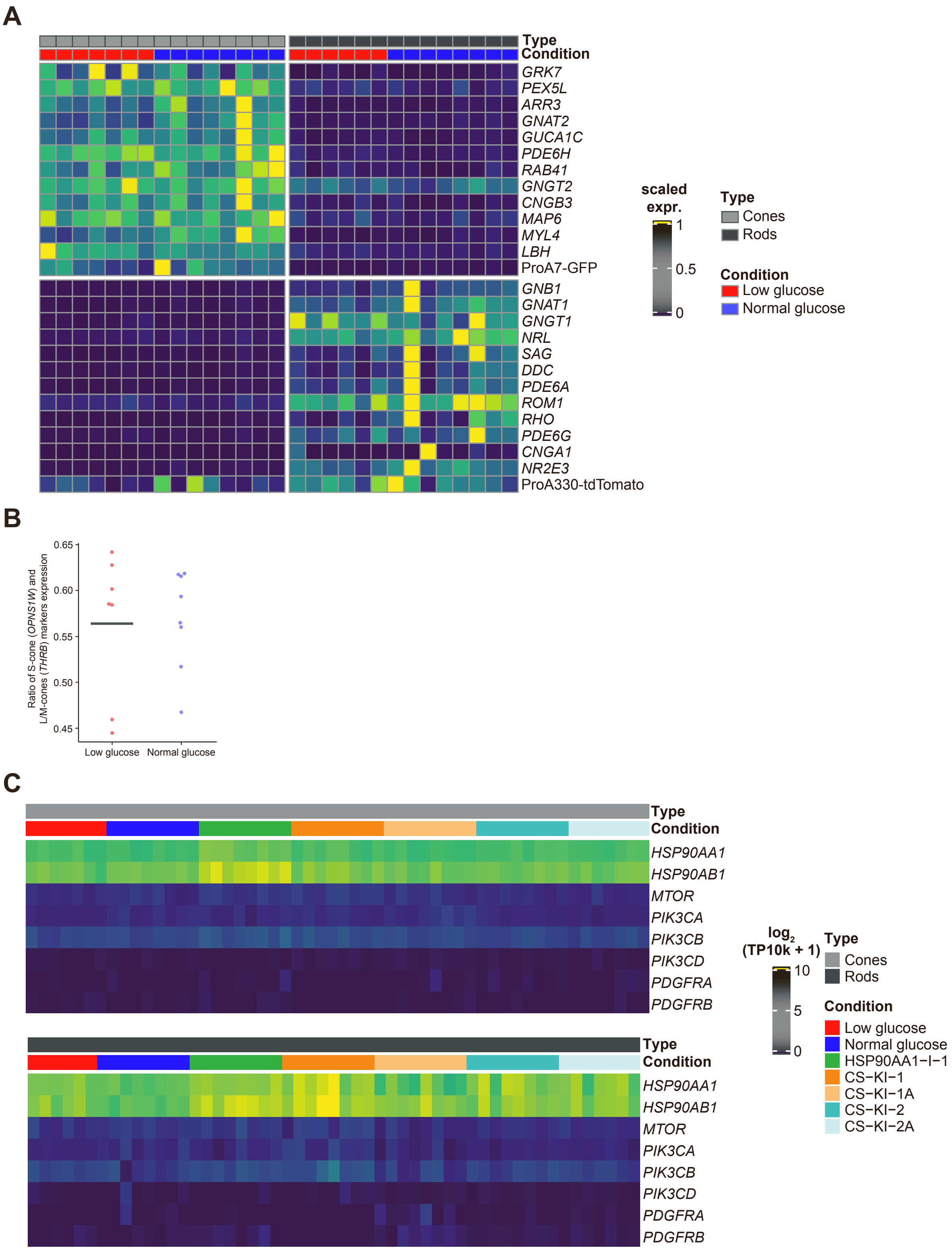

A
Type
Condition
GRK7
PEX5L
ARR3
GNAT2
GUCA1C
PDE6H
RAB41
GNGT2
CNGB3
MAP6
MYL4
LBH
ProA7-GFP
GNB1
GNAT1
GNGT1
NRL
SAG
DDC
PDE6A
ROM1
RHO
PDE6G
CNGA1
NR2E3
ProA330-tdTomato
scaled expr.
1
0.5
0
Type
Cones
Rods
Condition
Low glucose
Normal glucose
B
Ratio of S-cone (OPNS1W) and L/M-cones (THRB) markers expression
0.65
0.60
0.55
0.50
0.45
Low glucose
Normal glucose
C
Type
Condition
HSP90AA1
HSP90AB1
MTOR
PIK3CA
PIK3CB
PIK3CD
PDGFRA
PDGFRB
log2 (TP10k + 1)
10
5
0
Type
Cones
Rods
Condition
Low glucose
Normal glucose
HSP90AA1−I−1
CS−KI−1
CS−KI−1A
CS−KI−2
CS−KI−2A
Type
Condition
HSP90AA1
HSP90AB1
MTOR
PIK3CA
PIK3CB
PIK3CD
PDGFRA
PDGFRB

**Figure S12: Compound target and marker gene expression, related to Figure 6.**

**A:** Expression of cone (top) and rod (bottom) marker genes. Heatmap colors correspond to gene expression normalized to the row-wise maximum. Normal- and low-glucose conditions are indicated at the top. **B:** Ratio of S-cone (*OPNS1W*) and L/M-cones (*THRB*) markers expression in each organoid exposed to low glucose or normal glucose. **C:** Expression of genes encoding annotated compound targets. Colors at the top indicate cell types and conditions. Color scale indicates gene expression levels. TP10k, transcript counts per 10,000.

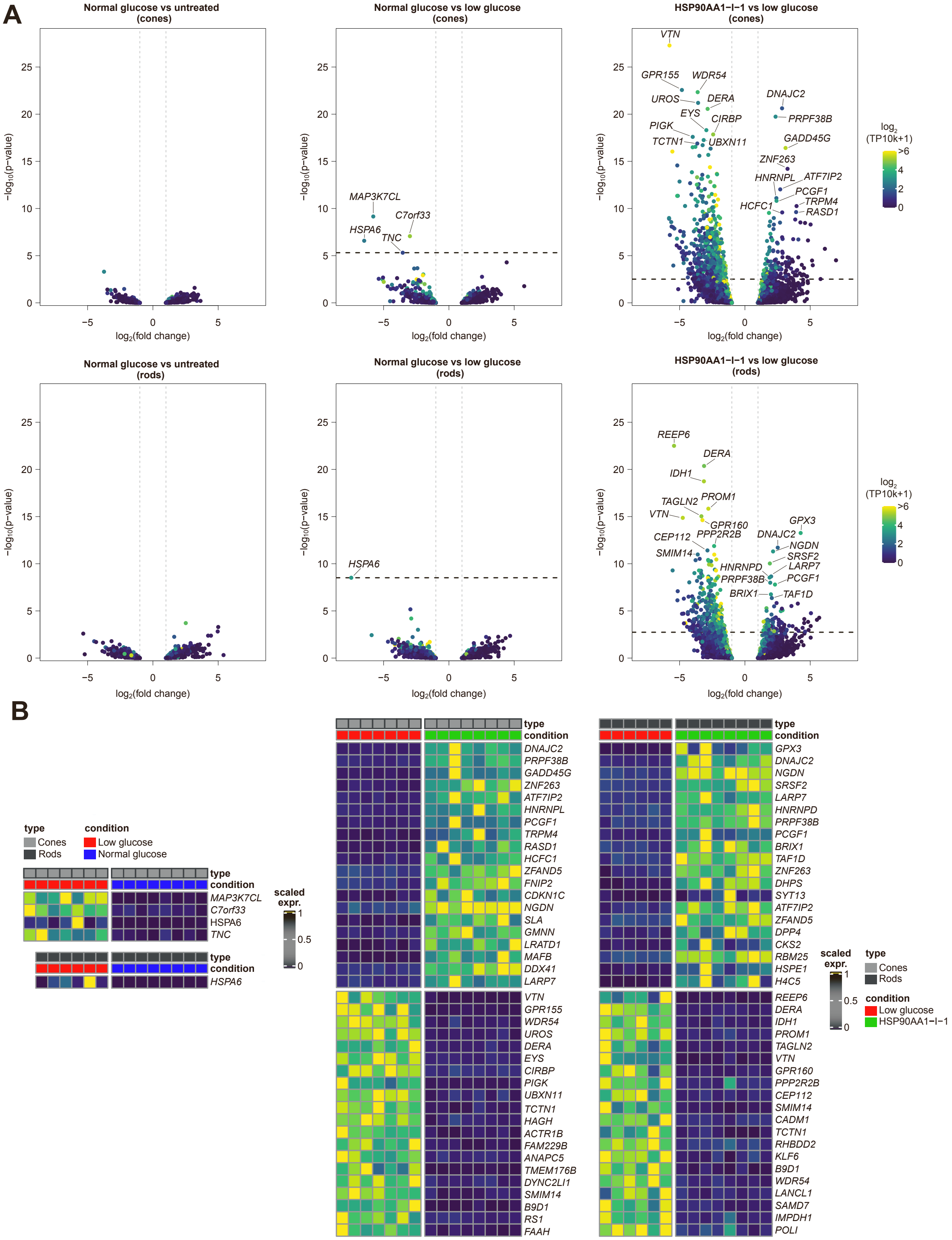
A
Normal glucose vs untreated (cones)
Normal glucose vs low glucose (cones)
HSP90AA1−I−1 vs low glucose (cones)
−log10(p−value)
log2(fold change)
MAP3K7CL
C7orf33
HSPA6
TNC
VTN
GPR155
WDR54
UROS
DERA
EYS
CIRBP
PIGK
TCTN1
UBXN11
DNAJC2
PRPF38B
GADD45G
ZNF263
HNRNPL
ATF7IP2
PCGF1
TRPM4
HCFC1
RASD1
log2 (TP10k+1)
>6
Normal glucose vs untreated (rods)
Normal glucose vs low glucose (rods)
HSP90AA1−I−1 vs low glucose (rods)
REEP6
IDH1
TAGLN2
PROM1
GPR160
PPP2R2B
CEP112
SMIM14
GPX3
NGDN
SRSF2
HNRNPD
LARP7
PRPF38B
PCGF1
BRIX1
TAF1D
B
type
Cones
Rods
condition
Low glucose
Normal glucose
HSP90AA1−I−1
scaled expr.
DNAJC2
PRPF38B
GADD45G
ZNF263
ATF7IP2
HNRNPL
PCGF1
TRPM4
RASD1
HCFC1
ZFAND5
FNIP2
CDKN1C
NGDN
SLA
GMNN
LRATD1
MAFB
DDX41
LARP7
VTN
GPR155
WDR54
UROS
DERA
EYS
CIRBP
PIGK
UBXN11
TCTN1
HAGH
ACTR1B
FAM229B
ANAPC5
TMEM176B
DYNC2LI1
SMIM14
B9D1
RS1
FAAH
GPX3
DNAJC2
NGDN
SRSF2
LARP7
HNRNPD
PRPF38B
PCGF1
BRIX1
TAF1D
ZNF263
DHPS
SYT13
ATF7IP2
ZFAND5
DPP4
CKS2
RBM25
HSPE1
H4C5
REEP6
DERA
IDH1
PROM1
TAGLN2
VTN
GPR160
PPP2R2B
CEP112
SMIM14
CADM1
TCTN1
RHBDD2
KLF6
B9D1
WDR54
LANCL1
SAMD7
IMPDH1
POLI

**Figure S13: Differential gene expression analysis of controls and HSP90AA1-I-1, related to Figure 6.**

**A:** Differential gene expression comparing human retinal organoid cone (top) and rod (bottom) transcriptomes in normal glucose with untreated controls (left), normal glucose to low glucose (middle), and HSP90AA1-I-1 treatment in low glucose to low glucose (right). Gray dotted lines indicate foldchange threshold beyond which genes were included in analysis. Red dotted line indicates the p-value beyond which genes passed the significant threshold of $p<0.05$ after Benjamini-Hochberg correction for multiple testing. Colors indicate average gene expression in low-glucose cones (top) or rods (bottom). The 10 most significantly up- or downregulated genes are labeled. TP10k, transcript counts per 10,000. **B:** Heatmaps displaying gene expression levels of differentially expressed genes across samples under specified conditions. Up to 20 up- and downregulated genes are included and ordered by p-values with the most significant p-value at the top. Heatmap colors correspond to gene expression normalized to the row-wise maximum.

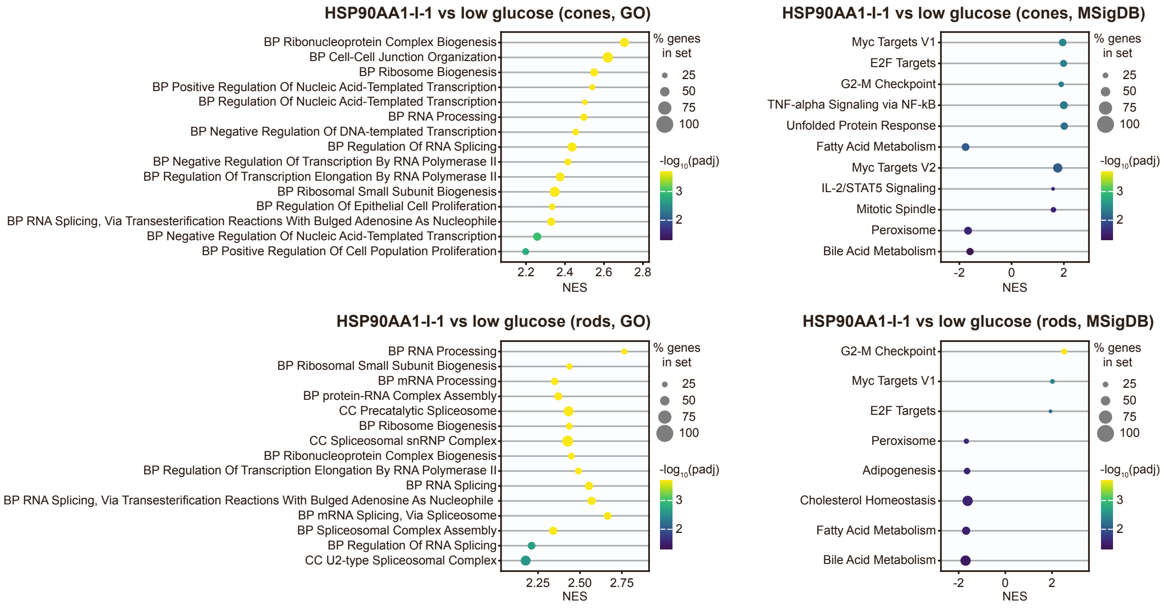


**Figure S14: Gene set enrichment analysis for HSP90AA1-I-1, related to Figure 6.**

Gene Ontology terms (GO, left) and Molecular Signatures Database Terms (MSigDB, right) enrichment analysis of HSP90AA1-I-1 -treated samples compared to low glucose in both cones (top) and rods (bottom). Dots represent normalized enrichment scores (NES) with their size indicating the percentage of differentially expressed genes within each gene set, and the color reflecting the adjusted p-value corrected for multiple testing (padj). All or the top 15 GO-terms are shown ordered by p-values, whereas all enriched MSigDB-terms are shown. BP: biological process, CC: cellular component.

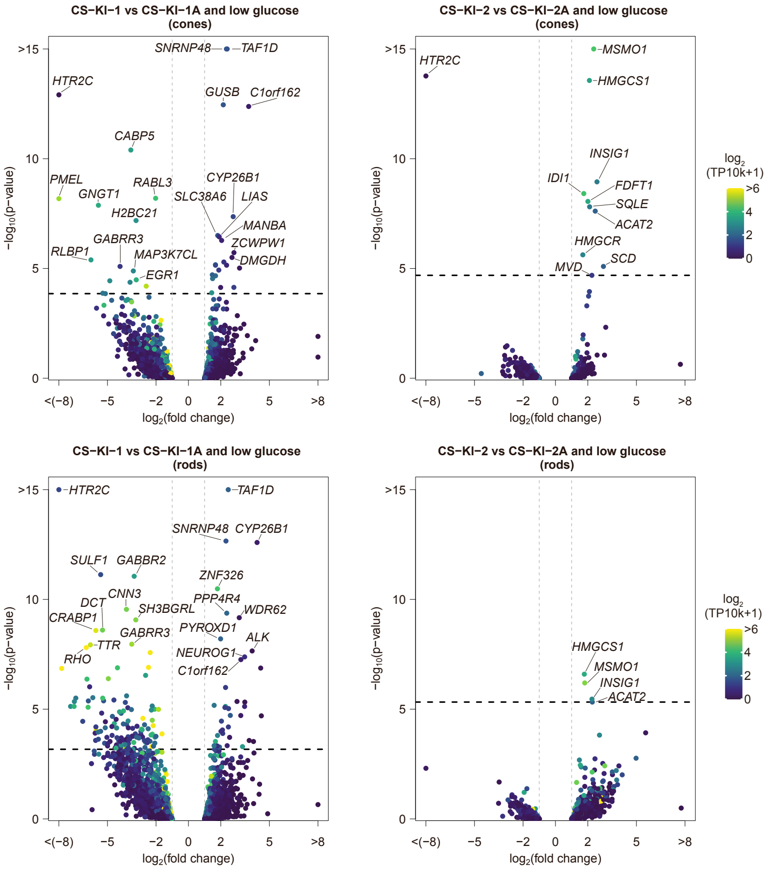


**Figure S15: Differential gene expression analysis for cone-saving compounds, related to Figure 6.** Differential gene expression comparing human retinal organoid cones (top) and rods (bottom) transcriptomes treated with CS-KI-1 compared to CS-KI-1A treatment and low-glucose controls (left), and CS-KI-2 treatment compared to CS-KI-2A treatment and low-glucose controls (right). Gray dotted lines indicate the threshold beyond which genes were included in analysis. Red dotted line indicates the p-value beyond which genes passed the significant threshold of $p<0.05$ after Benjamini-Hochberg correction for multiple testing. Colors indicate average gene expression in low-glucose cones (top) or rods (bottom). Up to 10 most significantly up- or downregulated genes are labeled. TP10k, transcript counts per 10,000.

A

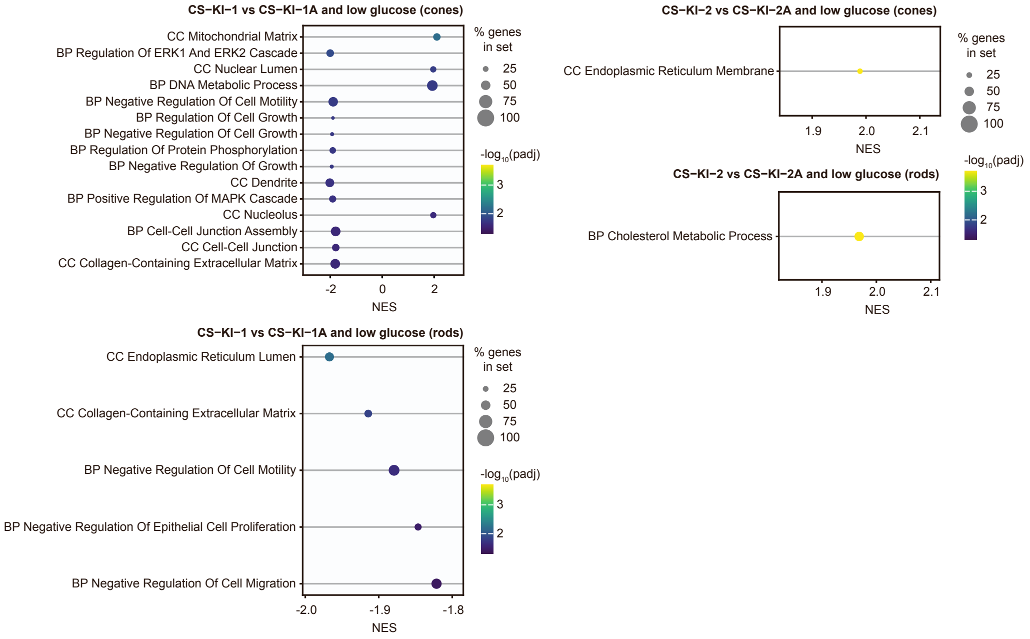
CS−KI−1 vs CS−KI−1A and low glucose (cones)
CC Mitochondrial Matrix
BP Regulation Of ERK1 And ERK2 Cascade
CC Nuclear Lumen
BP DNA Metabolic Process
BP Negative Regulation Of Cell Motility
BP Regulation Of Cell Growth
BP Negative Regulation Of Cell Growth
BP Regulation Of Protein Phosphorylation
BP Negative Regulation Of Growth
CC Dendrite
BP Positive Regulation Of MAPK Cascade
CC Nucleolus
BP Cell-Cell Junction Assembly
CC Cell-Cell Junction
CC Collagen-Containing Extracellular Matrix
-2
0
2
NES
% genes in set
25
50
75
100
-log10(padj)
3
2
CS−KI−2 vs CS−KI−2A and low glucose (cones)
CC Endoplasmic Reticulum Membrane
1.9
2.0
2.1
NES
CS−KI−2 vs CS−KI−2A and low glucose (rods)
BP Cholesterol Metabolic Process
1.9
2.0
2.1
NES
CS−KI−1 vs CS−KI−1A and low glucose (rods)
CC Endoplasmic Reticulum Lumen
CC Collagen-Containing Extracellular Matrix
BP Negative Regulation Of Cell Motility
BP Negative Regulation Of Epithelial Cell Proliferation
BP Negative Regulation Of Cell Migration
-2.0
-1.9
-1.8
NES

B

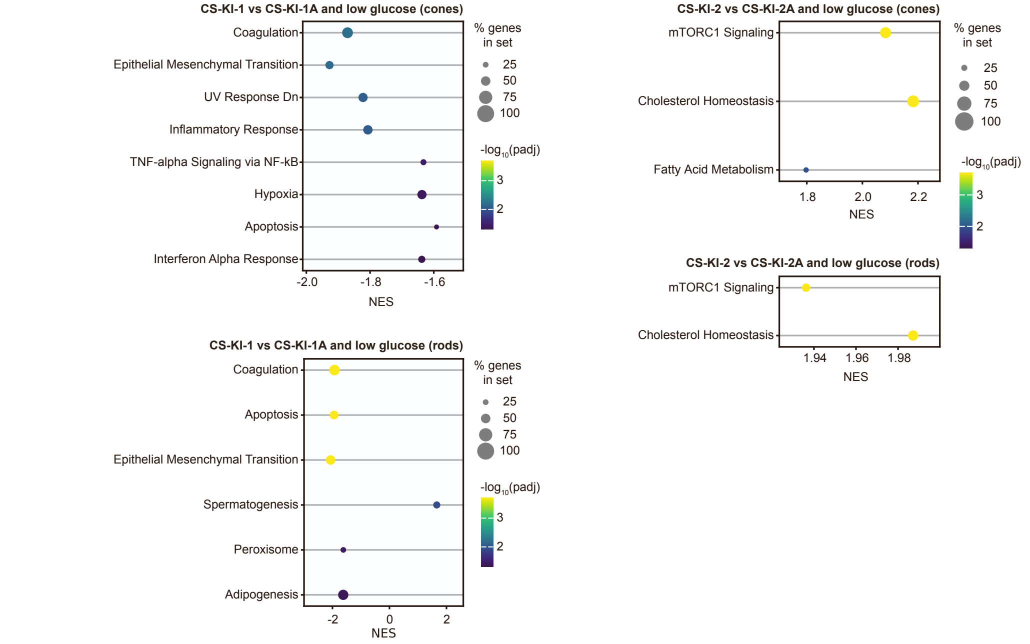
CS-KI-1 vs CS-KI-1A and low glucose (cones)
Coagulation
Epithelial Mesenchymal Transition
UV Response Dn
Inflammatory Response
TNF-alpha Signaling via NF-kB
Hypoxia
Apoptosis
Interferon Alpha Response
-2.0
-1.8
-1.6
NES
% genes in set
25
50
75
100
-log10(padj)
3
2
CS-KI-2 vs CS-KI-2A and low glucose (cones)
mTORC1 Signaling
Cholesterol Homeostasis
Fatty Acid Metabolism
1.8
2.0
2.2
NES
CS-KI-2 vs CS-KI-2A and low glucose (rods)
mTORC1 Signaling
Cholesterol Homeostasis
1.94
1.96
1.98
NES
CS-KI-1 vs CS-KI-1A and low glucose (rods)
Coagulation
Apoptosis
Epithelial Mesenchymal Transition
Spermatogenesis
Peroxisome
Adipogenesis
-2
0
2
NES

**Figure S16: Gene set enrichment analysis for cone-saving compounds, related to Figure 6.**

**A-B:** Gene Ontology (GO) terms enrichment analysis (A) and Molecular Signatures Database (MSigDB) terms enrichment analysis (B) for specified comparisons in both cones (top) and rods (bottom). Dots represent normalized enrichment scores (NES) with their size indicating the percentage of differentially expressed genes within each gene set, and the color reflecting the adjusted p-value corrected for multiple testing (padj). All or the top 15 GO terms (A) or MSigDB terms (B) with the most significant p-values are displayed ordered by p-values.

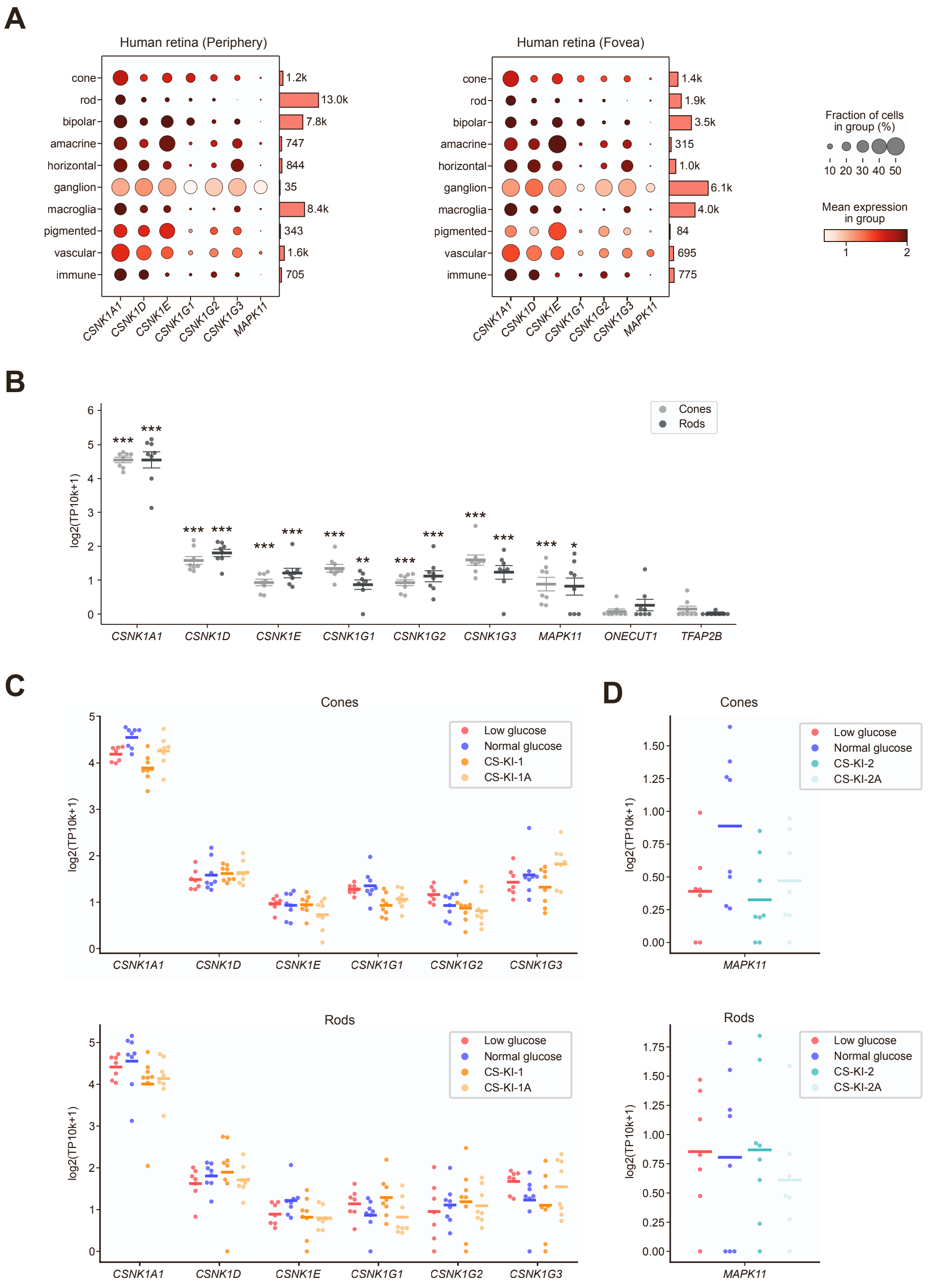
A
Human retina (Periphery)
Human retina (Fovea)
cone
rod
bipolar
amacrine
horizontal
ganglion
macroglia
pigmented
vascular
immune
1.2k
13.0k
7.8k
747
844
35
8.4k
343
1.6k
705
1.4k
1.9k
3.5k
315
1.0k
6.1k
4.0k
84
695
775
CSNK1A1
CSNK1D
CSNK1E
CSNK1G1
CSNK1G2
CSNK1G3
MAPK11
Fraction of cells in group (%)
10 20 30 40 50
Mean expression in group
1
2
B
Cones
Rods
log2(TP10k+1)
***
**
*
CSNK1A1
CSNK1D
CSNK1E
CSNK1G1
CSNK1G2
CSNK1G3
MAPK11
ONECUT1
TFAP2B
C
Cones
Rods
Low glucose
Normal glucose
CS-KI-1
CS-KI-1A
log2(TP10k+1)
D
Low glucose
Normal glucose
CS-KI-2
CS-KI-2A
MAPK11

**Figure S17: Expression of casein kinase 1 genes and MAPK11 in human retina and organoids, related to Figure 7.**

**A:** *CK1* and *MAPK11* gene expression in human retina fovea and periphery from published single cell atlases [1]. Color indicates mean expression in $log_2$ transcript counts per 10000. Dot size indicates the fraction of cells expressing the indicated gene. Right bars and numbers indicate the number of cells from each population used for the analysis. **B:** *CK1* gene family and *MAPK11* differential gene expression in rods and cones in normal-glucose organoids from data generated in this study. *ONECUT1* and *TFAP2B* are shown as examples of genes not expressed in photoreceptors and their combined expression is used for statistical comparison with the *CK1* and *MAPK11* genes (significantly expressed genes are marked with *, denoting a p-value<0.05, ** when p-value<0.01 and *** when p-value<0.001, after one-way ANOVA with Dunnett's correction for multiple testing). The results are shown as mean ± se and each dot corresponds to the expression in one organoid. **C-D:** *CK1* family genes and *MAPK11* differential gene expression in rods and cones from organoids treated with low glucose, normal glucose and selected compounds and their analogs. Dots represent individual organoids, and mean is shown with a line. TP10k, transcript counts per 10,000.

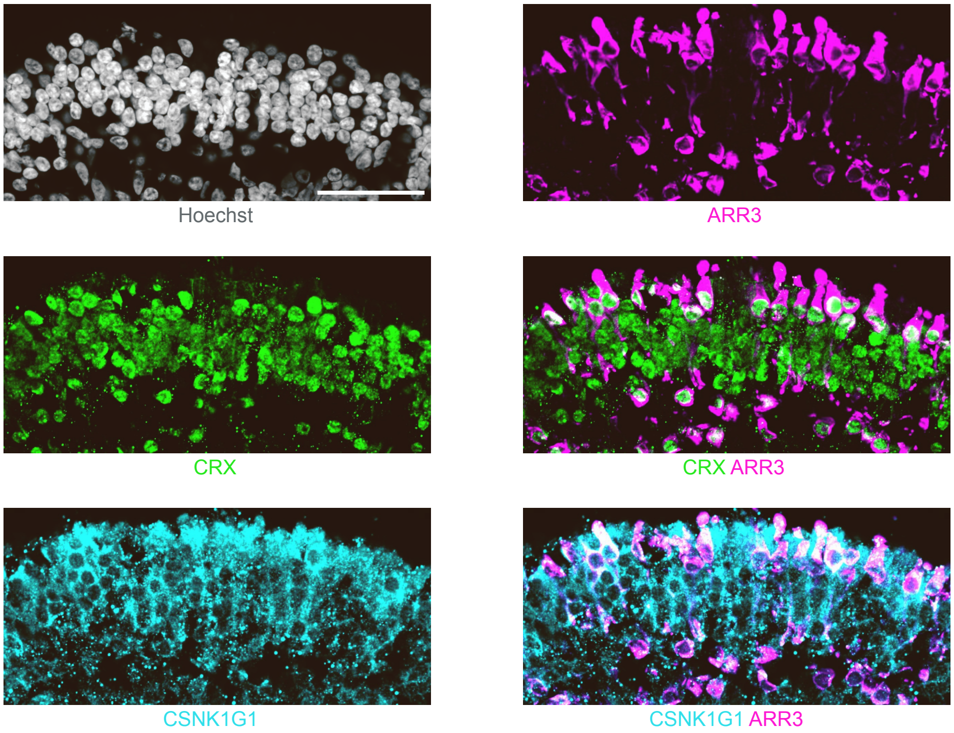


**Figure S18: CSNK1G1 protein detection in organoids, related to Figure 7.**
Confocal images of sectioned and stained human retinal organoids at week 30 of maturation with indicated antibodies for the detection of CSNK1G1 protein, rods and cones (CRX) and cones (ARR3). Hoechst labels nuclei (scale bar, 50 μm).

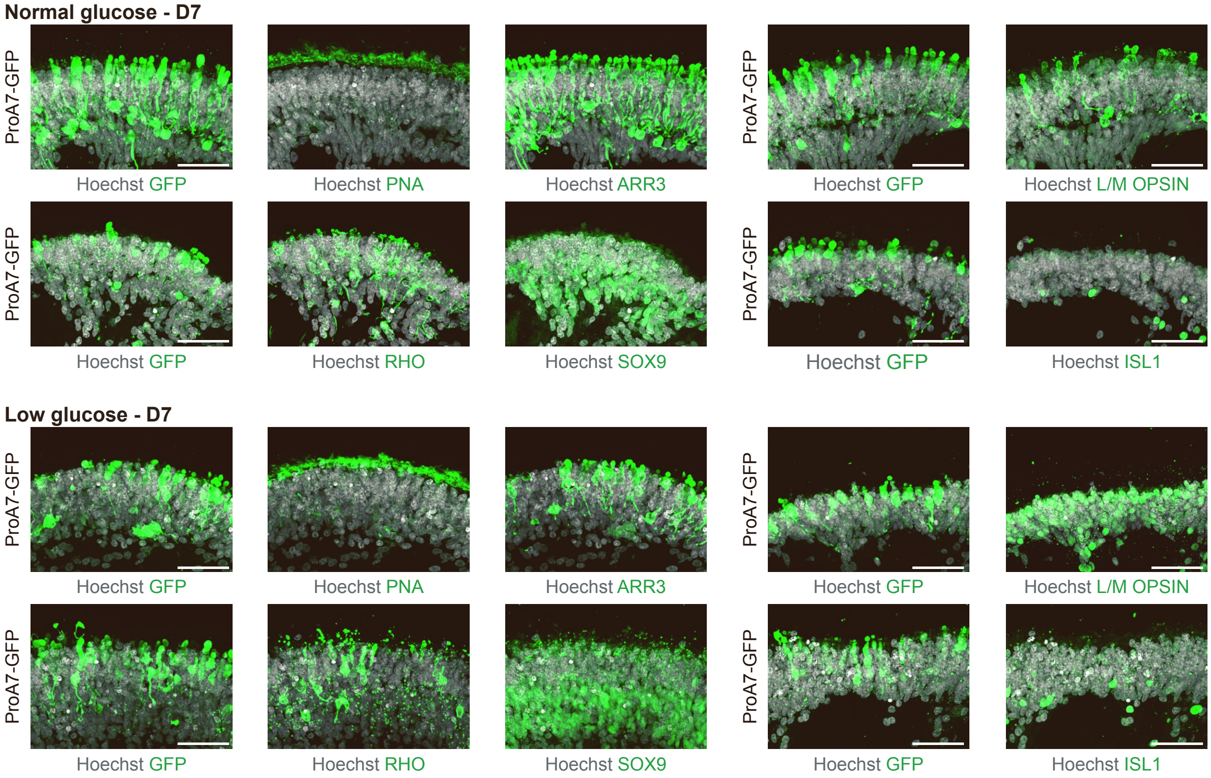


**Figure S19: Photoreceptor morphology and presence of other cell types in treated organoids with normal and low glucose, related to Figure 7.**

Confocal images of sectioned and stained human retinal organoids transduced with ProA7-GFP AAVs targeting cones. Indicated antibody stainings show the morphology and structure of rods (RHO) and cones (ARR3, L/M OPSIN, PNA labeling outer segments), and the presence and distribution of Muller (SOX9) and a subset of bipolar and horizontal cells (ISL1) in human retinal organoids in normal glucose and low glucose at D7. Hoechst labels nuclei. The corresponding image of transduced cones ($GFP^{+}$) for each section used for the staining was included as a reference (scale bar, 50 μm).

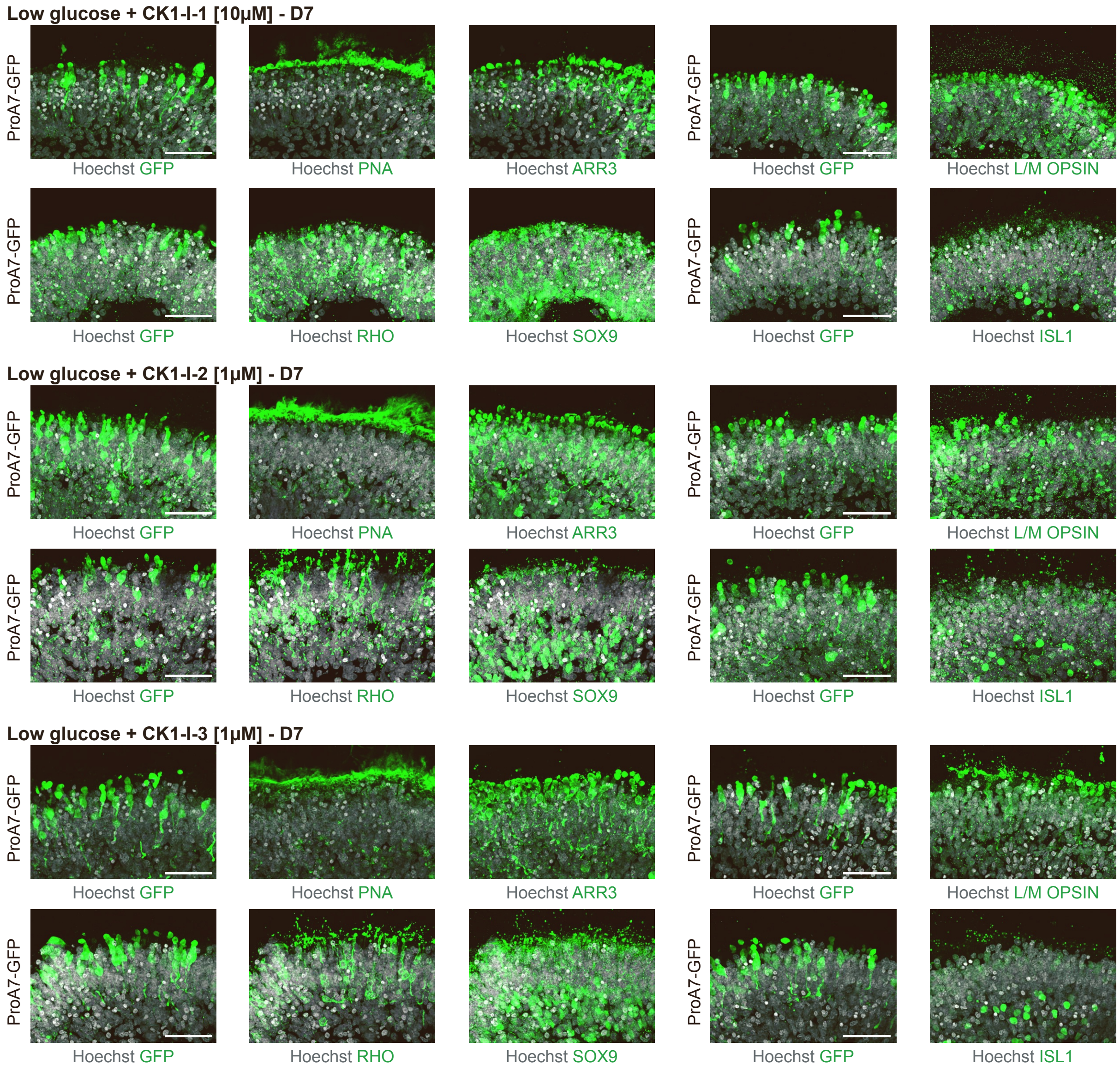


**Figure S20: Photoreceptor morphology and presence of other cell types in treated organoids with CK1 inhibitors, related to Figure 7.**

Confocal images of sectioned and stained human retinal organoids transduced with ProA7-GFP AAVs targeting cones. Indicated antibody stainings show the morphology and structure of rods (RHO) and cones (ARR3, L/M OPSIN, PNA labeling outer segments), and the presence and distribution of Muller (SOX9) and a subset of bipolar and horizontal cells (ISL1) in human retinal organoids treated with CK1 inhibitors at the indicated concentrations in low glucose at D7. Hoechst labels nuclei. The corresponding image of transduced cones ($GFP^+$) for each section used for the staining was included as a reference (scale bar, 50 µm).

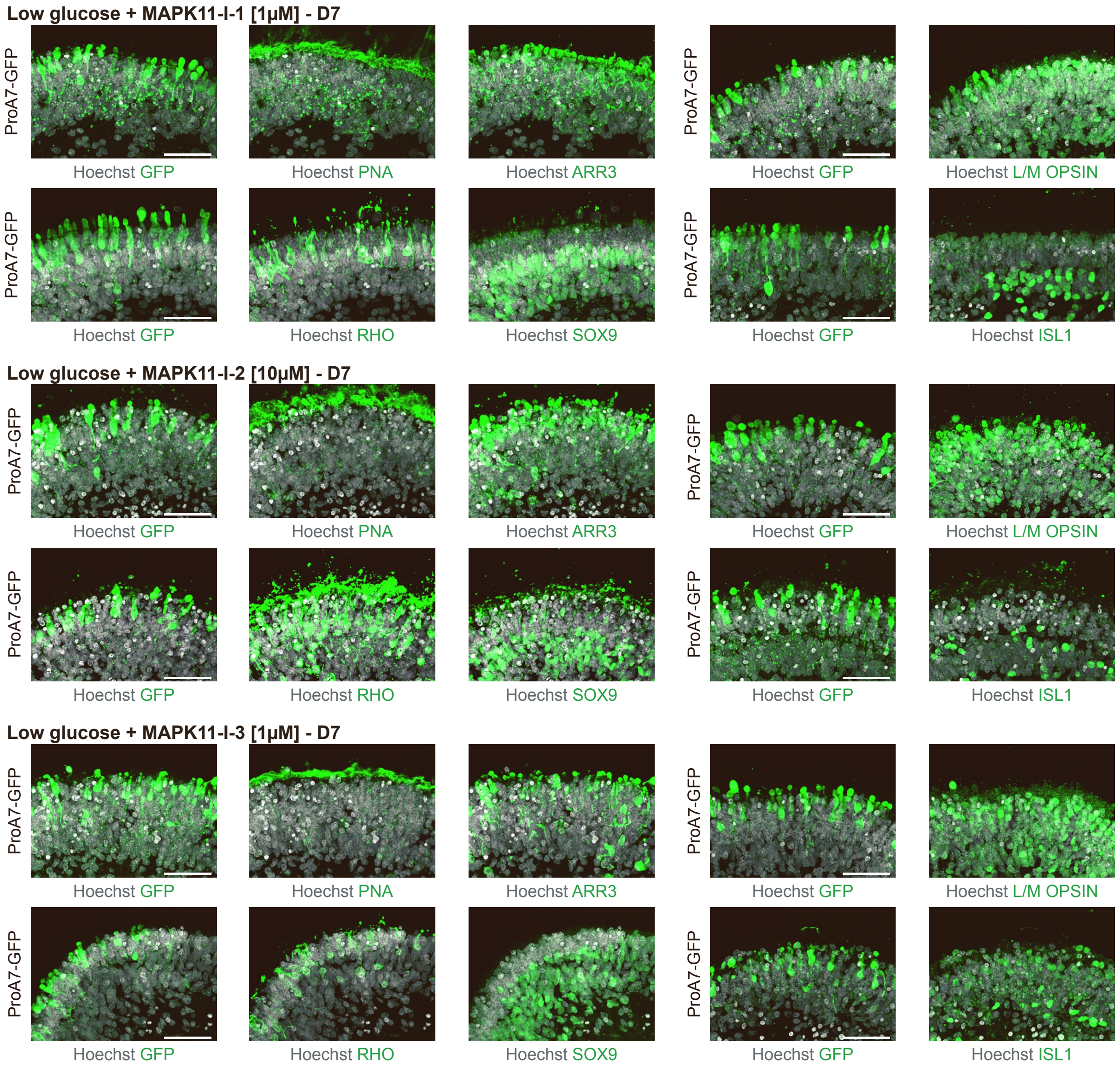


**Figure S21: Photoreceptor morphology and presence of other cell types in treated organoids with MAPK11 inhibitors, related to Figure 7.**

Confocal images of sectioned and stained human retinal organoids transduced with ProA7-GFP AAVs targeting cones. Indicated antibody stainings show the morphology and structure of rods (RHO) and cones (ARR3, L/M OPSIN, PNA labeling outer segments), and the presence and distribution of Muller (SOX9) and a subset of bipolar and horizontal cells (ISL1) in human retinal organoids treated with MAPK11 inhibitors at the indicated concentrations in low glucose at D7. Hoechst labels nuclei. The corresponding image of transduced cones ($GFP^+$) for each section used for the staining was included as a reference (scale bar, 50 μm).

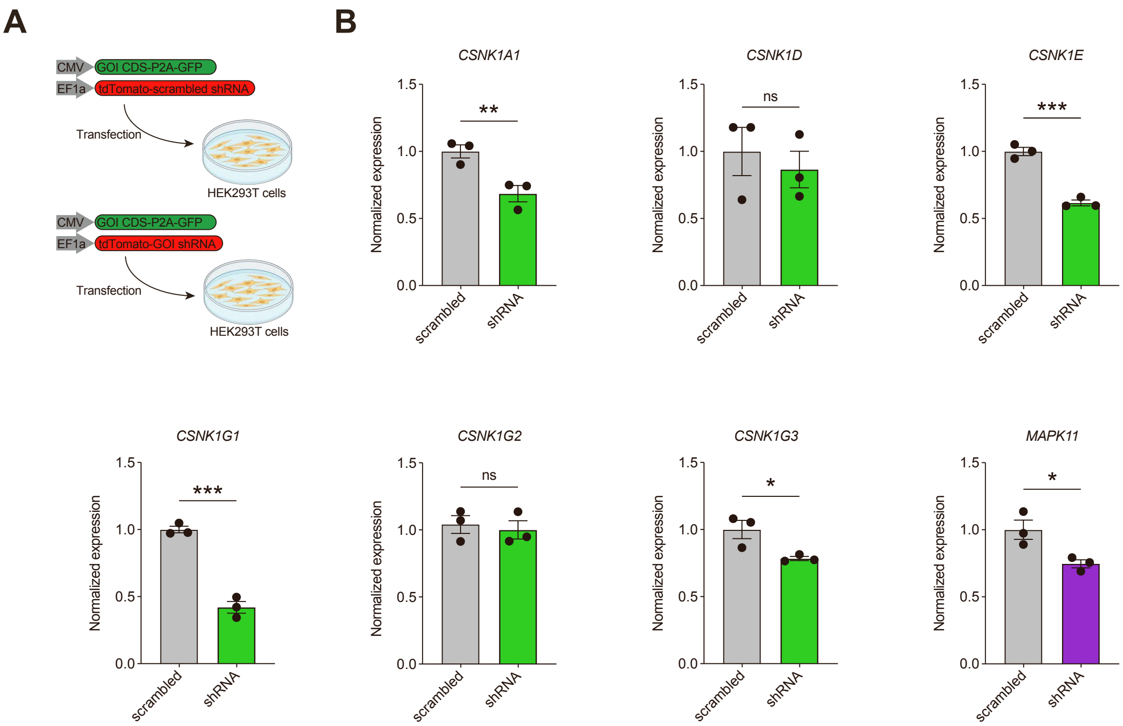


**Figure S22: Validation of shRNAs in HEK293T cells, related to Figure 7.**

**A:** Schematic illustration for the validation of shRNAs in HEK293T cells. Coding sequence and shRNAs (using scrambled shRNAs as control) of the target genes were overexpressed simultaneously in HEK293T cells and the expression of the targeted genes was determined. GOI CDS: Gene of interest coding sequence. **B:** Normalized relative values of expression of the targeted genes. The results are shown as mean ± se with * denoting a p-value<0.05, and each black dot represents an independent culture.

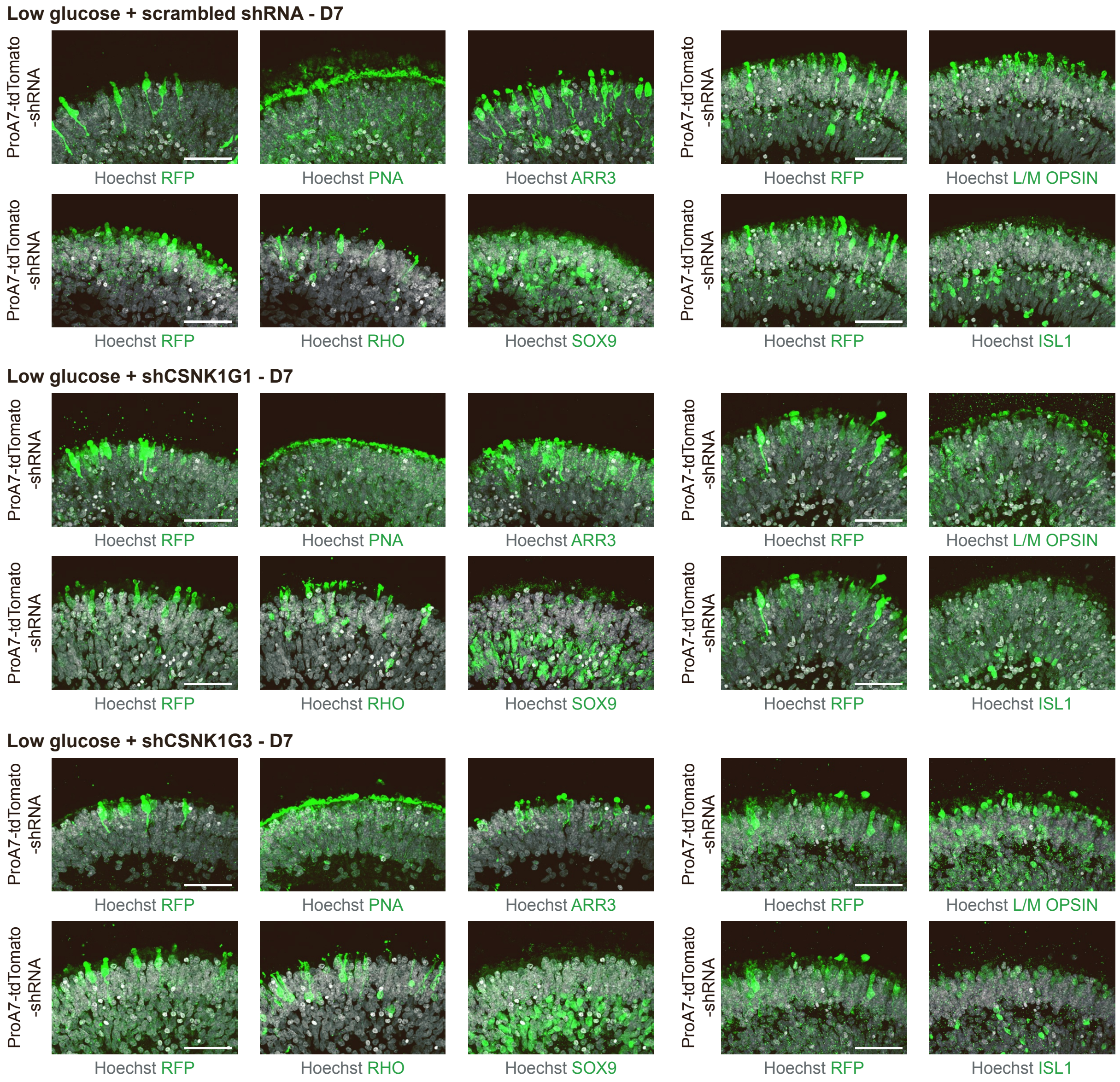


**Figure S23: Photoreceptor morphology and presence of other cell types in treated organoids with shRNAs, related to Figure 7.**

Confocal images of sectioned and stained human retinal organoids transduced with AAVs encoding ProA7-tdTomato-shRNA targeting cones. Indicated antibody stainings show the morphology and structure of rods (RHO) and cones (ARR3, L/M OPSIN, PNA labeling outer segments), and the presence and distribution of Muller (SOX9) and a subset of bipolar and horizontal cells (ISL1) in human retinal organoids treated with scrambled shRNAs, or shRNAs for *CSNK1G1* or *CSNK1G3* at D7 after exposure to low glucose. Hoechst labels nuclei. The corresponding image of transduced cones ($GFP^{+}$) for each section used for the staining was included as a reference (scale bar, 50 µm).

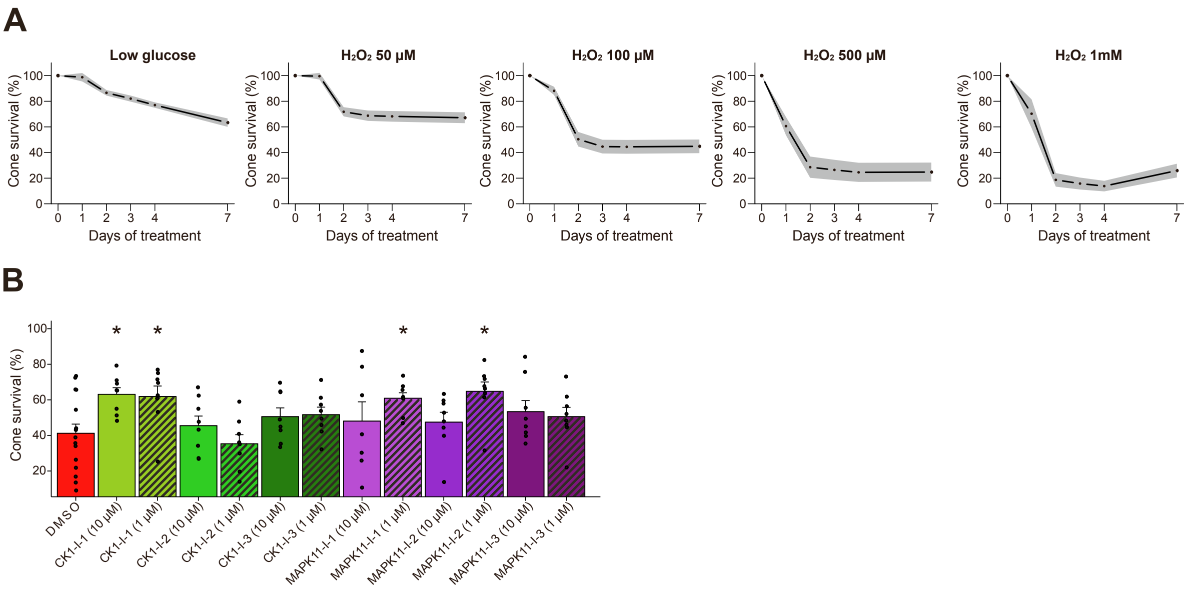


**Figure S24: Compound effects in a model of photoreceptor degeneration induced by oxidative stress, related to Figure 7.**

**A:** Time course of cone survival in organoids exposed to different concentrations of $H_2O_2$. Results are shown as mean ± se. **B:** Cone survival at D7 in organoids exposed to 50 µM $H_2O_2$ and treated with different CK1 and MAPK11 inhibitors. Results are shown as mean ± se with * denoting an adjusted p-value<0.05. Black dots indicate individual organoids.

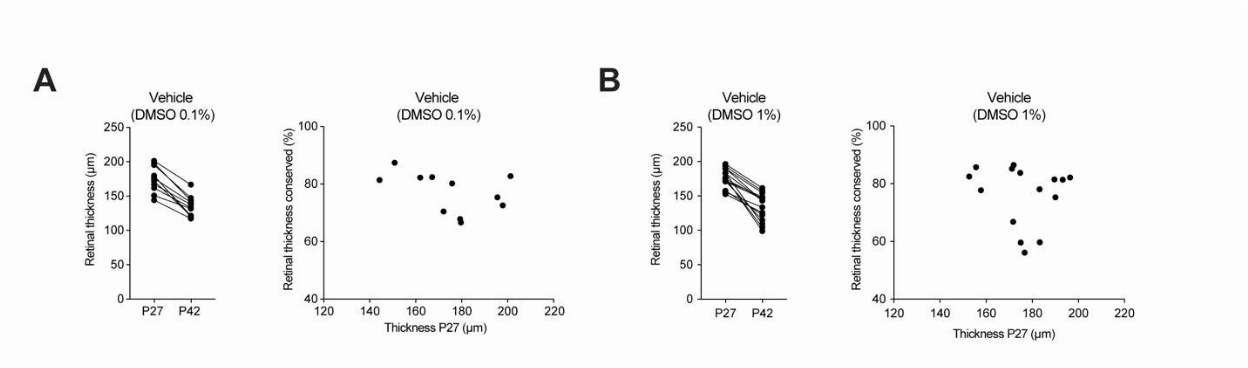


**Figure S25: Analysis of control injected eyes in rd10 mice, related to Figure 7.**

**A:** Left: retinal thickness at P27 and P42 of the control eyes injected with vehicle (DMSO 0.1%). Right: Thickness of control eyes at P27 against the corresponding percentage of retinal conservation between P27 and P42. **B:** Left: retinal thickness at P27 and P42 of the control eyes injected with vehicle (DMSO 1%). Right: Thickness of control eyes at P27 against the corresponding percentage of retinal conservation between P27 and P42. Each dot represents an injected eye.

**Table S6: Custom oligonucleotide sequences, related to the Key Resources Table and the STAR Methods section.**

| Name | Sequence |
|---|---|
| ITR-Fw | GGAACCCCTAGTGATGGAGTT |
| ITR-Rev | CGGCCTCAGTGAGCGA |
| ITR-Probe | CACTCCCTCTCTGCGCGCTCG |
| CSNK1A1_qPCR_Fw | AACCAGATAACTTCCTAATG |
| CSNK1A1_qPCR_Rev | GCACATATTCAATTCTACTG |
| CSNK1D_qPCR_Fw | AAGGATTAGCGAGAAGAA |
| CSNK1D_qPCR_Rev | GGCAGAAATTCAGGTATG |
| CSNK1E_qPCR_Fw | CAGCGAGAAGAAGATGTC |
| CSNK1E_qPCR_Rev | AAGTTGAGGTATGTTGAGAAT |
| CSNK1G1_qPCR_Fw | GGTTGGCAAGAAGATAGG |
| CSNK1G1_qPCR_Rev | ACATATTCATTGGTGTAGAGAT |
| CSNK1G2_qPCR_Fw | AACTTCCCAGAGGAGATG |
| CSNK1G2_qPCR_Rev | TTCCGCAGGTAGTCATAG |
| CSNK1G3_qPCR_Fw | TAATAGGACGACCAGGAA |
| CSNK1G3_qPCR_Rev | GTAAGGCTCTTGTGTTCT |
| MAPK11_qPCR_Fw | GAACAACATCGTCAAGTG |
| MAPK11_qPCR_Rev | CGAGTGGATGTACTTCAG |
| GAPDH_qPCR_Fw | GCACCACCAACTGCTTAG |
| GAPDH_qPCR_Rev | TTCTGGGTGGCAGTGAT |

**Supplemental References**


[1] Cowan, C.S., Renner, M., De Gennaro, M., Gross-Scherf, B., Goldblum, D., Hou, Y., Munz, M., Rodrigues, T.M., Krol, J., Szikra, T., et al. (2020). Cell Types of the Human Retina and Its Organoids at Single-Cell Resolution. Cell 182, 1623-1640.e34. https://doi.org/10.1016/j.cell.2020.08.013.
